# Surveillance of PFAS in sludge and biosolids at 12 water resource recovery facilities

**DOI:** 10.1002/jeq2.20595

**Published:** 2024-07-14

**Authors:** Shubhashini Oza, Katherine Y. Bell, Zhiliang Xu, Yifei Wang, Martha J. M. Wells, John W. Norton, Lloyd J. Winchell, Qingguo Huang, Hui Li

**Affiliations:** ^1^ Brown and Caldwell Charlotte North Carolina USA; ^2^ Brown and Caldwell Nashville Tennessee USA; ^3^ Department of Plant, Soil and Microbial Sciences Michigan State University East Lansing Michigan USA; ^4^ Department of Crop and Soil Science University of Georgia Griffin Georgia USA; ^5^ EnviroChem Services Cookeville Tennessee USA; ^6^ Great Lakes Water Authority Detroit Michigan USA; ^7^ Brown and Caldwell Saint Paul Minnesota USA

## Abstract

Per‐ and polyfluoroalkyl substances (PFAS) are refractory anthropogenic chemicals and current treatment processes at municipal water resource recovery facilities (WRRFs) cannot efficiently degrade them, hence, these chemicals cycle through the environment. Certain PFAS can be concentrated in biosolids from WRRFs and are commonly land applied for beneficial reuse. Given recent advances in measurement of PFAS, documentation of the range of concentrations in pre‐stabilized sludge and stabilized biosolids is critical to evaluating treatment best practices and assessing potential human health and ecological risks. In this study, pre‐stabilized sludge and post‐stabilized biosolids samples were collected from 12 major WRRFs across the United States. PFAS were analyzed using Environmental Protection Agency (EPA) Method SW846‐3500C/537.1, and Draft EPA Method 1633, by one commercial laboratory and two university research laboratories, respectively. Results comparison among laboratories demonstrated statistical differences in PFAS concentrations among split samples. For example, 5:3 FTCA (fluorotelomer carboxylic acid) concentrations in post‐stabilized sludge at Lab 1 were measured at 21 ng/g (dry), while they were detected at 151 ng/g (dry) in Lab 3. Further, higher PFAS concentrations were observed in post‐stabilized biosolids compared to pre‐stabilized sludges, regardless of the laboratory or analysis method, even when solids destruction through solids stabilization was considered. Further research is required to refine methods for analyses of PFAS in sludge and biosolids samples from WRRFs prior to being used for development of regulatory actions as well as understanding how various treatment protocols could impact concentrations of PFAS in land‐applied biosolids.

## Core Ideas

- The study documents the range of concentrations of 40 per‐ and polyfluoroalkyl substances (PFAS) present in solids from 12 water resource recovery facilities in the United States, which may be used to inform development of regulatory policies.
- PFAS were measured in pre‐stabilized sludge and post‐stabilized biosolids using two different Environmental Protection Agency Methods.
- Measured PFAS were significantly different among the three laboratories.
- Higher 5:3 FTCA and PFOS concentrations were found in post‐stabilized biosolids than in pre‐stabilized sludges.

**Abbreviations**
FTCA: fluorotelomer carboxylic acid
FTOS: fluorotelomer sulfonic acid
IDA: isotope dilution analyte
LC‐MS/MS: liquid chromatography‐tandem mass spectrometry
MDL: minimum detection limit
NSSS: National Sewage Sludge Survey
PCB: polychlorinated biphenyl
PFAS: per‐ and polyfluoroalkyl substances
PFOA: perfluorooctanoic acid
PFOS: perfluorooctane sulfonate
QA: quality assurance
QC: quality control
RL: reporting limit
SPE: solid‐phase extraction
US EPA: United States Environmental Protection Agency
WAS: waste activated sludge
WRRF: water resource recovery facility

## 1 INTRODUCTION

Recalcitrant compounds such as dioxins, furans, and polychlorinated biphenyls (PCBs) have been introduced into the environment, with resultant impacts on human health and ecosystems (Barone et al., 2021). Banning PCB production and use, and regulation of discharges to water, air, and soil is history being repeated with yet another class of compounds—per‐ and polyfluoroalkyl substances (PFAS). PFAS are a family of per‐ and polyfluorinated chemicals “that structurally contain the unit R‐(CF_2_)–C(F)(R′) R″. Both the CF_2_ and CF moieties are saturated carbons and none of the R groups (R, R′, or R″) can be hydrogen” (US EPA, 2023d). They are used to make coatings and products that resist heat, oil, stains, grease, and water, and have been produced since the late 1940s (Panieri et al., 2022). The unique chemistry of the carbon–fluorine bond results in strong, stable, and water‐ and oil‐repellent characteristics compared to their hydrocarbon counterparts (Wang et al., 2017). Thus, numerous PFAS compounds have been developed for industrial and consumer applications such as cosmetics, fire‐fighting foams, food contact materials, household products, inks, medical devices, oil production, mining, pesticides, textile, leather, and apparel (Gaines, 2023).

Given their widespread use, PFAS have been released into the environment and found in surface, ground waters, soils, and air (Phong Vo et al., 2020). Due to their resistance to degradation, they cycle through the environment (Winchell et al., 2022), and studies have shown that they are not efficiently removed in municipal water resource recovery facilities (WRRFs) (Arvaniti & Stasinakis, 2015; Chen et al., 2018; Szabo et al., 2018). Further, PFAS are known to partition to biosolids (Choi et al., 2019; Guo et al., 2010; Schaefer et al., 2022), which are commonly land applied for beneficial reuse. The term biosolids, here, refers to stabilized solids produced at WRRFs, where the United States Environmental Protection Agency (US EPA) defines biosolids as “a product of the wastewater treatment process. During wastewater treatment, the liquids are separated from the solids. Those solids are then treated physically and chemically to produce a semisolid, nutrient‐rich product known as biosolids. The terms ‘biosolids’ and ‘sewage sludge’ are often used interchangeably” (US EPA, 2023a). While the practice of land application is an important means of sustainably managing carbon and associated nutrients, studies have shown that biosolids containing PFAS have resulted in contamination of soil, ground, and surface waters in specific cases (Lindstrom et al., 2011; Sepulvado et al., 2011). Considering biosolids as a potential source of PFAS in land application schemes, the US EPA conducted the first National Sewage Sludge Survey in 2001, documenting 13 PFAS compounds (Venkatesan & Halden, 2013).

Since the 2001 EPA study, advances in analytical chemistry have expanded the list of PFAS, including lower detection limits and optimized extraction methods. The Draft Method 1633 (US EPA, 2023) for PFAS analysis includes low‐level (part per trillion) analyses for forty compounds and incorporates the quality control (QC) acceptance criteria for all aqueous matrices (surface water, ground water, and wastewater). The final version of Method 1633 was published in January 2024 (after this study had been conducted) and included QC acceptance guidance for all eight environmental matrices (wastewater, surface water, groundwater, soil, biosolids, sediment, landfill leachate, and fish tissue), derived from a multi‐laboratory validation study. In addition to improved analytical methods, more powerful risk assessment techniques are available than when PCBs, dioxins, and furans were identified as persistent environmental contaminants that pose risks (Hites, 2011). The historical themes of risk identification, bans on production and use, and broad regulation of toxic, recalcitrant compounds could inform the emerging PFAS story (Al Amin et al., 2020). Thus, as regulators advance policy, laws, monitoring, and reporting requirements for PFAS, it is critical to develop data using appropriate sampling and analytical methods, QC, and interpretation of low‐level data.

The increasing detection of ever‐lower concentrations of PFAS, combined with new data on potential risks to human and ecological health, has prompted the US EPA to evaluate risk to inform regulatory policy and criteria for PFAS emissions (US EPA, 2021). The US EPA is completing a risk assessment for perfluorooctanoic acid (PFOA) and perfluorooctane sulfonate (PFOS) in biosolids, paving the way for future rulemaking. In context of this effort, the US EPA has funded research to improve the knowledge of the occurrence, transport, fate, plant uptake, bioaccumulation in livestock, and human exposure to PFAS in land‐based biosolids application (Oza et al., 2023). Thus, development of accurate PFAS data in biosolids is critical to informing appropriate policy and regulations that will guide selection and implementation of solids treatment practices. Noting, only minor differences between the fourth Draft and final versions of EPA Method 1633 (published after this work was completed), including, among other changes, elimination of QC samples: field duplicates, matrix spikes, and laboratory duplicates. Whether data quality is improved with EPA Method 1633 compared to modified EPA Method 537.1 preceded by clean up remains to be documented. The commercial laboratory used in this study has developed internal laboratory data demonstrating that comparable results can be generated using both methods. Notably, some drawbacks to the wastewater industry associated with EPA Method 1633 includes higher cost, potentially longer sample turnaround times, and the limitation to only 40 PFAS compounds. Conversely, the modified EPA Method SW846‐3550C/537.1 method supports slightly lower reporting for certain PFAS, with support for over 70 PFAS compounds (US EPA, 2007).

In this study, sludge from primary and secondary treatment processes, pre‐stabilization (Location A) and post‐stabilization (biosolids) samples (Location B), was collected from 12 WRRFs throughout the United States. Samples were analyzed by three different laboratories, two university research laboratories, and one commercial laboratory. While other studies have conducted interlaboratory methods comparisons for analyses of PFAS in water (Whitaker et al., 2021), this is the first to examine the sample extraction and analysis methods for split samples of sludge and biosolids to document the range of PFAS concentrations across multiple facilities.

## 2 MATERIALS AND METHODS

**FIGURE 1:**
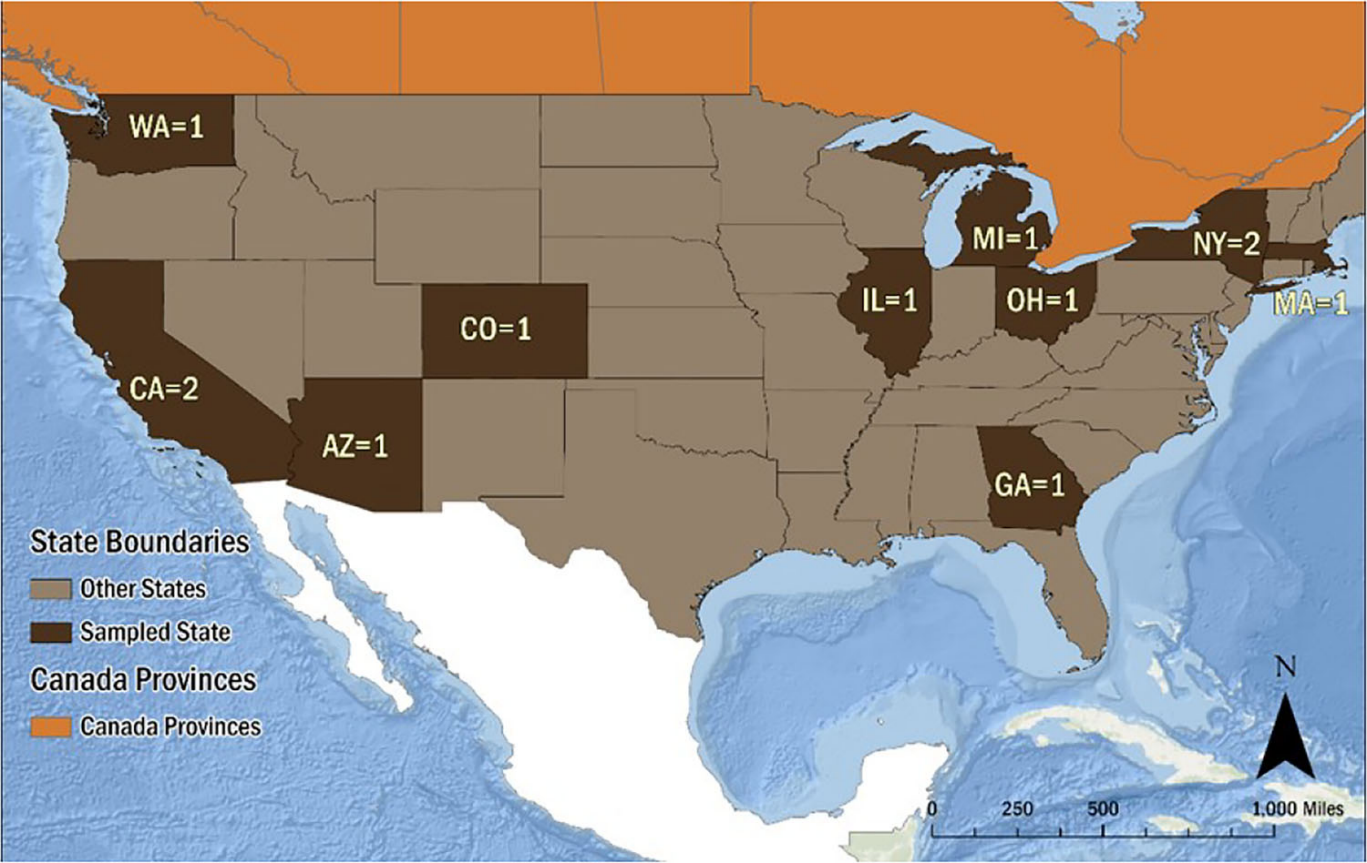
Geographical locations of 12 WRRFs in this study.

**FIGURE 2:**
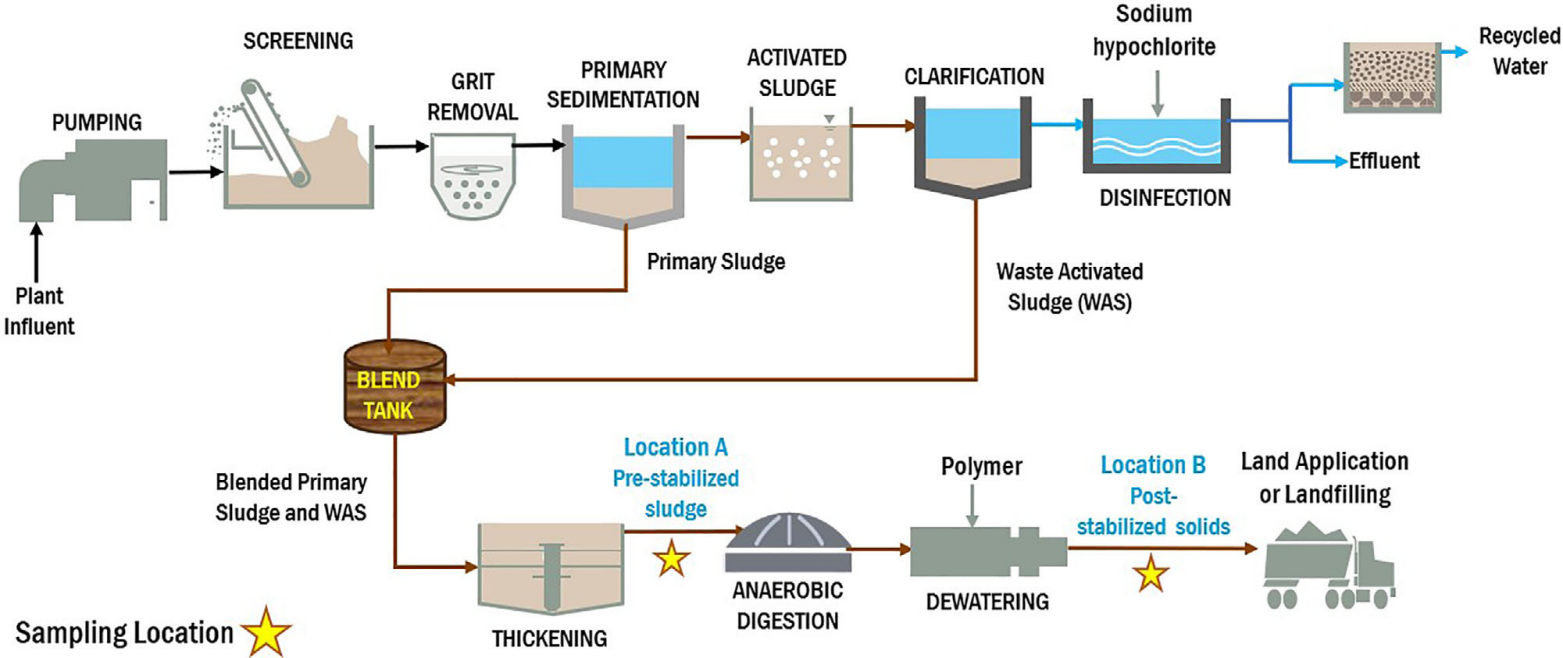
Sample schematic of WRRF indicating sample locations A and B.

The 12 WRRFs (Figure 1) in this study had slightly different treatment processes; therefore, the pre‐stabilized sludge (Location A) and post‐stabilized biosolids (Location B) samples (Figure 2) have different characteristics. Weemaes and Verstraete previously reported that primary sludge has higher total solids compared to waste activated sludge (WAS) from secondary clarifiers. Both types of sludge contain nitrogen (1.5%–4.0% for primary and 2.4%–5.0% for WAS) and phosphorous (0.17%–0.6% for primary and 0.6–2.3% for WAS) (Weemaes & Verstraete, 1999).

**Core Ideas**

- The study documents the range of concentrations of 40 per‐ and polyfluoroalkyl substances (PFAS) present in solids from 12 water resource recovery facilities in the United States, which may be used to inform development of regulatory policies.
- PFAS were measured in pre‐stabilized sludge and post‐stabilized biosolids using two different Environmental Protection Agency Methods.
- Measured PFAS were significantly different among the three laboratories.
- Higher 5:3 FTCA and PFOS concentrations were found in post‐stabilized biosolids than in pre‐stabilized sludges.

Primary sludge is captured by gravity, with or without coagulants or polymers, and has a higher energy content than WAS because microorganisms consume the energy content of sludge during secondary biological treatment (Weemaes & Verstraete, 1998). Pre‐stabilized sludge samples were comprised of a blend of primary sludge and secondary treated sludge/WAS. Table 1 provides pre‐stabilized sludge characteristics including blend ratios and thickening specifics for each WRRF. Post‐stabilized biosolids samples have undergone additional treatment, that is, anaerobic digestion or incineration (one site). The post‐stabilization treatment processes used at each WRRF are summarized in Table 2.

**TABLE 1:** Pre‐stabilized sludge information (Location A).

Site No.	Primary sludge thickening	Secondary sludge thickening	Primary:secondary sludge blend ratio	Blended sludge total solids (%)
1	Yes, gravity thickeners (GT)	Yes, centrifuge thickener	Varies	5.3
2	Yes, GT, primary and secondary sludge are co‐thickened		49%:51%	2.7
3	Yes, GT, primary and secondary sludge are co‐thickened		57%:43%	2.9
4	No thickening	Yes, gravity belt thickeners (GBT)	90%:10%	4.0
5	Yes, but blended along with secondary prior to thickening in GBT		55%:45%	6.2
6	No thickening	Yes, GBT	65%:35%	4.3
7	Yes, GT	Yes, GBT	55%:45%	4.6
8	Yes, GT	None	Varies	3.7
9	No thickening	Secondary is co‐thickened with primary using GBT.	Varies based on the time of the year	5.5
10	Yes, sludge concentration tanks	Yes, sludge concentration tanks	Varies	7.4
11	Yes, but blended along with secondary prior to thickening in rotary drum thickeners		53%:47%	1.6
12	Yes, ‐‐primary and secondary are combined and then thickened in air floatation tanks		64%:36%	5.6

**TABLE 2:** Post‐stabilized biosolids information (Location B).

Site No.	Stabilization method	Dewatering post stabilization	Disposal method	Type of biosolid
1	Anaerobic digestion	Centrifuge thickening	Land application	Class A
2	Anaerobic digestion	Centrifuge thickening	Landfill	–
3	Anaerobic digestion	Centrifuge thickening	Landfill	–
4	Anaerobic digestion	Belt filter press	Landfill	Class B
5	Anaerobic digestion	Screw press	Land application	Class B
6	Anaerobic digestion	Centrifuge thickening	Land application	Class A
7	Anaerobic digestion	Centrifuge thickening	Land application	Class B
8	Incineration	–	Landfill	–
9	Anaerobic digestion	Centrifuge thickening	Land application	Class B
10	Anaerobic digestion	Lagoons	Land application	Class A/B
11	Anaerobic digestion	Centrifuge thickening	Landfill	–
12	Anaerobic digestion	Centrifuge thickening	Land application	Class A EQ^a^

^a^ Class A EQ, exceptional quality (Class A) biosolids.

### 2.1 Sample collection

Sampling at the 12 WRRFs was conducted between September 19 and October 31, 2022 (Figures 1 and 2). Samples were collected by WRRF personnel according to the PFAS sampling protocol using a PFAS‐free sampling kit as described in Figures S1–S4. The sampling protocol included a sampling checklist addressing appropriate field clothing, personal protection equipment, field equipment, equipment decontamination, and food packaging contamination consideration (Michigan Deptartment EGLE, 2020). Samples were shipped overnight, on ice, to a centralized facility (Brown and Caldwell Treatability Laboratory). Samples were homogenized and split into three PFAS‐free sample bottles; one set was shipped to the commercial laboratory, while the other two sets were frozen (below −20°C) and shipped to university laboratories after all WRRF samples were collected. Upon receipt of samples, the commercial laboratory analyzed samples within 14 days, and the university laboratories froze samples until analyses were conducted. Henceforth, to maintain anonymity, laboratories are referenced as Lab 1, Lab 2, and Lab 3.

### 2.2 Sample preparation, extraction, detection, and quantification

At the time of sample analyses, PFAS were analyzed using EPA Method SW846‐3500C/537.1 (US EPA, 2022) and Draft Method 1633 (US EPA, 2023b), which has been finalized since this work was conducted. Table 3 summarizes general sample preparation, extraction, detection, and quantification performed by each of the participating laboratories. Lab 1 analyzed samples according to Method SW846‐3500C/537.1, and Lab 2 and Lab 3 followed the Draft Method 1633, as published. Sample analysis dates are summarized in Table S1.

**TABLE 3:** Sample preparation, extraction, detection, and quantification at the three laboratories.

Step	Lab 1	Lab 2	Lab 3
1	A well homogenized sub‐sample (∼1 g) was fortified with isotopically labeled Extracted Internal Standard (EIS).	A known mass (∼5 g) of as received sample was spiked with 0.1 mL of EIS dilution solution containing 24 mass labeled per‐ and polyfluoroalkyl substances (PFAS) isotopes and equilibrated for at least half an hour.	Approximately 0.4 g dry weight of sample was spiked with 20 µL of EIS standard dilution solution containing 24 mass labeled PFAS isotopes, and equilibrated for at least half an hour.
2	Extraction with solvent was performed using ultrasonic extraction for 1 h. Supernatant collected after centrifugation.	Extraction (three times) with 0.3% methanolic ammonium hydroxide was performed and supernatant collected after centrifuging.	Extraction (three times, 15, 10, and 5 mL) with 0.3% methanolic ammonium hydroxide was performed and supernatant collected after centrifuging.
3	The extracts are vortexed and centrifuged. A 2.0 mL portion of supernatant was transferred and concentrated with a low flow of nitrogen in a heated water bath and then reconstituted to 1.0 mL with methanol.	Volume of the supernatant was reduced based on the solid content of the sample using nitrogen gas flow.	Volume of the supernatant was reduced based on the solid content of the sample using nitrogen gas flow.
4	–	Reagent water was added, and pH was adjusted to 6.5 ± 0.5.	Reagent water was added, and pH was adjusted to 6.5 ± 0.5
5	Isotopically labeled non‐extracted internal standards (NISs) are added to the sample extract.	The sample was loaded onto a weak anion exchange (WAX) solid‐phase extraction (SPE) cartridge half‐filled with silanized glass wool after washing to remove potential interferences. The samples were eluted using 1% methanolic ammonium hydroxide into a 10‐mL centrifuge tube containing seven NIS PFAS.	The sample was loaded onto a WAX‐SPE cartridge half‐filled with silanized glass wool after washing to remove potential interferences. The samples were eluted using 1% methanolic ammonium hydroxide into a 15‐mL centrifuge tube containing seven NIS PFAS.
6	Extracts were analyzed by liquid chromatography/tandem mass spectrometry (LC‐MS/MS) operated at negative electrospray ionization mode for detection and quantification of the analytes. Quantitative analysis is performed using isotope dilution. Secondary transitions are used for confirmation when available.	Extracts were analyzed using ultrahigh LC‐MS/MS in the multiple reaction monitoring (MRM) mode on a Waters Xevo triple quadrupole mass spectrometer (TQMS) (Waters). The identified PFAS were confirmed with two pairs of ion transitions.	Extracts were analyzed using Shimadzu Prominence High Performance LC coupled to a Sciex 4500 Q‐TrapTQMS in the scheduled MRM mode. The MRM transitions have been optimized for each compound for the Sciex Q‐Trap TQMS, and the identified PFAS were confirmed with two pairs of ion transitions.

Because Method 537.1 was developed specifically for potable water, application of this method to biosolids requires some modifications. EPA Method 537.1 is a drinking water method and has only been evaluated by the EPA for use in analyzing drinking water samples. This drinking water method relies on solid‐phase extraction (SPE) procedures to separate the PFAS analytes from the sample matrix and although it works well for wastewater analyses, the applications of SPE as described in EPA Method 537.1 require adjustments to handle the suspended solids in a typical wastewater discharge. Because the drinking water method does not contain many of the mandatory cleanup steps in the Draft EPA Method 1633 that are needed to analyze many wastewater samples, the commercial laboratory used in this study has used SW846‐3550C to clean up the samples prior to extraction and analyzed by EPA Method 537.1. The term “modified” means that the analytical procedure has been changed from what is allowed in the published method; given that drinking water methods do not allow for modification, the label “modified” is misleading. In the case of this project, the commercial laboratory used EPA Method 537.1 “modified” to provide sample clean up using EPA Method SW846‐3550C. In this method, biosolids samples were fortified with isotopically labeled extraction standards and were prepared using ultrasonic extraction according to EPA Method SW846‐3550C. In this method, EPA acknowledges there is no single extraction solvent that is universally applicable to all analyte groups and that the analyst must demonstrate adequate performance for the analytes of interest at the levels of interest. Thus, samples analyzed by the commercial laboratory were extracted using a proprietary solvent system that gives optimum, reproducible recovery of the analytes of interest from the sample matrix, at the concentrations of interest in this study. The commercial laboratory has demonstrated proficiencies for this extraction protocol as described in EPA Method SW846‐3550C, using a clean reference matrix, and implemented analytical procedures to develop performance criteria for the performance demonstration, as well as for matrix spike and laboratory control sample results. Following ultrasonication, sample extracts were vortexed and centrifuged; a 2.0 mL portion of supernatant was transferred and concentrated with nitrogen in a heated water bath and reconstituted to 1.0 mL with methanol. Isotopically labeled internal standards were added to the sample extract which was analyzed by liquid chromatography‐tandem mass spectrometry (LC‐MS/MS). Quantitative analyses of sample extracts were performed using isotope dilution according to modified EPA Method 537.1. Because this method is written specifically for the analysis of drinking water samples, augmentation to the method was made to accommodate the preparation of biosolids samples which are not addressed in EPA 537.1, as follows:

1. Where commercially available, a labeled isotopic analog—extraction standards—was spiked into samples prior to extraction. For those compounds, an isotope dilution calibration model is used. Where labeled isotopes are not available, an internal standard calibration model using the extraction standards is used.
2. Prior to instrumental analysis, separate but similar isotopic analogs were added to the sample extracts. Using an internal standard calibration model, these injection standards were used to calculate recoveries of the extraction standards.
3. Field reagent blanks were not processed as listed in EPA 537.1, Section 8.3.
4. Spike concentrations were not rotated between low, medium, and high levels.

The QC program for these analyses included minimum detection limit (MDL) studies, reporting limits (RLs), initial demonstration of capability, and ongoing QC requirements that must be met, according to the referenced methods, when preparing and analyzing samples. The QC parameters, their required frequencies, and the performance criteria that meet EPA data quality objectives are provided in tables 12 and 13 of EPA Method 537.1. These QC requirements are considered the minimum acceptable QC criteria. As part of the QC program, analysts must consider analytical method interferences. Bangma et al. (2023) documented such interferences for perfluoropentanoic acid and perfluorobutanoic acid using low‐resolution MS/MS calling into question quantitative results for these two PFAS.

For both methodologies, EPA SW846‐3500C/537.1 and Draft EPA Method 1633, PFAS standards used for matrix fortification, native standards, and isotope‐labeled PFAS analogues used by the three laboratories were all purchased from Wellington Laboratories Inc. PFAS compounds are evaluated, and isotopes used by the three laboratories are summarized in Tables S2 and S3, respectively.

## 3 RESULTS AND DISCUSSION

### 3.1 Data quality standards

Analytical data were assessed for data quality using (1) quality assurance (QA)/QC data provided by each analytical laboratory, (2) Brown and Caldwell data verification and validation guidelines for reporting general chemistry, (3) guidance documents from US EPA and the Interstate Technology Regulatory Council, (4) information from EPA Methods SW846‐3550C and 537.1 and, though not yet promulgated, guidance from Draft EPA Method 1633, and (5) the expertise of the research team. A four‐step assessment process was applied through the following stages (IRTC, 2022): (a) verification of data, (b) validation of data, (c) data quality, and (d) data usability. Data usability was determined by the project team after verification, validation, or any other data quality review was complete, and the overall quality of the collected data is known. Simply, a data usability assessment allows determination of whether the quality of analytical data is fit for its intended use. This approach was recently applied for data evaluation by Winchell et al. (2024). Definitions for MDL and RL are important to define in context of this evaluation:

1. *The MDL must be established by each laboratory for all target analytes using procedures in 40 CFR Part 136, Appendix B. The MDL is the minimum measured concentration of a substance that can be reported with 99% confidence that the measured analyte concentration is distinguishable from method blank results* (US EPA, 2023b).
2. *The reporting level (RL), unless specified otherwise by a regulatory authority or discharge permit, for analytes that meet the identification criteria is reported down to the concentration of the minimum level of quantification  established by the laboratory through calibration of the instrument, that is, the lowest calibration standard concentration. Notably, US EPA considers the terms “reporting limit,” “quantitation limit,” “limit of quantitation,” and “minimum level” to be synonymous* (US EPA, 2023c).

Laboratories used these definitions for data qualifiers published with results, according to the relevant method; a description of qualifiers is summarized in Table 4. Considering the complexity of sample matrices in this study, a rigorous data usability review was conducted; thus, along with data qualifiers, the data usability assessment rule is also described in Table 4.

**TABLE 4:** Qualifiers, descriptions, and data usability assessment rules.

Qualifier	Description	Rules for data usability
U	Undetected/not detected	Data are non‐detected and "<RL" is used and not considered for numerical analysis.
*5+	Isotope dilution analyte is outside acceptance limits, high bias.	Data cannot be used for analyses or evaluations.
*5−	Isotope dilution analyte is outside acceptance limits, low bias.	Data cannot be used for analyses or evaluations.
J	Result is less than the RL but greater than or equal to the MDL and the concentration is an approximate value.	Data cannot be used for analyses or evaluations.
F1	MS and/or MSD recovery exceeds control limits (matrix spike/matrix spike duplicate [MS/MSD]).	Data cannot be used for analyses or evaluations.
*‐	Laboratory control sample (LCS) and/or laboratory control sample duplicate (LCSD) is outside acceptance limits, low biased.	Data cannot be used for analyses or evaluations.
*+	LCS and/or LCSD is outside acceptance limits, high biased.	Data cannot be used for analyses or evaluations.
cn	Refer to case narrative for further detail.	Specific evaluation of data usability will depend on the case narrative explanation and professional judgement
I	Value is estimated maximum possible concentration.	Data cannot be used for analyses or evaluations.
^2	Calibration blank is outside acceptance limits.	Data cannot be used for analyses or evaluations.
F2	MS/MSD RPD exceeds control limits.	Data cannot be used for analyses or evaluations.

### 3.2 PFAS in sludge and biosolids samples

Of the PFAS compounds in Table S2, 15 compounds were detected in pre‐stabilized sludge (Location A) and post‐stabilized biosolids (Location B) from all laboratories. Notably, molecular weights of the analyzed PFAS compounds range from 300.1 to 614.1 g/mole and because of the substantial difference in molecular weights and the fact that analytical results are reported on a dry mass basis, comparisons are only made among sites and laboratories for individual compounds. Any comparisons using summations of multiple compounds would require conversion of mass to molar concentrations. Results, qualifiers, RLs, and MDLs are tabulated in Table S4 on a dry mass basis.

There were differences in the number of PFAS compounds detected among the laboratories. For pre‐stabilized sludge, four compounds were detected by Lab 1, two by Lab 2, and 12 by Lab 3. For post‐stabilized biosolids, seven compounds were detected by Lab 1, six by Lab 2, and 13 by Lab 3. The PFAS compounds detected above the RL by the three laboratories are summarized in Table S5.

**FIGURE 3:**
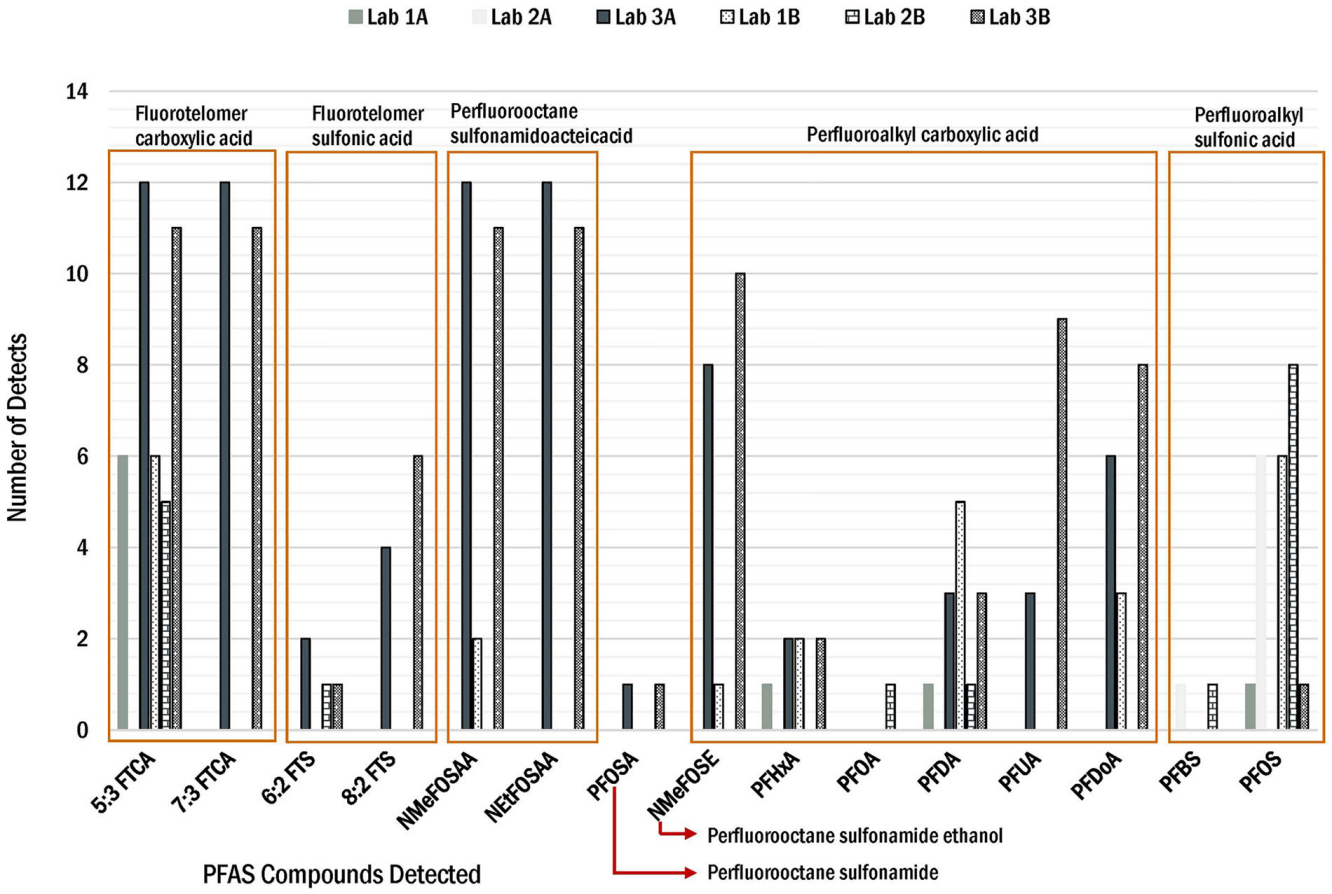
Class of per‐ and polyfluoroalkyl substances (PFAS) compound and number of compounds detected by three laboratories for Locations A and B.

In EPA Method 1633, PFAS are grouped into nine categories (Table S2). The number of samples measured above the RL for pre‐stabilized sludge and post‐stabilized biosolids is shown in Figure 3. Results showed more detections for fluorotelomer carboxylic acids, perfluorooctanesulfonamido acetic acids, and perfluoroalkyl carboxylic acids than for other PFAS groups.

**FIGURE 4:**
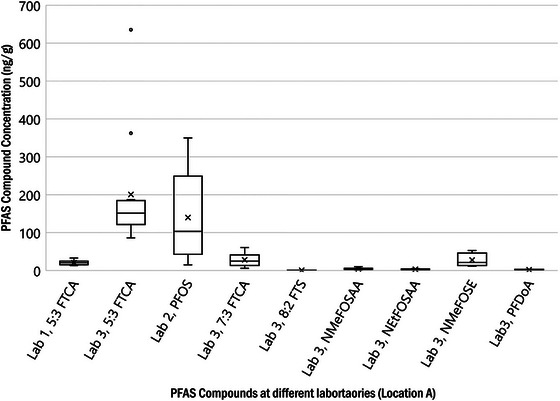
Per‐ and polyfluoroalkyl substances (PFAS) concentration ranges reported for pre‐stabilized sludge (Location A). NMeFOSE, perfluorooctane sulfonamide ethanol.

**FIGURE 5:**
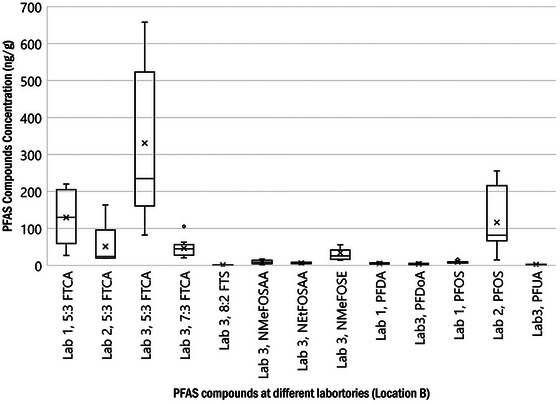
Per‐ and polyfluoroalkyl substances (PFAS) concentration ranges for post‐stabilized biosolids (Location B). NMeFOSE, perfluorooctane sulfonamide ethanol.

The reported PFAS compound concentration ranges, in nanogram per gram, are presented in Figure 4 (pre‐stabilized sludge) and Figure 5 (post‐stabilized biosolids). These figures summarize concentrations reported by all three laboratories for compounds with more than three detections per compound.

US EPA is anticipated to complete risk assessments for PFOA and PFOS by the end of 2024, which would serve as the basis for potential regulation of these compounds in biosolids if potential risks are identified (US EPA, 2021). PFOS was most frequently detected by both Lab 1 and Lab 2, but not reported by Lab 3 due to the high background of PFOS in their LC‐MS/MS. PFOA was detected by one laboratory, at one site only.

PFOS in post‐stabilized biosolids (Location B) were consistent with previous studies, where concentrations were within 80–160 ng/g, and PFOA was found near the detection limit (Higgins et al., 2005; Schultz et al., 2006). Interestingly, both Lab 1 and Lab 2 reported more PFOS detections in post‐stabilized biosolids than in pre‐stabilized sludges. At least one other study has reported this increase in PFAS concentrations through stabilization (Li et al., 2021).

Other compounds of interest with respect to data observations in Figures 4 and 5 are the fluorotelomers, specifically 5:3 FTCA (fluorotelomer carboxylic acid), 7:3 FTCA, 6:2 FTS (fluorotelomer sulfonic acid), and 8:2 FTS. Because 5:3 FTCA was detected by all three laboratories, results for all 12 WRRFs are tabulated in Table 5.

**TABLE 5:** 5:3 FTCA (fluorotelomer carboxylic acid) concentrations for all 12 water resource recovery facility (WRRF) sites in, both pre‐stabilized sludge and post‐stabilized biosolids.

WRRF site number	Pre‐stabilized (ng/g)			Post‐stabilized (ng/g)		
	Lab 1	Lab 2	Lab 3	Lab 1	Lab 2	Lab 3
1	22	<34.2	146	220	<75.8	616
2	13	<57.3	104	200	<103.2	523
3	16	<67.6	177	160	<102.8	304
4	*‐ cn	<6	187	*‐ cn	19	235
5	U*‐ cn	<37	117	70	24	193
6	33	<40.8	164	100	28	508
7	21	<34.9	86	27	<7.6	161
8	20	<67.5	362	<0.6	<1.5	<1.6
9	*‐ cn	<27.6	156	*‐ cn	<6	82
10	*‐ cn	<26	137	*‐ cn	<7.5	141
11	*‐ cn	<206.1	635	*‐ cn	163.5	658
12	*‐ cn	<31.6	134	*‐ cn	20	219

*Note*: The less than values in the table report the reporting limit as per the decision made during data quality and usability steps.

Abbreviation: cn, case narrative.

Examination of 5:3 FTCA results demonstrate that its concentrations in post‐stabilized biosolids were higher than in pre‐stabilized sludges—even when solids reduction through stabilization has been considered. PFAS concentrations in the post‐stabilized biosolids were 1–10 order of magnitude higher than in pre‐stabilized sludges. This is consistent with other research where 5:3 FTCA concentrations increased following anaerobic digestion where researchers identified 5:3 FTCA (and 6:2 FTCA) as products of anaerobic 6:2 fluorotelomer alcohol degradation (Zhang et al., 2013).

**FIGURE 6:**
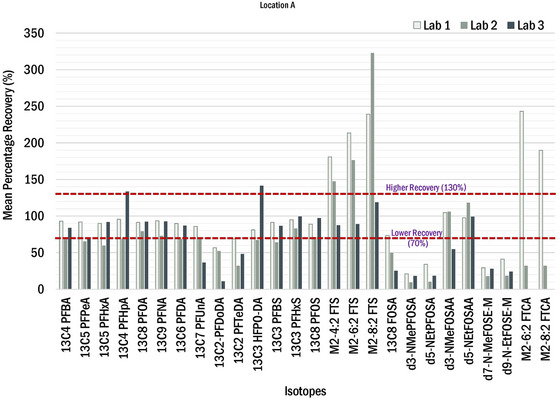
Mean percentage recoveries of per‐ and polyfluoroalkyl substances (PFAS) extracted internal standard from the pre‐stabilized sludges from all the three laboratories. (Lab 3 followed the EPA method 1633 in which M‐FTCAs are not included in the extract internal standard list. Therefore, these classes of chemicals are not included.).

**FIGURE 7:**
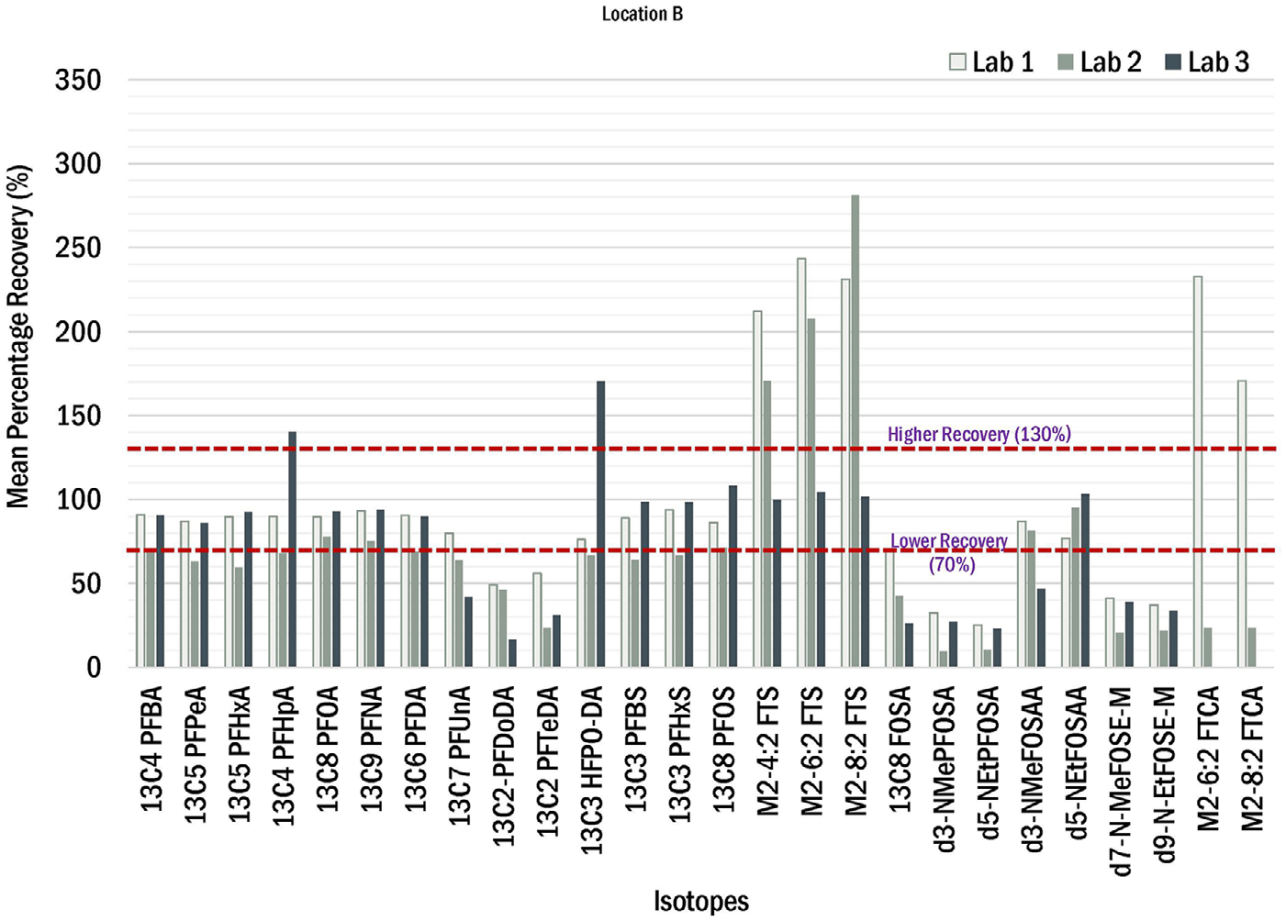
Mean percentage recoveries of per‐ and polyfluoroalkyl substances (PFAS) extracted internal standards from post‐stabilized biosolids from all the three laboratories. (Lab 3 followed the EPA method 1633 in which M‐FTCAs are not included in the extract internal standard list. Therefore, these classes of chemicals are not included.).

To confirm increases in PFOS and 5:3 FTCA from pre‐stabilized sludge to post‐stabilized biosolids were not an analytical anomaly, relevant isotope dilution analyte (IDA) recoveries were examined. As previously described, the US EPA methods used for this investigation included isotope dilution, where carbon‐13 (^13^C)‐labeled analogs, oxygen‐18 (^18^O)‐labeled analogs, or deuterated analogs of the compounds of interest were spiked into the samples at the time of extraction. This allows correction for analytical biases encountered in chemically complex environmental samples because the isotopically labeled compounds are chemically like the compounds of concern and are impacted by the same sample‐related interferences as compounds of concern. The mean percentage recoveries of IDA among the three laboratories for pre‐stabilized sludges and post‐stabilized biosolids are shown in Figures 6 and 7, respectively. The red line defines the lower (70%) and higher (130%) recovery bound as described in the Draft EPA Method 1633. The IDAs for PFOS and 5:3FTCA are ^13^C_8_‐PFOS and ^13^C_5_‐PFHxA, respectively. The extraction recovery for these IDAs were within the acceptable range, except for Lab 2, which had low recoveries for ^13^C_5_‐PFHxA.

While there is currently an analytical method for measurement of PFAS in wastewater sludge and biosolids was finalized shortly after this work was completed, recent research has used various methods to measure PFAS. EPA Methods SW846‐3550C/537.1 and Draft EPA Methods 1633 have been used. The multi‐laboratory validation study report supporting the final method summarizes the results for solid matrices and landfill leachate matrices (US EPA, 2023b).

Although EPA QC criteria were generally met by each laboratory, data from the split samples showed poor agreement among laboratories. While this low agreement could be attributed to poor homogenization of samples, other studies have found consistency among split biosolids samples. Winchell et al. also showed that PFAS concentrations in biosolids were consistent over multiple sampling events throughout an operating day when working with the same commercial laboratory using EPA Method SW846‐3550C/537.1 (Winchell et al., 2024).

### 3.3 Statistical analysis

The Wilcoxon rank sum test evaluation was conducted to evaluate if measured concentrations of PFAS compounds were significantly different among the laboratories (Frey, 2023). For pre‐stabilized sludges, Lab 2 reported all non‐detects; therefore, only a comparison between Lab 1 and Lab 3 was made. For post‐stabilized biosolids, a comparison among all three laboratories was performed. The *α* value was chosen to be 0.1 because the population size varied from 5 to 15. The statistics summary analysis is presented in Table S6 and shows that for both pre‐ and post‐stabilized samples, there were significant differences between Lab 1 and Lab 3. No significant differences were observed between Lab 1 and Lab 2, and Lab 2 and Lab 3 in post‐stabilized biosolids. This could be attributed to the smaller number of non‐detects observed in the Lab 2 data. Tables S7 and S8 provide the Lab 2 limit of quantification (LOQ) and Lab 3 limit of detection and LOQ, respectively.

## 4 SUMMARY AND CONCLUSIONS

The widespread occurrence of PFAS and emerging regulations necessitates a better understanding of their concentrations in biosolids. The US EPA has initiated efforts to fill this knowledge gap and has performed analyses of PFAS in samples collected through National Sewage Sludge Survey (NSSS). In the first (US EPA, 2007), samples were acquired and analyzed for PFAS to establish baseline concentrations for a national inventory of these chemicals in treated municipal biosolids. The original survey of 13 PFAS in US WRRF biosolids composites from 32 States and the District of Columbia showed that PFOS concentrations were 403 ± 127 ng/g as the most abundant PFAS, followed by PFOA at 34 ± 22 ng/g (Venkatesan & Halden, 2013). Considering that the primary US manufacturer of PFOS voluntarily phased out production of PFOS in 2002, and eight other companies voluntarily agreed to phase out production of PFOS and PFOA‐related chemicals by 2015, it is not surprising that PFOS and PFOA concentrations were less than those reported for the 2001 NSSS in this surveillance study.

While this study is the first to report ranges of 40 PFAS concentrations in pre‐stabilized sludges and post‐stabilized biosolids, findings from this study point to a critically important consideration in evaluating QC data along with reported analytical results. If PFAS regulations emerge, laboratory certification may become an issue. This is not surprising, considering the low levels of PFAS compounds in complicated matrices such as wastewater sludge. Additionally, manual extraction and analysis protocols conducted by different analytical staff could lead to some analytical variation, which may be reflected in QC samples. Certified samples are needed to validate the methods and operations of each laboratory in the measurement of trace‐level PFAS. In context of analyzing complex matrices and interpreting the data, it is essential to leverage appropriate QA/QC tools to assure that evaluations related to risk assessment, regulation development, and compliance monitoring are accurate and precise. Inadequate QC protocols, specifically including data usability reviews, could result in inaccurate conclusions related to actual human health or ecological risks and multiply the costs of treatment or remediation to achieve regulatory compliance.

## AUTHOR CONTRIBUTIONS

**Shubhashini Oza**: Conceptualization; data curation; formal analysis; investigation; methodology; validation; writing—original draft. **Katherine Y. Bell**: Conceptualization; funding acquisition; supervision; writing—review and editing. **Zhiliang Xu**: Investigation; methodology; validation; writing—review and editing. **Yifei Wang**: Investigation; methodology; validation; writing—review and editing. **Martha J. M. Wells**: Supervision; writing—review and editing. **John W. Norton. Jr**.: Funding acquisition; supervision; writing—review and editing. **Lloyd J. Winchell**: Writing—review and editing. **Qingguo Huang**: Conceptualization; funding acquisition; resources; supervision; writing—review and editing. **Hui Li**: Conceptualization; funding acquisition; resources; supervision; writing—review and editing.

## CONFLICT OF INTEREST STATEMENT

Martha J. M. Wells is a chemical consultant to Brown and Caldwell and served in that role while assisting with preparation of this manuscript. John Norton is the Director of Energy, Research, and Innovation at Great Lakes Water Authority who provided partial funding for this work along with US EPA funding.

## Supporting information

Figure S1: Page one of Sampling Guideline Document shared with 12 WRRF.
Figure S2: Page two of Sampling Guideline shared with 12 WRRF.
Figure S3: Page three of Sampling Guideline shared with 12 WRRF.
Figure S4: Page four of Sampling Guideline shared with 12 WRRF.
Table S1: Sample analysis dates.
Table S2: Summary of the 40 PFAS compounds evaluated by the three laboratories.
Table S3: Summary of PFAS Isotopes used by the laboratories.
Table S4: Measured concentrations for all 12 WRRF from three Laboratories (ng/g dry basis).
TABLE S5: PFAS compounds detected in pre‐stabilized and post‐stabilized sludge sample by the three laboratories.
Table S6: Wilcoxon rank sum test evaluation.
Table S7: Lab 2 Limit of quantification (LOQ).
Table S8: Lab 3 Limit of detection and LOQ.
