# Supplementary material for: Surveillance of PFAS in sludge and biosolids at 12 water resource recovery facilities
Source: J Environ Qual. 2024 Jul 14;54(1):6–19. doi: 10.1002/jeq2.20595 (PMC11718139; doi:10.1002/jeq2.20595)
Supplement: Supplementary file 1 — Figure S1: Page one of Sampling Guideline Document shared with 12 WRRF. Figure S2: Page two of Sampling Guideline shared with 12 WRRF. Figure S3: Page three of Sampling Guideline shared with 12 WRRF. Figure S4: Page four of Sampling Guideline shared with 12 WRRF. Table S1: Sample analysis dates. Table S2: Summary of the 40 PFAS compounds evaluated by the three laboratories. Table S3: Summary of PFAS Isotopes used by the laboratories. Table S4: Measured concentrations for all 12 WRRF from three Laboratories (ng/g dry basis). TABLE S5: PFAS compounds detected in pre‐stabilized and post‐stabilized sludge sample by the three laboratories. Table S6: Wilcoxon rank sum test evaluation. Table S7: Lab 2 Limit of quantification (LOQ). Table S8: Lab 3 Limit of detection and LOQ. [file JEQ2-54-6-s001.docx]

**SUPPLEMENTAL MATERIAL**

**Surveillance of PFAS in Sludge and Biosolids at 12 Water Resource Recovery Facilities**

Shubhashini Oza^1*^, Katherine Y. Bell^1^, Zhiliang Xu^2^, Yifei Wang^3^, Martha J.M. Wells^4^, John W. Norton, Jr.^5^, Lloyd J. Winchell^1^, Qingguo Huang^3^, Hui Li^2*^

^1^ Brown and Caldwell, Charlotte, NC, Nashville, TN, and Saint Paul, MN, USA.

^2^ Department of Plant, Soil and Microbial Sciences, Michigan State University, East Lansing, MI, USA.

^3^ Department of Crop and Soil Science, University of Georgia, Griffin, GA, USA.

^4^ EnviroChem Services, Cookeville, TN, USA.

^5^ Great Lakes Water Authority, Detroit, MI, USA.

^*^ Correspondence: Hui Li, [lihui@msu.edu](mailto:lihui@msu.edu) and Shubhashini Oza, [soza@brwncald.com](mailto:soza@brwncald.com)


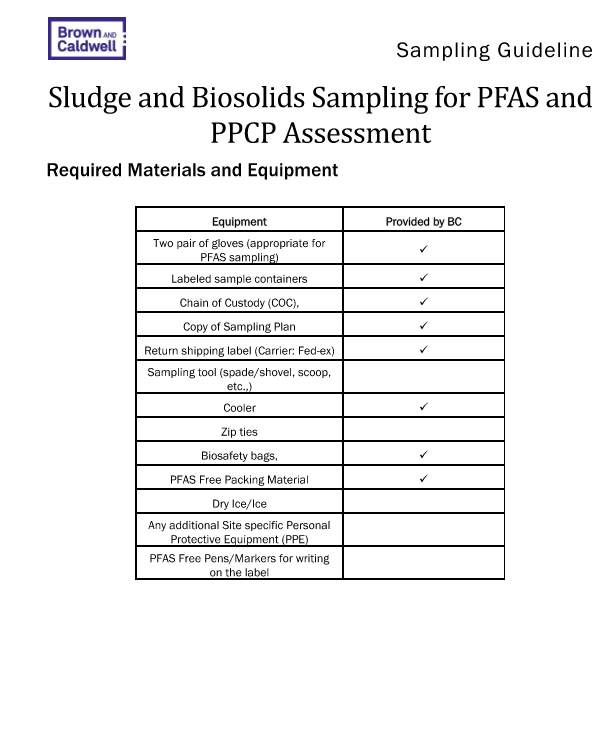


**Figure S1**: Page one of Sampling Guideline Document shared with twelve WRRF.


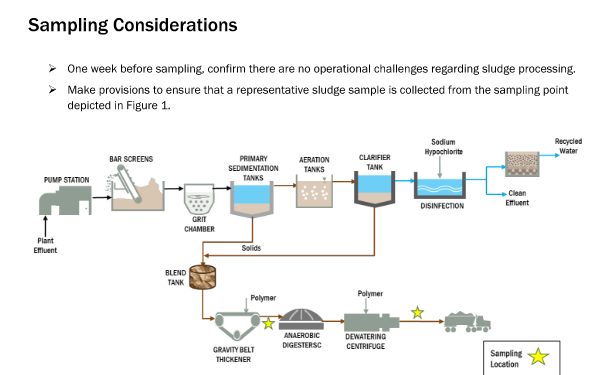


**Figure S2**: Page two of Sampling Guideline shared with twelve WRRF.


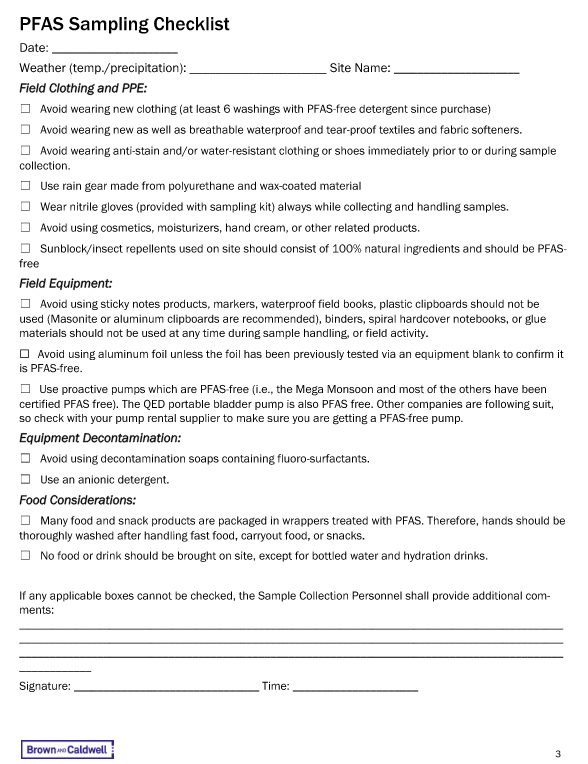


**Figure S3**: Page three of Sampling Guideline shared with twelve WRRF.


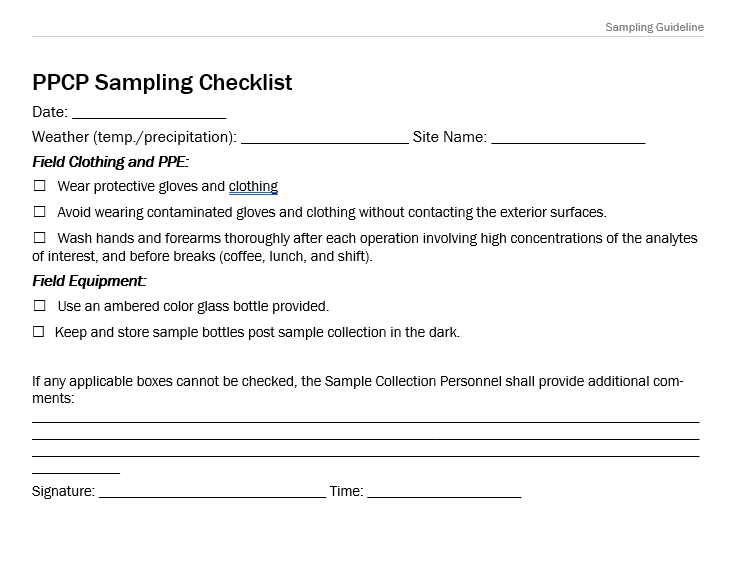


**Figure S4**: Page four of Sampling Guideline shared with twelve WRRF.

**Table S1**: Sample Analysis Dates

| **Item** | **Lab 1** | **Lab 2** | **Lab 3** |
| --- | --- | --- | --- |
| 1A, 1B | 10/7/2022, 10/13/2022 | 10/10/2022 | 3/30/2023 |
| 2A, 2B | 10/13/2022, 10/13/2022 | 10/12/2022 | 3/30/2023 |
| 3A, 3B | 10/13/2022, 10/13/2022 | 10/12/2022 | 3/30/2023 |
| 4A, 4B | 10/28/2022, 10/28/2022 | 10/13/2022 | 3/30/2023 |
| 5A, 5B | 11/9/2022, 11/9/2022 | 10/14/2022 | 3/30/2023 |
| 6A, 6B | 11/9/2022, 11/9/2022 | 10/17/2022 | 3/30/2023 |
| 7A, 7B | 11/9/2022, 11/9/2022 | 10/18/2022 | 3/30/2023 |
| 8A, 8B | 11/11/2022, 11/14/2022, 11/23/2022 | 11/7/2022-11/9/2022 | 3/30/2023 |
| 9A, 9B | 11/21/2022, 11/21/2022 | 11/11/2022 | 3/30/2023 |
| 10A, 10B | 11/21/2022, 11/21/2022 | 11/17/2022 | 3/30/2023 |
| 11A, 11B | 11/23/2022, 11/23/2022 | 11/18/2022 | 3/30/2023 |
| 14A, 14B | 12/6/2022, 12/10/2022 | 11/25/2022 | 3/30/2023 |

**Table S2**: Summary of the forty PFAS compounds evaluated by the three laboratories.

| **Analyte** | **Acronym** | **Category** |
| --- | --- | --- |
| 3-Perfluoropropylpropanoic acid | 3:3 FTCA | Fluorotelomer carboxylic acid |
| 3-Perfluoropenthylpropanoic acid | 5:3 FTCA | Fluorotelomer carboxylic acid |
| 3-Perfluoroheptylpropanoic acid | 7:3 FTCA | Fluorotelomer carboxylic acid |
| 1H,1H,2H,2H-Perfluorohexane sulfonic acid | 4:2 FTS | Fluorotelomer sulfonic acid |
| 1H,1H, 2H, 2H-Perfluorooctane sulfonic acid | 6:2 FTS | Fluorotelomer sulfonic acid |
| 1H,1H, 2H, 2H-Perfluorodecane sulfonic acid | 8:2 FTS | Fluorotelomer sulfonic acid |
| Perfluoro-3-methoxypropanoic acid | PFMPA | Per- and polyfluoroether carboxylic acid |
| Perfluoro-4-methoxybutanoic acid | PFMBA | Per- and polyfluoroether carboxylic acid |
| Perfluoro-3,6-dioxaheptanoic acid | NFDHA | Per- and polyfluoroether carboxylic acid |
| Hexafluoropropylene oxide dimer acid | HFPO-DA | Per- and polyfluoroether carboxylic acid |
| 4,8-Dioxa-3H-perfluorononanoic acid | DONA | Per- and polyfluoroether carboxylic acid |
| N-methylperfluorooctane sulfonamido acetic acid | NMeFOSAA | Perfluorooctane sulfonamido acetic acid |
| N-ethylperfluorooctane sulfonamido acetic acid | NEtFOSAA | Perfluorooctane sulfonamido acetic acid |
| Perfluorooctane sulfonamide | PFOSA | Perfluorooctane sulfonamide |
| N-methylperfluorooctane sulfonamide | NMeFOSA | Perfluorooctane sulfonamide |
| N-ethylperfluorooctane sulfonamide | NEtFOSA | Perfluorooctane sulfonamide |
| N-methylperfluorooctane sulfonamidoethanol | NMeFOSE | Perfluorooctane sulfonamide ethanol |
| N-ethylperfluorooctane sulfonamidoethanol | NEtFOSE | Perfluorooctane sulfonamide ethanol |
| Perfluorobutanoic acid | PFBA | Perfluoroalkyl carboxylic acid |
| Perfluoropentanoic acid | PFPeA | Perfluoroalkyl carboxylic acid |
| Perfluorohexanoic acid | PFHxA | Perfluoroalkyl carboxylic acid |
| Perfluoroheptanoic acid | PFHpA | Perfluoroalkyl carboxylic acid |
| Perfluorooctanoic acid | PFOA | Perfluoroalkyl carboxylic acid |
| Perfluorononanoic acid | PFNA | Perfluoroalkyl carboxylic acid |
| Perfluorodecanoic acid | PFDA | Perfluoroalkyl carboxylic acid |
| Perfluoroundecanoic acid | PFUA | Perfluoroalkyl carboxylic acid |
| Perfluorododecanoic acid | PFDoA | Perfluoroalkyl carboxylic acid |
| Perfluorotridecanoic acid | PFTrDA | Perfluoroalkyl carboxylic acid |
| Perfluorotetradecanoic acid | PFTeA | Perfluoroalkyl carboxylic acid |
| Perfluorobutanesulfonic acid | PFBS | Perfluoroalkyl sulfonic acid |
| Perfluoropentanesulfonic acid | PFPS | Perfluoroalkyl sulfonic acid |
| Perfluorohexanesulfonic acid | PFHxS | Perfluoroalkyl sulfonic acid |
| Perfluoroheptanesulfonic acid | PFHpS | Perfluoroalkyl sulfonic acid |
| Perfluorooctanesulfonic acid | PFOS | Perfluoroalkyl sulfonic acid |
| Perfluoro nonanesulfonic acid | PFNS | Perfluoroalkyl sulfonic acid |
| Perfluorodecanesulfonic acid | PFDS | Perfluoroalkyl sulfonic acid |
| Perfluorododecanesulfonic acid | PFDoS | Perfluoroalkyl sulfonic acid |
| Perfluoro(2-ethoxyethane) sulfonic acid | PFEESA/PES | Ether sulfonic acid |
| 9-Chlorohexadecafluoro-3-oxanonane-1-sulfonic acid | 9Cl-PF3ONS | Ether sulfonic acid |
| 11-Chloroeicosafluoro-3-oxaundecane-1-sulfonic acid | 11Cl-PF3OUdS | Ether sulfonic acid |

**Table S3**. Summary of PFAS Isotopes used by the laboratories.

| **Isotopes** | **Acronym** | **Lab 1** | **Lab 2** | **Lab 3** |
| --- | --- | --- | --- | --- |
| Perfluoro-n-[2,3,4-13C3]butanoic acid | 13C4 PFBA | Yes | Yes | Yes |
| Perfluoro-n-[13C5]pentanoic acid | 13C5 PFPeA | Yes | Yes | Yes |
| Perfluoro-n-[1,2,3,4,6-13C5]hexanoic acid | 13C5 PFHxA | Yes | Yes | Yes |
| Perfluoro-n-[1,2,3,4-13C4]heptanoic acid | 13C4 PFHpA | Yes | Yes | Yes |
| Perfluoro-n-[13C8]octanoic acid | 13C8 PFOA | Yes | Yes | Yes |
| Perfluoro-n-[1,2,3,4,5-13C5]nonanoic acid | 13C9 PFNA | Yes | Yes | Yes |
| Perfluoro-n-[1,2,3,4,5,6-13C6]decanoic acid | 13C6 PFDA | Yes | Yes | Yes |
| Perfluoro-n-[1,2,3,4,5,6,7-13C7]undecanoic acid | 13C7 PFUnA | Yes | Yes | Yes |
| Perfluoro-n-[1,2-13C2]dodecanoic acid | 13C2-PFDoDA | Yes | Yes | Yes |
| Perfluoro-n-[1,2-13C2]tetradecanoic acid | 13C2 PFTeDA | Yes | Yes | Yes |
| Tetrafluoro-2-heptafluoropropoxy-13C3-propanoic acid | 13C3 HFPO-DA | Yes | Yes | Yes |
| Perfluoro-n-[4,1-13C1]butanesulfonic acid | 13C3 PFBS | Yes | Yes | Yes |
| Perfluoro-1-[1,2,3-13C3]hexanesulfonic acid | 13C3 PFHxS | Yes | Yes | Yes |
| Perfluoro-n-[13C8]octanesulfonic acid | 13C8 PFOS | Yes | Yes | Yes |
| 1H,1H,2H,2H-Perfluoro-1-[1,2-13C2]hexane sulfonic acid | M2-4:2 FTS | Yes | Yes | Yes |
| 1H,1H,2H,2H-Perfluoro-1-[1,2-13C2]octane sulfonic acid | M2-6:2 FTS | Yes | Yes | Yes |
| 1H,1H,2H,2H-Perfluoro-1-[1,2-13C2]decane sulfonic acid | M2-8:2 FTS | Yes | Yes | Yes |
| Perfluoro-1[13C8]octanesulfonamide | 13C8 FOSA | Yes | Yes | Yes |
| N-methyl-d3-perfluoro-1-octanesulfonamide | d3-NMePFOSA | Yes | Yes | Yes |
| N-ethyl-d5-perfluoro-1-octanesulfonamide | d5-NEtPFOSA | Yes | Yes | Yes |
| N-methyl-d3-perfluoro-1-octanesulfonamidoacetic acid | d3-NMeFOSAA | Yes | Yes | Yes |
| N-ethyl-d5-perfluoro-1-octanesulfonamidoacetic acid | d5-NEtFOSAA | Yes | Yes | Yes |
| N-methyl-d7-perfluorooctanesulfonamidoethanol | d7-N-MeFOSE-M | Yes | Yes | Yes |
| N-ethyl-d9-perfluorooctanesulfonamidoethanol | d9-N-EtFOSE-M | Yes | Yes | Yes |
| 13C2-2-Perfluorohexylethanoic acid | M2-6:2 FTCA | Yes | No | No |
| 13C2-2-Perfluorooctylethanoic acid | M2-8:2 FTCA | Yes | No | No |
| Bis(3,3,4,4,5,5,6,6,7,7,8,8,8-tridecafluorooctyl-1,2-13C2) hydrogen phosphate | 13C4-6:2 diPAP | No | No | Yes |
| Bis(3,3,4,4,5,5,6,6,7,7,8,8,9,9,10,10,10-heptadecafluorodecyl-1,2-13C2) hydrogen phosphate | 13C4-8:2 diPAP | No | No | Yes |

**Table S4**: Measured concentrations for all twelve WRRF from three Laboratories (ng/g dry basis)

Site 1

| **Lab** | **Lab 1** | | | | | | | |  | **Lab 2** | | | | | | | |  | **Lab 3** | | | | | | | |
| --- | --- | --- | --- | --- | --- | --- | --- | --- | --- | --- | --- | --- | --- | --- | --- | --- | --- | --- | --- | --- | --- | --- | --- | --- | --- | --- |
| **Acronym** | **(Location A)** | | | | **(Location B)** | | | |  | **(Location A)** | | | | **(Location B)** | | | |  | **(Location A)** | | | | **(Location B)** | | | |
|  | R | Q | RL | MDL | R | Q | RL | MDL |  | R | Q | RL | MDL | R | Q | RL | MDL |  | R | Q | RL | MDL | R | Q | RL | MDL |
| 3:3 FTCA | ND | U | 5.7 | 1.9 | ND | U | 14 | 4.7 |  | ND | U | 34.3 | 10.8 | ND | U | 75.9 | 23.9 |  | ND | U | 15.9 | 5.0 | ND | U | 15.6 | 4.9 |
| 5:3 FTCA | 22 | N/A | 5.7 | 1.9 | 220 | N/A | 14 | 4.7 |  | ND | U | 34.2 | 10.8 | ND | U | 75.8 | 23.9 |  | 146.5 | N/A | 15.8 | 5.0 | 615.7 | N/A | 15.5 | 4.9 |
| 7:3 FTCA | ND | U *- cn | 5.7 | 1.9 | 5.6 | J *- cn | 14 | 4.7 |  | ND | U | 34.2 | 10.8 | ND | U | 75.8 | 23.9 |  | 31.4 | N/A | 4.4 | 1.4 | 48.7 | N/A | 4.3 | 1.4 |
| 4:2 FTS | ND | U | 19 | 5.7 | ND | U | 47 | 14 |  | ND | U | 5.7 | 1.8 | ND | U | 12.6 | 4.0 |  | ND | U | 0.4 | 0.1 | ND | U | 0.4 | 0.1 |
| 6:2 FTS | ND | U | 19 | 5.7 | ND | U | 47 | 14 |  | ND | U | 9.9 | 3.1 | ND | U | 22.1 | 7.0 |  | ND | U | 0.5 | 0.2 | ND | U | 0.5 | 0.2 |
| 8:2 FTS | ND | U | 28 | 5.7 | ND | U | 70 | 14 |  | ND | U | 7.8 | 2.5 | ND | U | 17.3 | 5.5 |  | ND | U | 0.0 | 0.0 | 1.1 | N/A | 0.0 | 0.0 |
| PFMPA | ND | U | 5.7 | 1.9 | ND | U | 14 | 4.7 |  | ND | U | 7.1 | 2.2 | ND | U | 15.8 | 5.0 |  | ND | U | 2.3 | 0.7 | ND | U | 2.3 | 0.7 |
| PFMBA | ND | U | 5.7 | 1.9 | ND | U | 14 | 4.7 |  | ND | U | 3.5 | 1.1 | ND | U | 7.9 | 2.5 |  | ND | U | 4.5 | 1.4 | ND | U | 4.5 | 1.4 |
| NFDHA | ND | U | 5.7 | 1.9 | ND | U | 14 | 4.7 |  | ND | U | 6.4 | 2.0 | ND | U | 14.2 | 4.5 |  | ND | U | 0.8 | 0.3 | ND | U | 0.8 | 0.3 |
| HFPODA | ND | U | 19 | 9.5 | ND | U | 47 | 23 |  | ND | U | 8.5 | 2.7 | ND | U | 18.9 | 6.0 |  | ND | U | 17.1 | 5.4 | ND | U | 16.8 | 5.3 |
| DONA | ND | U | 28 | 1.9 | ND | U | 70 | 4.7 |  | ND | U | 7.1 | 2.2 | ND | U | 15.8 | 5.0 |  | ND | U | 1.2 | 0.4 | ND | U | 1.2 | 0.4 |
| NMeFOSAA | ND | U | 19 | 1.9 | ND | U | 47 | 4.7 |  | ND | U | 4.9 | 1.6 | ND | U | 11.0 | 3.5 |  | 1.2 | N/A | 0.4 | 0.1 | 3.5 | N/A | 0.4 | 0.1 |
| NEtFOSAA | 4.5 | J | 19 | 1.9 | 5.9 | J | 47 | 4.7 |  | ND | U | 3.5 | 1.1 | ND | U | 7.9 | 2.5 |  | 4.2 | N/A | 0.6 | 0.2 | 8.3 | N/A | 0.6 | 0.2 |
| PFOSA | ND | U | 5.7 | 1.9 | ND | U | 14 | 4.7 |  | ND | U | 2.8 | 0.9 | ND | U | 6.3 | 2.0 |  | ND | U | 0.4 | 0.1 | ND | U | 0.4 | 0.1 |
| NMeFOSA | ND | U | 19 | 4.7 | ND | U | 47 | 12 |  | ND | U | 2.8 | 0.9 | ND | U | 6.3 | 2.0 |  | ND | U | 3.6 | 1.2 | ND | U | 3.6 | 1.1 |
| NEtFOSA | ND | U | 19 | 4.7 | ND | U | 47 | 12 |  | ND | U | 2.8 | 0.9 | ND | U | 6.3 | 2.0 |  | ND | U | 4.0 | 1.3 | ND | U | 3.9 | 1.3 |
| NMeFOSE | ND | U | 19 | 4.7 | ND | U | 47 | 12 |  | ND | U | 28.5 | 9.0 | ND | U | 63.2 | 19.9 |  | ND | U | 11.3 | 3.6 | 26.1 | N/A | 11.1 | 3.5 |
| NEtFOSE | ND | U | 19 | 4.7 | ND | U | 47 | 12 |  | ND | U | 28.5 | 9.0 | ND | U | 63.2 | 19.9 |  | ND | U | 8.6 | 4.3 | ND | U | 8.5 | 4.3 |
| PFBA | ND | U | 19 | 7.6 | ND | U | 47 | 19 |  | ND | U | 9.2 | 2.9 | ND | U | 20.5 | 6.5 |  | ND | U | 28.2 | 14.1 | ND | U | 27.6 | 13.8 |
| PFPeA | ND | U | 5.7 | 1.9 | ND | U | 14 | 4.7 |  | ND | U | 5.7 | 1.8 | ND | U | 12.6 | 4.0 |  | ND | U | 7.6 | 2.4 | ND | U | 7.5 | 2.4 |
| PFHxA | 6.9 | N/A | 5.7 | 1.9 | 8.2 | J | 14 | 4.7 |  | ND | U | 8.5 | 2.7 | ND | U | 18.9 | 6.0 |  | ND | U | 12.6 | 4.0 | ND | U | 12.3 | 3.9 |
| PFHpA | ND | U | 5.7 | 1.9 | ND | U | 14 | 4.7 |  | ND | U | 4.2 | 1.3 | ND | U | 9.4 | 3.0 |  | ND | U | 5.2 | 1.6 | ND | U | 5.1 | 1.6 |
| PFOA | 2 | J | 5.7 | 1.9 | ND | U | 14 | 4.7 |  | ND | U | 4.9 | 1.6 | ND | U | 11.0 | 3.5 |  | ND | U | 5.5 | 1.7 | ND | U | 5.4 | 1.7 |
| PFNA | ND | U | 5.7 | 1.9 | ND | U | 14 | 4.7 |  | ND | U | 2.8 | 0.9 | ND | U | 6.3 | 2.0 |  | ND | U | 3.0 | 1.0 | ND | U | 3.0 | 0.9 |
| PFDA | ND | U | 5.7 | 1.9 | ND | U | 14 | 4.7 |  | ND | U | 3.5 | 1.1 | ND | U | 7.9 | 2.5 |  | ND | U | 3.1 | 1.0 | ND | U | 3.1 | 1.0 |
| PFUA | ND | U | 5.7 | 1.9 | ND | U | 14 | 4.7 |  | ND | U | 4.2 | 1.3 | ND | U | 9.4 | 3.0 |  | ND | U | 0.3 | 0.1 | 2.3 | N/A | 0.3 | 0.1 |
| PFDoA | ND | U | 5.7 | 1.9 | ND | U | 14 | 4.7 |  | ND | U | 5.7 | 1.8 | ND | U | 12.6 | 4.0 |  | ND | U | 0.7 | 0.2 | ND | U | 0.7 | 0.2 |
| PFTrDA | ND | U | 5.7 | 1.9 | ND | U | 14 | 4.7 |  | ND | U | 5.7 | 1.8 | ND | U | 12.6 | 4.0 |  | ND | U | 0.9 | 0.3 | ND | U | 0.9 | 0.3 |
| PFTeA | 2.7 | J | 5.7 | 1.9 | ND | U | 14 | 4.7 |  | ND | U | 12.1 | 3.8 | ND | U | 26.8 | 8.5 |  | ND | U | 3.9 | 1.2 | ND | U | 3.8 | 1.2 |
| PFBS | ND | U | 19 | 3.8 | ND | U | 47 | 9.3 |  | ND | U | 3.5 | 1.1 | ND | U | 7.9 | 2.5 |  | ND | U | 11.0 | 3.5 | ND | U | 10.8 | 3.4 |
| PFPS | ND | U | 5.7 | 1.9 | ND | U | 14 | 4.7 |  | ND | U | 4.9 | 1.6 | ND | U | 11.0 | 3.5 |  | ND | U | 2.3 | 0.7 | ND | U | 2.2 | 0.7 |
| PFHxS | ND | U | 5.7 | 1.9 | ND | U | 14 | 4.7 |  | ND | U | 7.8 | 2.5 | ND | U | 17.3 | 5.5 |  | ND | U | 4.7 | 1.5 | ND | U | 4.6 | 1.5 |
| PFHpS | ND | U | 5.7 | 1.9 | ND | U | 14 | 4.7 |  | ND | U | 5.7 | 1.8 | ND | U | 12.6 | 4.0 |  | ND | U | 2.4 | 0.8 | ND | U | 2.4 | 0.8 |
| PFOS | 9.7 | N/A | 5.7 | 1.9 | 38 | I | 14 | 4.7 |  | ND | U | 3.5 | 1.1 | 249.9 | N/A | 7.9 | 2.5 |  | ND | U | 16.2 | 5.1 | 50.5 | N/A | 15.9 | 5.0 |
| PFNS | ND | U | 5.7 | 1.9 | ND | U | 14 | 4.7 |  | ND | U | 10.7 | 3.4 | ND | U | 23.7 | 7.5 |  | ND | U | 0.6 | 0.2 | ND | U | 0.6 | 0.2 |
| PFDS | 5.6 | J I | 5.7 | 1.9 | ND | U | 14 | 4.7 |  | ND | U | 6.4 | 2.0 | ND | U | 14.2 | 4.5 |  | ND | U | 0.3 | 0.1 | ND | U | 0.3 | 0.1 |
| PFDoS | ND | U | 19 | 1.9 | ND | U | 47 | 4.7 |  | ND | U | 14.2 | 4.5 | ND | U | 31.6 | 9.9 |  | ND | U | 0.1 | 0.1 | ND | U | 0.1 | 0.1 |
| PFEESA/PES | ND | U | 5.7 | 1.9 | ND | U | 14 | 4.7 |  | ND | U | 2.1 | 0.7 | ND | U | 4.7 | 1.5 |  | ND | U | 17.6 | 5.5 | ND | U | 17.3 | 5.4 |
| 9Cl-PF3ONS | ND | U | 19 | 1.9 | ND | U | 47 | 4.7 |  | ND | U | 4.2 | 1.3 | ND | U | 9.4 | 3.0 |  | ND | U | 1.2 | 0.4 | ND | U | 1.2 | 0.4 |
| 11Cl-PF3OUdS | ND | U | 5.7 | 1.9 | ND | U | 14 | 4.7 |  | ND | U | 3.5 | 1.1 | ND | U | 7.9 | 2.5 |  | ND | U | 1.2 | 0.4 | ND | U | 1.2 | 0.4 |

Site 2

| **Lab** | **Lab 1** | | | | | | | |  | **Lab 2** | | | | | | | |  | **Lab 3** | | | | | | | |
| --- | --- | --- | --- | --- | --- | --- | --- | --- | --- | --- | --- | --- | --- | --- | --- | --- | --- | --- | --- | --- | --- | --- | --- | --- | --- | --- |
| **Acronym** | **(Location A)** | | | | **(Location B)** | | | |  | **(Location A)** | | | | **(Location B)** | | | |  | **(Location A)** | | | | **(Location B)** | | | |
|  | R | Q | RL | MDL | R | Q | RL | MDL |  | R | Q | RL | MDL | R | Q | RL | MDL |  | R | Q | RL | MDL | R | Q | RL | MDL |
| 3:3 FTCA | ND | U | 11 | 3.7 | ND | U | 22 | 7.3 |  | ND | U | 57.3 | 18.0 | ND | U | 103.2 | 32.5 |  | ND | U | 17.0 | 5.4 | ND | U | 30.6 | 9.6 |
| 5:3 FTCA | 13 | N/A | 11 | 3.7 | 200 | N/A | 22 | 7.3 |  | ND | U | 57.3 | 18.0 | ND | U | 103.2 | 32.5 |  | 104.5 | N/A | 17.0 | 5.4 | 523.2 | N/A | 30.6 | 9.6 |
| 7:3 FTCA | ND | U *- | 11 | 3.7 | ND | U *- | 22 | 7.3 |  | ND | U | 57.3 | 18.0 | ND | U | 103.2 | 32.5 |  | 19.8 | N/A | 4.7 | 1.5 | 31.5 | N/A | 8.5 | 2.7 |
| 4:2 FTS | ND | U | 37 | 11 | ND | U | 73 | 22 |  | ND | U | 9.5 | 3.0 | ND | U | 17.2 | 5.4 |  | ND | U | 0.4 | 0.2 | ND | U | 0.8 | 0.3 |
| 6:2 FTS | ND | U | 37 | 11 | ND | U | 73 | 22 |  | ND | U | 16.7 | 5.3 | ND | U | 30.1 | 9.5 |  | ND | U | 0.5 | 0.2 | ND | U | 1.0 | 0.3 |
| 8:2 FTS | ND | U | 56 | 11 | ND | U | 110 | 22 |  | ND | U | 13.1 | 4.1 | ND | U | 23.6 | 7.4 |  | ND | U | 0.0 | 0.0 | ND | U | 0.1 | 0.1 |
| PFMPA | ND | U | 11 | 3.7 | ND | U | 22 | 7.3 |  | ND | U | 11.9 | 3.8 | ND | U | 21.5 | 6.8 |  | ND | U | 2.5 | 0.8 | ND | U | 4.5 | 1.4 |
| PFMBA | ND | U | 11 | 3.7 | ND | U | 22 | 7.3 |  | ND | U | 5.9 | 1.9 | ND | U | 10.7 | 3.4 |  | ND | U | 4.9 | 1.5 | ND | U | 8.8 | 2.8 |
| NFDHA | ND | U | 11 | 3.7 | ND | U | 22 | 7.3 |  | ND | U | 10.7 | 3.4 | ND | U | 19.3 | 6.1 |  | ND | U | 0.9 | 0.3 | ND | U | 1.6 | 0.5 |
| HFPODA | ND | U | 37 | 19 | ND | U | 73 | 36 |  | ND | U | 14.3 | 4.5 | ND | U | 25.8 | 8.1 |  | ND | U | 18.3 | 5.8 | ND | U | 33.0 | 10.4 |
| DONA | ND | U | 56 | 3.7 | ND | U | 110 | 7.3 |  | ND | U | 11.9 | 3.8 | ND | U | 21.5 | 6.8 |  | ND | U | 1.3 | 0.4 | ND | U | 2.3 | 0.7 |
| NMeFOSAA | ND | U | 37 | 3.7 | ND | U | 73 | 7.3 |  | ND | U | 8.3 | 2.6 | ND | U | 15.0 | 4.7 |  | 0.51 | N/A | 0.4 | 0.1 | 1.2 | N/A | 0.8 | 0.3 |
| NEtFOSAA | ND | U | 37 | 3.7 | ND | U | 73 | 7.3 |  | ND | U | 5.9 | 1.9 | ND | U | 10.7 | 3.4 |  | 2.01 | N/A | 0.7 | 0.2 | 5.29 | N/A | 1.2 | 0.4 |
| PFOSA | ND | U | 11 | 3.7 | ND | U | 22 | 7.3 |  | ND | U | 4.7 | 1.5 | ND | U | 8.6 | 2.7 |  | ND | U | 0.4 | 0.1 | ND | U | 0.8 | 0.3 |
| NMeFOSA | ND | U | 37 | 9.4 | ND | U | 73 | 18 |  | ND | U | 4.7 | 1.5 | ND | U | 8.6 | 2.7 |  | ND | U | 3.9 | 1.2 | ND | U | 7.1 | 2.2 |
| NEtFOSA | ND | U | 37 | 9.4 | ND | U | 73 | 18 |  | ND | U | 4.7 | 1.5 | ND | U | 8.6 | 2.7 |  | ND | U | 4.3 | 1.4 | ND | U | 7.8 | 2.5 |
| NMeFOSE | ND | U | 37 | 9.4 | ND | U | 73 | 18 |  | ND | U | 47.7 | 15.0 | ND | U | 86.0 | 27.0 |  | 24.6 | N/A | 12.1 | 3.8 | 25.6 | N/A | 21.8 | 6.9 |
| NEtFOSE | ND | U | 37 | 9.4 | ND | U | 73 | 18 |  | ND | U | 47.7 | 15.0 | ND | U | 86.0 | 27.0 |  | ND | U | 9.2 | 4.6 | ND | U | 16.6 | 8.3 |
| PFBA | ND | U | 37 | 15 | ND | U | 73 | 29 |  | ND | U | 15.5 | 4.9 | ND | U | 27.9 | 8.8 |  | ND | U | 30.2 | 15.1 | ND | U | 54.3 | 27.2 |
| PFPeA | ND | U | 11 | 3.7 | ND | U | 22 | 7.3 |  | ND | U | 9.5 | 3.0 | ND | U | 17.2 | 5.4 |  | ND | U | 8.2 | 2.6 | ND | U | 14.7 | 4.6 |
| PFHxA | ND | U | 11 | 3.7 | ND | U | 22 | 7.3 |  | ND | U | 14.3 | 4.5 | ND | U | 25.8 | 8.1 |  | ND | U | 13.5 | 4.3 | ND | U | 24.3 | 7.6 |
| PFHpA | ND | U | 11 | 3.7 | ND | U | 22 | 7.3 |  | ND | U | 7.1 | 2.3 | ND | U | 12.9 | 4.1 |  | ND | U | 5.6 | 1.8 | ND | U | 10.0 | 3.2 |
| PFOA | ND | U | 11 | 3.7 | ND | U | 22 | 7.3 |  | ND | U | 8.3 | 2.6 | ND | U | 15.0 | 4.7 |  | ND | U | 5.9 | 1.9 | ND | U | 10.6 | 3.4 |
| PFNA | ND | U | 11 | 3.7 | ND | U | 22 | 7.3 |  | ND | U | 4.7 | 1.5 | ND | U | 8.6 | 2.7 |  | ND | U | 3.2 | 1.0 | ND | U | 5.8 | 1.9 |
| PFDA | ND | U | 11 | 3.7 | ND | U | 22 | 7.3 |  | ND | U | 5.9 | 1.9 | ND | U | 10.7 | 3.4 |  | ND | U | 3.4 | 1.1 | ND | U | 6.1 | 1.9 |
| PFUA | ND | U | 11 | 3.7 | ND | U | 22 | 7.3 |  | ND | U | 7.1 | 2.3 | ND | U | 12.9 | 4.1 |  | ND | U | 0.3 | 0.1 | 1.58 | N/A | 0.5 | 0.2 |
| PFDoA | ND | U | 11 | 3.7 | ND | U | 22 | 7.3 |  | ND | U | 9.5 | 3.0 | ND | U | 17.2 | 5.4 |  | ND | U | 0.7 | 0.2 | 4.90 | N/A | 1.4 | 0.4 |
| PFTrDA | ND | U | 11 | 3.7 | ND | U | 22 | 7.3 |  | ND | U | 9.5 | 3.0 | ND | U | 17.2 | 5.4 |  | ND | U | 1.0 | 0.3 | ND | U | 1.9 | 0.6 |
| PFTeA | ND | U | 11 | 3.7 | ND | U | 22 | 7.3 |  | ND | U | 20.2 | 6.4 | ND | U | 36.5 | 11.5 |  | ND | U | 4.1 | 1.3 | ND | U | 7.5 | 2.4 |
| PFBS | ND | U | 37 | 7.5 | ND | U | 73 | 15 |  | ND | U | 5.9 | 1.9 | ND | U | 10.7 | 3.4 |  | ND | U | 11.8 | 3.7 | ND | U | 21.3 | 6.7 |
| PFPS | ND | U | 11 | 3.7 | ND | U | 22 | 7.3 |  | ND | U | 8.3 | 2.6 | ND | U | 15.0 | 4.7 |  | ND | U | 2.4 | 0.8 | ND | U | 4.4 | 1.4 |
| PFHxS | ND | U | 11 | 3.7 | ND | U | 22 | 7.3 |  | ND | U | 13.1 | 4.1 | ND | U | 23.6 | 7.4 |  | ND | U | 5.0 | 1.6 | ND | U | 9.1 | 2.9 |
| PFHpS | ND | U | 11 | 3.7 | ND | U | 22 | 7.3 |  | ND | U | 9.5 | 3.0 | ND | U | 17.2 | 5.4 |  | ND | U | 2.6 | 0.8 | ND | U | 4.7 | 1.5 |
| PFOS | ND | U | 11 | 3.7 | 8.2 | J | 22 | 7.3 |  | 61.2 | N/A | 5.9 | 1.9 | ND | U | 10.7 | 3.4 |  | ND | U | 17.4 | 5.5 | ND | U | 31.2 | 9.8 |
| PFNS | ND | U | 11 | 3.7 | ND | U | 22 | 7.3 |  | ND | U | 17.9 | 5.6 | ND | U | 32.2 | 10.1 |  | ND | U | 0.6 | 0.2 | ND | U | 1.2 | 0.4 |
| PFDS | ND | U | 11 | 3.7 | ND | U | 22 | 7.3 |  | ND | U | 10.7 | 3.4 | ND | U | 19.3 | 6.1 |  | ND | U | 0.3 | 0.1 | ND | U | 0.7 | 0.2 |
| PFDoS | ND | U | 37 | 3.7 | ND | U | 73 | 7.3 |  | ND | U | 23.8 | 7.5 | ND | U | 43.0 | 13.5 |  | ND | U | 0.1 | 0.1 | ND | U | 0.3 | 0.1 |
| PFEESA/PES | ND | U | 11 | 3.7 | ND | U | 22 | 7.3 |  | ND | U | 3.5 | 1.1 | ND | U | 6.4 | 2.0 |  | ND | U | 18.9 | 5.9 | ND | U | 33.9 | 10.7 |
| 9Cl-PF3ONS | ND | U | 37 | 3.7 | ND | U | 73 | 7.3 |  | ND | U | 7.1 | 2.3 | ND | U | 12.9 | 4.1 |  | ND | U | 1.3 | 0.4 | ND | U | 2.3 | 0.7 |
| 11Cl-PF3OUdS | ND | U | 11 | 3.7 | ND | U | 22 | 7.3 |  | ND | U | 5.9 | 1.9 | ND | U | 10.7 | 3.4 |  | ND | U | 1.3 | 0.4 | ND | U | 2.3 | 0.7 |

Site 3

| **Lab** | **Lab 1** | | | | | | | |  | **Lab 2** | | | | | | | |  | **Lab 3** | | | | | | | |
| --- | --- | --- | --- | --- | --- | --- | --- | --- | --- | --- | --- | --- | --- | --- | --- | --- | --- | --- | --- | --- | --- | --- | --- | --- | --- | --- |
| **Acronym** | **(Location A)** | | | | **(Location B)** | | | |  | **(Location A)** | | | | **(Location B)** | | | |  | **(Location A)** | | | | **(Location B)** | | | |
|  | R | Q | RL | MDL | R | Q | RL | MDL |  | R | Q | RL | MDL | R | Q | RL | MDL |  | R | Q | RL | MDL | R | Q | RL | MDL |
| 3:3 FTCA | 3.4 | U | 10 | 3.4 | 7.9 | U | 24 | 7.9 |  | ND | U | 67.6 | 21.3 | ND | U | 102.8 | 32.3 |  | ND | U | 28.50 | 8.96 | ND | U | 10.80 | 3.40 |
| 5:3 FTCA | 16 | N/A | 10 | 3.4 | 160 | N/A | 24 | 7.9 |  | ND | U | 67.6 | 21.3 | ND | U | 102.8 | 32.3 |  | 177.0 | N/A | 28.50 | 8.96 | 304 | N/A | 10.80 | 3.40 |
| 7:3 FTCA | 3.4 | U *- cn | 10 | 3.4 | 7.9 | U *- cn | 24 | 7.9 |  | ND | U | 67.6 | 21.3 | ND | U | 102.8 | 32.3 |  | 11.6 | N/A | 7.90 | 2.51 | 20.6 | N/A | 3.00 | 0.95 |
| 4:2 FTS | 10 | U | 34 | 10 | 24 | U | 79 | 24 |  | ND | U | 11.2 | 3.5 | ND | U | 17.1 | 5.4 |  | ND | U | 0.80 | 0.26 | ND | U | 0.30 | 0.10 |
| 6:2 FTS | 10 | U | 34 | 10 | 24 | U | 79 | 24 |  | ND | U | 19.7 | 6.2 | ND | U | 29.9 | 9.4 |  | ND | U | 0.90 | 0.29 | ND | U | 0.30 | 0.11 |
| 8:2 FTS | 10 | U | 51 | 10 | 24 | U | 120 | 24 |  | ND | U | 15.5 | 4.9 | ND | U | 23.5 | 7.4 |  | ND | U | 0.10 | 0.05 | 0.34 | N/A | 0.00 | 0.02 |
| PFMPA | 3.4 | U | 10 | 3.4 | 7.9 | U | 24 | 7.9 |  | ND | U | 14.1 | 4.4 | ND | U | 21.4 | 6.7 |  | ND | U | 4.20 | 1.33 | ND | U | 1.60 | 0.50 |
| PFMBA | 3.4 | U | 10 | 3.4 | 7.9 | U | 24 | 7.9 |  | ND | U | 7.0 | 2.2 | ND | U | 10.7 | 3.4 |  | ND | U | 8.20 | 2.59 | ND | U | 3.10 | 0.98 |
| NFDHA | 3.4 | U | 10 | 3.4 | 7.9 | U | 24 | 7.9 |  | ND | U | 12.6 | 4.0 | ND | U | 19.2 | 6.1 |  | ND | U | 1.50 | 0.48 | ND | U | 0.50 | 0.18 |
| HFPODA | 17 | U | 34 | 17 | 40 | U | 79 | 40 |  | ND | U | 16.9 | 5.3 | ND | U | 25.7 | 8.1 |  | ND | U | 30.70 | 9.67 | ND | U | 11.60 | 3.67 |
| DONA | 3.4 | U | 51 | 3.4 | 7.9 | U | 120 | 7.9 |  | ND | U | 14.1 | 4.4 | ND | U | 21.4 | 6.7 |  | ND | U | 2.10 | 0.69 | ND | U | 0.80 | 0.26 |
| NMeFOSAA | 3.4 | U | 34 | 3.4 | 7.9 | U | 79 | 7.9 |  | ND | U | 9.8 | 3.1 | ND | U | 14.9 | 4.7 |  | 3.8 | N/A | 0.70 | 0.24 | 7.1 | N/A | 0.20 | 0.09 |
| NEtFOSAA | 3.4 | U | 34 | 3.4 | 7.9 | U | 79 | 7.9 |  | ND | U | 7.0 | 2.2 | ND | U | 10.7 | 3.4 |  | 3.9 | N/A | 1.20 | 0.38 | 4.91 | N/A | 0.40 | 0.14 |
| PFOSA | 3.4 | U | 10 | 3.4 | 7.9 | U | 24 | 7.9 |  | ND | U | 5.6 | 1.8 | ND | U | 8.5 | 2.7 |  | ND | U | 0.70 | 0.24 | ND | U | 0.20 | 0.09 |
| NMeFOSA | 8.5 | U | 34 | 8.5 | 20 | U | 79 | 20 |  | ND | U | 5.6 | 1.8 | ND | U | 8.5 | 2.7 |  | ND | U | 6.60 | 2.09 | ND | U | 2.50 | 0.79 |
| NEtFOSA | 8.5 | U | 34 | 8.5 | 20 | U | 79 | 20 |  | ND | U | 5.6 | 1.8 | ND | U | 8.5 | 2.7 |  | ND | U | 7.20 | 2.29 | ND | U | 2.70 | 0.87 |
| NMeFOSE | 8.5 | U | 34 | 8.5 | 20 | U | 79 | 20 |  | ND | U | 56.4 | 17.7 | ND | U | 85.6 | 26.9 |  | ND | U | 20.30 | 6.40 | ND | U | 7.70 | 2.43 |
| NEtFOSE | 8.5 | U | 34 | 8.5 | 20 | U | 79 | 20 |  | ND | U | 56.4 | 17.7 | ND | U | 85.6 | 26.9 |  | ND | U | 15.50 | 7.77 | ND | U | 5.80 | 2.95 |
| PFBA | 14 | U | 34 | 14 | 32 | U | 79 | 32 |  | ND | U | 18.3 | 5.8 | ND | U | 27.8 | 8.8 |  | ND | U | 50.60 | 25.31 | ND | U | 19.10 | 9.59 |
| PFPeA | 3.4 | U | 10 | 3.4 | 7.9 | U | 24 | 7.9 |  | ND | U | 11.2 | 3.5 | ND | U | 17.1 | 5.4 |  | ND | U | 13.70 | 4.33 | ND | U | 5.20 | 1.64 |
| PFHxA | 3.5 | J I | 10 | 3.4 | 8 | J | 24 | 7.9 |  | ND | U | 16.9 | 5.3 | ND | U | 25.7 | 8.1 |  | ND | U | 22.60 | 7.12 | ND | U | 8.50 | 2.70 |
| PFHpA | 3.4 | U | 10 | 3.4 | 7.9 | U | 24 | 7.9 |  | ND | U | 8.4 | 2.7 | ND | U | 12.8 | 4.0 |  | ND | U | 9.30 | 2.95 | ND | U | 3.50 | 1.12 |
| PFOA | 3.4 | U | 10 | 3.4 | 7.9 | U | 24 | 7.9 |  | ND | U | 9.8 | 3.1 | ND | U | 14.9 | 4.7 |  | ND | U | 9.90 | 3.12 | ND | U | 3.70 | 1.18 |
| PFNA | 3.4 | U | 10 | 3.4 | 7.9 | U | 24 | 7.9 |  | ND | U | 5.6 | 1.8 | ND | U | 8.5 | 2.7 |  | ND | U | 5.40 | 1.72 | ND | U | 2.00 | 0.65 |
| PFDA | 3.4 | U | 10 | 3.4 | 7.9 | U | 24 | 7.9 |  | ND | U | 7.0 | 2.2 | ND | U | 10.7 | 3.4 |  | ND | U | 5.70 | 1.79 | ND | U | 2.10 | 0.68 |
| PFUA | 3.4 | U | 10 | 3.4 | 7.9 | U | 24 | 7.9 |  | ND | U | 8.4 | 2.7 | ND | U | 12.8 | 4.0 |  | ND | U | 0.50 | 0.17 | 2.7 | N/A | 0.20 | 0.07 |
| PFDoA | 3.4 | U | 10 | 3.4 | 7.9 | U | 24 | 7.9 |  | ND | U | 11.2 | 3.5 | ND | U | 17.1 | 5.4 |  | ND | U | 1.30 | 0.41 | ND | U | 0.40 | 0.16 |
| PFTrDA | 3.4 | U | 10 | 3.4 | 7.9 | U | 24 | 7.9 |  | ND | U | 11.2 | 3.5 | ND | U | 17.1 | 5.4 |  | ND | U | 1.70 | 0.56 | ND | U | 0.60 | 0.21 |
| PFTeA | 3.4 | U | 10 | 3.4 | 7.9 | U | 24 | 7.9 |  | ND | U | 23.9 | 7.5 | ND | U | 36.4 | 11.5 |  | ND | U | 7.00 | 2.20 | ND | U | 2.60 | 0.84 |
| PFBS | 6.8 | U | 34 | 6.8 | 16 | U | 79 | 16 |  | ND | U | 7.0 | 2.2 | ND | U | 10.7 | 3.4 |  | ND | U | 19.80 | 6.24 | ND | U | 7.50 | 2.37 |
| PFPS | 3.4 | U | 10 | 3.4 | 7.9 | U | 24 | 7.9 |  | ND | U | 9.8 | 3.1 | ND | U | 14.9 | 4.7 |  | ND | U | 4.10 | 1.31 | ND | U | 1.50 | 0.50 |
| PFHxS | 4.9 | J I | 10 | 3.4 | 16 | J I | 24 | 7.9 |  | ND | U | 15.5 | 4.9 | ND | U | 23.5 | 7.4 |  | ND | U | 8.40 | 2.67 | ND | U | 3.20 | 1.01 |
| PFHpS | 3.4 | U | 10 | 3.4 | 7.9 | U | 24 | 7.9 |  | ND | U | 11.2 | 3.5 | ND | U | 17.1 | 5.4 |  | ND | U | 4.30 | 1.38 | ND | U | 1.60 | 0.52 |
| PFOS | 4.8 | J | 10 | 3.4 | 20 | J I | 24 | 7.9 |  | ND | U | 7.0 | 2.2 | 96.2 | N/A | 10.7 | 3.4 |  | ND | U | 29.10 | 9.16 | ND | U | 11.00 | 3.47 |
| PFNS | 3.4 | U | 10 | 3.4 | 7.9 | U | 24 | 7.9 |  | ND | U | 21.1 | 6.7 | ND | U | 32.1 | 10.1 |  | ND | U | 1.10 | 0.36 | ND | U | 0.40 | 0.14 |
| PFDS | 3.4 | U | 10 | 3.4 | 7.9 | U | 24 | 7.9 |  | ND | U | 12.6 | 4.0 | ND | U | 19.2 | 6.1 |  | ND | U | 0.60 | 0.21 | ND | U | 0.20 | 0.08 |
| PFDoS | 3.4 | U | 34 | 3.4 | 7.9 | U | 79 | 7.9 |  | ND | U | 28.2 | 8.9 | ND | U | 42.8 | 13.5 |  | ND | U | 0.30 | 0.10 | ND | U | 0.10 | 0.04 |
| PFEESA/PES | 3.4 | U | 10 | 3.4 | 7.9 | U | 24 | 7.9 |  | ND | U | 4.2 | 1.3 | ND | U | 6.4 | 2.0 |  | ND | U | 31.60 | 9.95 | ND | U | 11.90 | 3.77 |
| 9Cl-PF3ONS | 3.4 | U | 34 | 3.4 | 7.9 | U | 79 | 7.9 |  | ND | U | 8.4 | 2.7 | ND | U | 12.8 | 4.0 |  | ND | U | 2.10 | 0.69 | ND | U | 0.80 | 0.26 |
| 11Cl-PF3OUdS | 3.4 | U | 10 | 3.4 | 7.9 | U | 24 | 7.9 |  | ND | U | 7.0 | 2.2 | ND | U | 10.7 | 3.4 |  | ND | U | 2.10 | 0.69 | ND | U | 0.80 | 0.26 |

Site 4

| **Lab** | **Lab 1** | | | | | | | |  | **Lab 2** | | | | | | | |  | **Lab 3** | | | | | | | |
| --- | --- | --- | --- | --- | --- | --- | --- | --- | --- | --- | --- | --- | --- | --- | --- | --- | --- | --- | --- | --- | --- | --- | --- | --- | --- | --- |
| **Acronym** | **(Location A)** | | | | **(Location B)** | | | |  | **(Location A)** | | | | **(Location B)** | | | |  | **(Location A)** | | | | **(Location B)** | | | |
|  | R | Q | RL | MDL | R | Q | RL | MDL |  | R | Q | RL | MDL | R | Q | RL | MDL |  | R | Q | RL | MDL | R | Q | RL | MDL |
| 3:3 FTCA | 4.6 | U *- cn | 14 | 4.6 | 0.94 | U *- cn | 2.8 | 0.94 |  | ND | U | 6.0 | 1.9 | ND | U | 7.1 | 2.2 |  | ND | U | 8.3 | 2.64 | ND | U | 8.3 | 2.64 |
| 5:3 FTCA | 44 | *- cn | 14 | 4.6 | 86 | *- cn | 2.8 | 0.94 |  | ND | U | 6.0 | 1.9 | 19.3 | N/A | 7.1 | 2.2 |  | 187.2 | N/A | 8.3 | 2.64 | 235.0 | N/A | 8.3 | 2.64 |
| 7:3 FTCA | 4.6 | U *- cn | 14 | 4.6 | 6.7 | *- cn | 2.8 | 0.94 |  | ND | U | 6.0 | 1.9 | ND | U | 7.1 | 2.2 |  | 23.0 | N/A | 2.3 | 0.74 | 56.3 | N/A | 2.3 | 0.74 |
| 4:2 FTS | 14 | U | 46 | 14 | 2.8 | U | 9.4 | 2.8 |  | ND | U | 1.0 | 0.3 | ND | U | 1.1 | 0.4 |  | ND | U | 0.2 | 0.08 | ND | U | 0.2 | 0.08 |
| 6:2 FTS | 14 | U | 46 | 14 | 2.8 | U | 9.4 | 2.8 |  | ND | U | 1.7 | 0.6 | ND | U | 2.0 | 0.7 |  | ND | U | 0.2 | 0.09 | ND | U | 0.2 | 0.09 |
| 8:2 FTS | 14 | U | 69 | 14 | 2.8 | U | 14 | 2.8 |  | ND | U | 1.3 | 0.4 | ND | U | 1.6 | 0.5 |  | ND | U | 0.0 | 0.02 | ND | U | 0.0 | 0.02 |
| PFMPA | 4.6 | U | 14 | 4.6 | 0.94 | U | 2.8 | 0.94 |  | ND | U | 1.2 | 0.4 | ND | U | 1.4 | 0.5 |  | ND | U | 1.2 | 0.39 | ND | U | 1.2 | 0.39 |
| PFMBA | 4.6 | U *- cn | 14 | 4.6 | 0.94 | U *- cn | 2.8 | 0.94 |  | ND | U | 0.6 | 0.2 | ND | U | 0.7 | 0.2 |  | ND | U | 2.4 | 0.76 | ND | U | 2.4 | 0.76 |
| NFDHA | 4.6 | U *- cn | 14 | 4.6 | 0.94 | U *- cn | 2.8 | 0.94 |  | ND | U | 1.1 | 0.4 | ND | U | 1.3 | 0.4 |  | ND | U | 0.4 | 0.14 | ND | U | 0.4 | 0.14 |
| HFPODA | 23 | U | 46 | 23 | 4.7 | U | 9.4 | 4.7 |  | ND | U | 1.5 | 0.5 | ND | U | 1.7 | 0.6 |  | ND | U | 9.0 | 2.85 | ND | U | 9.0 | 2.85 |
| DONA | 4.6 | U | 69 | 4.6 | 0.94 | U | 14 | 0.94 |  | ND | U | 1.2 | 0.4 | ND | U | 1.4 | 0.5 |  | ND | U | 0.6 | 0.20 | ND | U | 0.6 | 0.20 |
| NMeFOSAA | 6.1 | J | 46 | 4.6 | 12 | N/A | 9.4 | 0.94 |  | ND | U | 0.8 | 0.3 | ND | U | 1.0 | 0.3 |  | 5.0 | N/A | 0.2 | 0.07 | 13.9 | N/A | 0.2 | 0.07 |
| NEtFOSAA | 4.6 | U | 46 | 4.6 | 7.2 | J | 9.4 | 0.94 |  | ND | U | 0.6 | 0.2 | ND | U | 0.7 | 0.2 |  | 2.8 | N/A | 0.3 | 0.11 | 8.97 | N/A | 0.3 | 0.11 |
| PFOSA | 4.6 | U | 14 | 4.6 | 0.94 | U | 2.8 | 0.94 |  | ND | U | 0.5 | 0.2 | ND | U | 0.5 | 0.2 |  | ND | U | 0.2 | 0.07 | ND | U | 0.2 | 0.07 |
| NMeFOSA | 11 | U | 46 | 11 | 2.4 | U | 9.4 | 2.4 |  | ND | U | 0.5 | 0.2 | ND | U | 0.5 | 0.2 |  | ND | U | 1.9 | 0.61 | ND | U | 1.9 | 0.61 |
| NEtFOSA | 11 | U | 46 | 11 | 2.4 | U | 9.4 | 2.4 |  | ND | U | 0.5 | 0.2 | ND | U | 0.5 | 0.2 |  | ND | U | 2.1 | 0.68 | ND | U | 2.1 | 0.68 |
| NMeFOSE | 11 | U | 46 | 11 | 7.6 | J | 9.4 | 2.4 |  | ND | U | 5.0 | 1.6 | ND | U | 5.9 | 1.9 |  | 16.0 | N/A | 5.9 | 1.88 | 19.3 | N/A | 5.9 | 1.88 |
| NEtFOSE | 11 | U | 46 | 11 | 3.1 | J | 9.4 | 2.4 |  | ND | U | 5.0 | 1.6 | ND | U | 5.9 | 1.9 |  | ND | U | 4.5 | 2.29 | ND | U | 4.5 | 2.29 |
| PFBA | 18 | U | 46 | 18 | 3.8 | U | 9.4 | 3.8 |  | ND | U | 1.6 | 0.5 | ND | U | 1.9 | 0.6 |  | ND | U | 14.9 | 7.45 | ND | U | 14.9 | 7.45 |
| PFPeA | 4.6 | U | 14 | 4.6 | 0.94 | U | 2.8 | 0.94 |  | ND | U | 1.0 | 0.3 | ND | U | 1.1 | 0.4 |  | ND | U | 4.0 | 1.27 | ND | U | 4.0 | 1.27 |
| PFHxA | 4.6 | J I | 14 | 4.6 | 4.7 | N/A | 2.8 | 0.94 |  | ND | U | 1.5 | 0.5 | ND | U | 1.7 | 0.6 |  | 32.6 | N/A | 6.6 | 2.10 | 34.7 | N/A | 6.6 | 2.10 |
| PFHpA | 4.6 | U | 14 | 4.6 | 0.94 | U | 2.8 | 0.94 |  | ND | U | 0.7 | 0.2 | ND | U | 0.8 | 0.3 |  | ND | U | 2.7 | 0.87 | ND | U | 2.7 | 0.87 |
| PFOA | 4.6 | U | 14 | 4.6 | 0.94 | U | 2.8 | 0.94 |  | ND | U | 0.8 | 0.3 | ND | U | 1.0 | 0.3 |  | ND | U | 2.9 | 0.92 | ND | U | 2.9 | 0.92 |
| PFNA | 4.6 | U | 14 | 4.6 | 0.94 | U | 2.8 | 0.94 |  | ND | U | 0.5 | 0.2 | ND | U | 0.5 | 0.2 |  | ND | U | 1.6 | 0.51 | ND | U | 1.6 | 0.51 |
| PFDA | 4.6 | U | 14 | 4.6 | 3.1 | N/A | 2.8 | 0.94 |  | ND | U | 0.6 | 0.2 | ND | U | 0.7 | 0.2 |  | 3 | N/A | 1.6 | 0.53 | 5.0 | N/A | 1.6 | 0.53 |
| PFUA | 4.6 | U | 14 | 4.6 | 1.2 | J | 2.8 | 0.94 |  | ND | U | 0.7 | 0.2 | ND | U | 0.8 | 0.3 |  | 1.1 | N/A | 0.1 | 0.05 | 1.3 | N/A | 0.1 | 0.05 |
| PFDoA | 4.6 | U | 14 | 4.6 | 1.6 | J | 2.8 | 0.94 |  | ND | U | 1.0 | 0.3 | ND | U | 1.1 | 0.4 |  | ND | U | 0.3 | 0.12 | ND | U | 0.3 | 0.12 |
| PFTrDA | 4.6 | U | 14 | 4.6 | 0.94 | U | 2.8 | 0.94 |  | ND | U | 1.0 | 0.3 | ND | U | 1.1 | 0.4 |  | ND | U | 0.5 | 0.16 | ND | U | 0.5 | 0.16 |
| PFTeA | 4.6 | U | 14 | 4.6 | 0.94 | U | 2.8 | 0.94 |  | ND | U | 2.1 | 0.7 | ND | U | 2.5 | 0.8 |  | ND | U | 2.0 | 0.65 | ND | U | 2.0 | 0.65 |
| PFBS | 9.1 | U | 46 | 9.1 | 1.9 | U | 9.4 | 1.9 |  | ND | U | 0.6 | 0.2 | ND | U | 0.7 | 0.2 |  | ND | U | 5.8 | 1.84 | ND | U | 5.8 | 1.84 |
| PFPS | 4.6 | U | 14 | 4.6 | 0.94 | U | 2.8 | 0.94 |  | ND | U | 0.8 | 0.3 | ND | U | 1.0 | 0.3 |  | ND | U | 1.2 | 0.39 | ND | U | 1.2 | 0.39 |
| PFHxS | 4.6 | U | 14 | 4.6 | 0.94 | U | 2.8 | 0.94 |  | ND | U | 1.3 | 0.4 | ND | U | 1.6 | 0.5 |  | ND | U | 2.5 | 0.79 | ND | U | 2.5 | 0.79 |
| PFHpS | 4.6 | U | 14 | 4.6 | 0.94 | U | 2.8 | 0.94 |  | ND | U | 1.0 | 0.3 | ND | U | 1.1 | 0.4 |  | ND | U | 1.2 | 0.41 | ND | U | 1.2 | 0.41 |
| PFOS | 4.9 | J | 14 | 4.6 | 8.7 | N/A | 2.8 | 0.94 |  | 14.9 | N/A | 0.6 | 0.2 | 66.6 | N/A | 0.7 | 0.2 |  | ND | U | 8.5 | 2.70 | ND | U | 8.5 | 2.70 |
| PFNS | 4.6 | U | 14 | 4.6 | 0.94 | U | 2.8 | 0.94 |  | ND | U | 1.8 | 0.6 | ND | U | 2.2 | 0.7 |  | ND | U | 0.3 | 0.11 | ND | U | 0.3 | 0.11 |
| PFDS | 4.6 | U | 14 | 4.6 | 1.8 | J I | 2.8 | 0.94 |  | ND | U | 1.1 | 0.4 | ND | U | 1.3 | 0.4 |  | ND | U | 0.1 | 0.06 | ND | U | 0.1 | 0.06 |
| PFDoS | 4.6 | U | 46 | 4.6 | 0.94 | U | 9.4 | 0.94 |  | ND | U | 2.5 | 0.8 | ND | U | 2.9 | 0.9 |  | ND | U | 0.0 | 0.03 | ND | U | 0.0 | 0.03 |
| PFEESA/PES | 4.6 | U | 14 | 4.6 | 0.94 | U | 2.8 | 0.94 |  | ND | U | 0.3 | 0.1 | ND | U | 0.4 | 0.1 |  | ND | U | 9.3 | 2.93 | ND | U | 9.3 | 2.93 |
| 9Cl-PF3ONS | 4.6 | U | 46 | 4.6 | 0.94 | U | 9.4 | 0.94 |  | ND | U | 0.7 | 0.2 | ND | U | 0.8 | 0.3 |  | ND | U | 0.6 | 0.20 | ND | U | 0.6 | 0.20 |
| 11Cl-PF3OUdS | 4.6 | U | 14 | 4.6 | 0.94 | U | 2.8 | 0.94 |  | ND | U | 0.6 | 0.2 | ND | U | 0.7 | 0.2 |  | ND | U | 0.6 | 0.20 | ND | U | 0.6 | 0.20 |

Site 5

| **Lab** | **Lab 1** | | | | | | | |  | **Lab 2** | | | | | | | |  | **Lab 3** | | | | | | | |
| --- | --- | --- | --- | --- | --- | --- | --- | --- | --- | --- | --- | --- | --- | --- | --- | --- | --- | --- | --- | --- | --- | --- | --- | --- | --- | --- |
| **Acronym** | **(Location A)** | | | | **(Location B)** | | | |  | **(Location A)** | | | | **(Location B)** | | | |  | **(Location A)** | | | | **(Location B)** | | | |
|  | R | Q | RL | MDL | R | Q | RL | MDL |  | R | Q | RL | MDL | R | Q | RL | MDL |  | R | Q | RL | MDL | R | Q | RL | MDL |
| 3:3 FTCA | 3.1 | U *- cn | 9.3 | 3.1 | 1 | U | 3.1 | 1 |  | ND | U | 37.0 | 11.6 | ND | U | 7.9 | 2.5 |  | ND | U | 11.4 | 3.61 | ND | U | 8.9 | 2.83 |
| 5:3 FTCA | 3.1 | U *- cn | 9.3 | 3.1 | 70 | N/A | 3.1 | 1 |  | ND | U | 37.0 | 11.6 | 24.0 | N/A | 7.9 | 2.5 |  | 117.2 | N/A | 11.4 | 3.61 | 193.2 | N/A | 8.9 | 2.83 |
| 7:3 FTCA | 3.1 | U *- cn | 9.3 | 3.1 | 6.1 | *- cn | 3.1 | 1 |  | ND | U | 37.0 | 11.6 | ND | U | 7.9 | 2.5 |  | 5.9 | N/A | 3.2 | 1.01 | 25.9 | N/A | 2.5 | 0.79 |
| 4:2 FTS | 9.3 | U | 31 | 9.3 | 3.1 | U | 10 | 3.1 |  | ND | U | 6.1 | 1.9 | ND | U | 1.3 | 0.4 |  | ND | U | 0.3 | 0.10 | ND | U | 0.2 | 0.08 |
| 6:2 FTS | 9.3 | U | 31 | 9.3 | 3.1 | U | 10 | 3.1 |  | ND | U | 10.7 | 3.4 | ND | U | 2.3 | 0.7 |  | ND | U | 0.3 | 0.12 | ND | U | 0.2 | 0.09 |
| 8:2 FTS | 9.3 | U | 46 | 9.3 | 3.1 | U | 16 | 3.1 |  | ND | U | 8.4 | 2.7 | ND | U | 1.8 | 0.6 |  | ND | U | 0.0 | 0.02 | ND | U | 0.0 | 0.02 |
| PFMPA | 3.1 | U | 9.3 | 3.1 | 1 | U | 3.1 | 1 |  | ND | U | 7.7 | 2.4 | ND | U | 1.6 | 0.5 |  | ND | U | 1.7 | 0.53 | ND | U | 1.3 | 0.42 |
| PFMBA | 3.1 | U *- cn | 9.3 | 3.1 | 1 | U | 3.1 | 1 |  | ND | U | 3.8 | 1.2 | ND | U | 0.8 | 0.3 |  | ND | U | 3.3 | 1.04 | ND | U | 2.5 | 0.82 |
| NFDHA | 3.1 | U *- cn | 9.3 | 3.1 | 1 | U | 3.1 | 1 |  | ND | U | 6.9 | 2.2 | ND | U | 1.4 | 0.5 |  | ND | U | 0.6 | 0.19 | ND | U | 0.4 | 0.15 |
| HFPODA | 15 | U | 31 | 15 | 5.2 | U | 10 | 5.2 |  | ND | U | 9.2 | 2.9 | ND | U | 1.9 | 0.6 |  | ND | U | 12.3 | 3.90 | ND | U | 9.6 | 3.05 |
| DONA | 3.1 | U | 46 | 3.1 | 1 | U | 16 | 1 |  | ND | U | 7.7 | 2.4 | ND | U | 1.6 | 0.5 |  | ND | U | 0.8 | 0.28 | ND | U | 0.6 | 0.22 |
| NMeFOSAA | 3.1 | U | 31 | 3.1 | 5.2 | J | 10 | 1 |  | ND | U | 5.3 | 1.7 | ND | U | 1.1 | 0.4 |  | 5.2 | N/A | 0.3 | 0.10 | 16.2 | N/A | 0.2 | 0.08 |
| NEtFOSAA | 3.1 | U | 31 | 3.1 | 7.8 | J | 10 | 1 |  | ND | U | 3.8 | 1.2 | ND | U | 0.8 | 0.3 |  | 3.5 | N/A | 0.4 | 0.15 | 6.92 | N/A | 0.3 | 0.12 |
| PFOSA | 3.1 | U | 9.3 | 3.1 | 1 | U | 3.1 | 1 |  | ND | U | 3.0 | 1.0 | ND | U | 0.6 | 0.2 |  | ND | U | 0.3 | 0.10 | ND | U | 0.2 | 0.08 |
| NMeFOSA | 7.7 | U | 31 | 7.7 | 2.6 | U *+ | 10 | 2.6 |  | ND | U | 3.0 | 1.0 | ND | U | 0.6 | 0.2 |  | ND | U | 2.6 | 0.84 | ND | U | 2.0 | 0.66 |
| NEtFOSA | 7.7 | U | 31 | 7.7 | 2.6 | U | 10 | 2.6 |  | ND | U | 3.0 | 1.0 | ND | U | 0.6 | 0.2 |  | ND | U | 2.9 | 0.92 | ND | U | 2.2 | 0.72 |
| NMeFOSE | 7.7 | U | 31 | 7.7 | 9.5 | J | 10 | 2.6 |  | ND | U | 30.8 | 9.7 | ND | U | 6.6 | 2.1 |  | 52.9 | N/A | 8.1 | 2.58 | 100.8 | N/A | 6.4 | 2.02 |
| NEtFOSE | 7.7 | U | 31 | 7.7 | 2.6 | J | 10 | 2.6 |  | ND | U | 30.8 | 9.7 | ND | U | 6.6 | 2.1 |  | ND | U | 6.2 | 3.13 | ND | U | 4.8 | 2.45 |
| PFBA | 12 | U | 31 | 12 | 4.2 | U | 10 | 4.2 |  | ND | U | 10.0 | 3.2 | ND | U | 2.1 | 0.7 |  | ND | U | 20.3 | 10.19 | ND | U | 15.9 | 7.98 |
| PFPeA | 3.1 | U | 9.3 | 3.1 | 1 | U | 3.1 | 1 |  | ND | U | 6.1 | 1.9 | ND | U | 1.3 | 0.4 |  | ND | U | 5.5 | 1.74 | ND | U | 4.3 | 1.36 |
| PFHxA | 3.1 | U | 9.3 | 3.1 | 1 | U | 3.1 | 1 |  | ND | U | 9.2 | 2.9 | ND | U | 1.9 | 0.6 |  | ND | U | 9.1 | 2.87 | ND | U | 7.1 | 2.24 |
| PFHpA | 3.1 | U | 9.3 | 3.1 | 1 | U | 3.1 | 1 |  | ND | U | 4.6 | 1.5 | ND | U | 0.9 | 0.3 |  | ND | U | 3.7 | 1.19 | ND | U | 2.9 | 0.93 |
| PFOA | 3.1 | U | 9.3 | 3.1 | 1 | U | 3.1 | 1 |  | ND | U | 5.3 | 1.7 | ND | U | 1.1 | 0.4 |  | ND | U | 3.9 | 1.26 | ND | U | 3.1 | 0.98 |
| PFNA | 3.1 | U | 9.3 | 3.1 | 1 | U | 3.1 | 1 |  | ND | U | 3.0 | 1.0 | ND | U | 0.6 | 0.2 |  | ND | U | 2.2 | 0.69 | ND | U | 1.7 | 0.54 |
| PFDA | 3.1 | U | 9.3 | 3.1 | 2.8 | J | 3.1 | 1 |  | ND | U | 3.8 | 1.2 | ND | U | 0.8 | 0.3 |  | ND | U | 2.2 | 0.72 | ND | U | 1.7 | 0.57 |
| PFUA | 3.1 | U | 9.3 | 3.1 | 2 | J | 3.1 | 1 |  | ND | U | 4.6 | 1.5 | ND | U | 0.9 | 0.3 |  | 1.1 | N/A | 0.2 | 0.07 | 2.5 | N/A | 0.1 | 0.05 |
| PFDoA | 3.1 | U | 9.3 | 3.1 | 2.9 | J | 3.1 | 1 |  | ND | U | 6.1 | 1.9 | ND | U | 1.3 | 0.4 |  | 1.3 | N/A | 0.5 | 0.17 | 2.5 | N/A | 0.4 | 0.13 |
| PFTrDA | 3.1 | U | 9.3 | 3.1 | 1 | U | 3.1 | 1 |  | ND | U | 6.1 | 1.9 | ND | U | 1.3 | 0.4 |  | ND | U | 0.7 | 0.22 | ND | U | 0.5 | 0.18 |
| PFTeA | 3.1 | U | 9.3 | 3.1 | 1.1 | J | 3.1 | 1 |  | ND | U | 13.1 | 4.1 | ND | U | 2.8 | 0.9 |  | ND | U | 2.8 | 0.89 | ND | U | 2.2 | 0.69 |
| PFBS | 6.2 | U | 31 | 6.2 | 2.1 | U | 10 | 2.1 |  | 4.3 | N/A | 3.8 | 1.2 | ND | U | 0.8 | 0.3 |  | ND | U | 7.9 | 2.51 | ND | U | 6.2 | 1.97 |
| PFPS | 3.1 | U | 9.3 | 3.1 | 1 | U | 3.1 | 1 |  | ND | U | 5.3 | 1.7 | ND | U | 1.1 | 0.4 |  | ND | U | 1.6 | 0.53 | ND | U | 1.3 | 0.41 |
| PFHxS | 3.1 | U | 9.3 | 3.1 | 3 | J | 3.1 | 1 |  | 1.8 | J | 8.4 | 2.7 | ND | U | 1.8 | 0.6 |  | ND | U | 3.4 | 1.08 | ND | U | 2.6 | 0.84 |
| PFHpS | 3.1 | U | 9.3 | 3.1 | 1 | U | 3.1 | 1 |  | ND | U | 6.1 | 1.9 | ND | U | 1.3 | 0.4 |  | ND | U | 1.7 | 0.56 | ND | U | 1.3 | 0.43 |
| PFOS | 3.1 | U | 9.3 | 3.1 | 2.6 | J | 3.1 | 1 |  | ND | U | 3.8 | 1.2 | 66.6 | N/A | 0.8 | 0.3 |  | ND | U | 11.7 | 3.69 | ND | U | 9.1 | 2.89 |
| PFNS | 3.1 | U | 9.3 | 3.1 | 1 | U | 3.1 | 1 |  | ND | U | 11.5 | 3.6 | ND | U | 2.4 | 0.8 |  | ND | U | 0.4 | 0.15 | ND | U | 0.3 | 0.11 |
| PFDS | 3.1 | U | 9.3 | 3.1 | 1 | J | 3.1 | 1 |  | ND | U | 6.9 | 2.2 | ND | U | 1.4 | 0.5 |  | ND | U | 0.2 | 0.08 | ND | U | 0.2 | 0.07 |
| PFDoS | 3.1 | U | 31 | 3.1 | 1.2 | J | 10 | 1 |  | ND | U | 15.4 | 4.8 | ND | U | 3.3 | 1.0 |  | ND | U | 0.1 | 0.04 | ND | U | 0.1 | 0.03 |
| PFEESA/PES | 3.1 | U | 9.3 | 3.1 | 1 | U | 3.1 | 1 |  | ND | U | 2.3 | 0.7 | ND | U | 0.4 | 0.2 |  | ND | U | 12.7 | 4.01 | ND | U | 9.9 | 3.14 |
| 9Cl-PF3ONS | 3.1 | U | 31 | 3.1 | 1 | U | 10 | 1 |  | ND | U | 4.6 | 1.5 | ND | U | 0.9 | 0.3 |  | ND | U | 0.8 | 0.28 | ND | U | 0.6 | 0.22 |
| 11Cl-PF3OUdS | 3.1 | U | 9.3 | 3.1 | 1 | U | 3.1 | 1 |  | ND | U | 3.8 | 1.2 | ND | U | 0.8 | 0.3 |  | ND | U | 0.8 | 0.28 | ND | U | 0.6 | 0.22 |

Site 6

| **Lab** | **Lab 1** | | | | | | | |  | **Lab 2** | | | | | | | |  | **Lab 3** | | | | | | | |
| --- | --- | --- | --- | --- | --- | --- | --- | --- | --- | --- | --- | --- | --- | --- | --- | --- | --- | --- | --- | --- | --- | --- | --- | --- | --- | --- |
| **Acronym** | **(Location A)** | | | | **(Location B)** | | | |  | **(Location A)** | | | | **(Location B)** | | | |  | **(Location A)** | | | | **(Location B)** | | | |
|  | R | Q | RL | MDL | R | Q | RL | MDL |  | R | Q | RL | MDL | R | Q | RL | MDL |  | R | Q | RL | MDL | R | Q | RL | MDL |
| 3:3 FTCA | 4.4 | U | 13 | 4.4 | 0.91 | U | 2.7 | 0.91 |  | ND | U | 40.8 | 12.8 | ND | U | 7.5 | 2.4 |  | ND | U | 12.6 | 3.97 | ND | U | 7.9 | 2.51 |
| 5:3 FTCA | 33 | N/A | 13 | 4.4 | 100 | N/A | 2.7 | 0.91 |  | ND | U | 40.8 | 12.8 | 28.3 | N/A | 7.5 | 2.4 |  | 163.9 | N/A | 12.6 | 3.97 | 507.7 | N/A | 7.9 | 2.51 |
| 7:3 FTCA | 4.4 | U *- cn | 13 | 4.4 | 6.6 | *- cn | 2.7 | 0.91 |  | ND | U | 40.8 | 12.8 | ND | U | 7.5 | 2.4 |  | 12.1 | N/A | 3.5 | 1.11 | 45.0 | N/A | 2.2 | 0.70 |
| 4:2 FTS | 13 | U | 44 | 13 | 2.7 | U | 9.1 | 2.7 |  | ND | U | 6.8 | 2.1 | ND | U | 1.2 | 0.4 |  | ND | U | 0.3 | 0.11 | ND | U | 0.2 | 0.07 |
| 6:2 FTS | 13 | U | 44 | 13 | 2.7 | U | 9.1 | 2.7 |  | ND | U | 11.9 | 3.7 | ND | U | 2.1 | 0.7 |  | ND | U | 0.4 | 0.13 | ND | U | 0.2 | 0.08 |
| 8:2 FTS | 13 | U | 66 | 13 | 2.7 | U | 14 | 2.7 |  | ND | U | 9.3 | 2.9 | ND | U | 1.7 | 0.5 |  | 0.51 | N/A | 0.0 | 0.02 | 0.71 | N/A | 0.0 | 0.01 |
| PFMPA | 4.4 | U | 13 | 4.4 | 0.91 | U | 2.7 | 0.91 |  | ND | U | 8.5 | 2.7 | ND | U | 1.5 | 0.5 |  | ND | U | 1.8 | 0.59 | ND | U | 1.1 | 0.37 |
| PFMBA | 4.4 | U | 13 | 4.4 | 0.91 | U | 2.7 | 0.91 |  | ND | U | 4.2 | 1.3 | ND | U | 0.7 | 0.2 |  | ND | U | 3.6 | 1.15 | ND | U | 2.3 | 0.72 |
| NFDHA | 4.4 | U | 13 | 4.4 | 0.91 | U | 2.7 | 0.91 |  | ND | U | 7.6 | 2.4 | ND | U | 1.4 | 0.4 |  | ND | U | 0.6 | 0.21 | ND | U | 0.4 | 0.14 |
| HFPODA | 22 | U | 44 | 22 | 4.5 | U | 9.1 | 4.5 |  | ND | U | 10.2 | 3.2 | ND | U | 1.8 | 0.6 |  | ND | U | 13.6 | 4.28 | ND | U | 8.6 | 2.71 |
| DONA | 4.4 | U | 66 | 4.4 | 0.91 | U | 14 | 0.91 |  | ND | U | 8.5 | 2.7 | ND | U | 1.5 | 0.5 |  | ND | U | 0.9 | 0.31 | ND | U | 0.6 | 0.19 |
| NMeFOSAA | 4.4 | U | 44 | 4.4 | 4.4 | J | 9.1 | 0.91 |  | ND | U | 5.9 | 1.9 | ND | U | 1.0 | 0.3 |  | 2.3 | N/A | 0.3 | 0.11 | 5.4 | N/A | 0.2 | 0.07 |
| NEtFOSAA | 4.4 | U | 44 | 4.4 | 5.8 | J | 9.1 | 0.91 |  | ND | U | 4.2 | 1.3 | ND | U | 0.7 | 0.2 |  | 2.0 | N/A | 0.5 | 0.17 | 4.80 | N/A | 0.3 | 0.11 |
| PFOSA | 4.4 | U | 13 | 4.4 | 0.98 | J | 2.7 | 0.91 |  | ND | U | 3.4 | 1.1 | ND | U | 0.6 | 0.2 |  | ND | U | 0.3 | 0.11 | ND | U | 0.2 | 0.07 |
| NMeFOSA | 11 | U *+ | 44 | 11 | 2.3 | U *+ | 9.1 | 2.3 |  | ND | U | 3.4 | 1.1 | ND | U | 0.6 | 0.2 |  | ND | U | 2.9 | 0.92 | ND | U | 1.8 | 0.58 |
| NEtFOSA | 11 | U | 44 | 11 | 2.3 | U | 9.1 | 2.3 |  | ND | U | 3.4 | 1.1 | ND | U | 0.6 | 0.2 |  | ND | U | 3.2 | 1.02 | ND | U | 2.0 | 0.64 |
| NMeFOSE | 11 | U | 44 | 11 | 7.7 | J | 9.1 | 2.3 |  | ND | U | 34.0 | 10.7 | ND | U | 6.2 | 2.0 |  | ND | U | 9.0 | 2.83 | 16.9 | N/A | 5.7 | 1.79 |
| NEtFOSE | 11 | U | 44 | 11 | 2.3 | U | 9.1 | 2.3 |  | ND | U | 34.0 | 10.7 | ND | U | 6.2 | 2.0 |  | ND | U | 6.8 | 3.44 | ND | U | 4.3 | 2.18 |
| PFBA | 18 | U | 44 | 18 | 3.6 | U | 9.1 | 3.6 |  | ND | U | 11.0 | 3.5 | ND | U | 2.0 | 0.6 |  | ND | U | 22.4 | 11.21 | ND | U | 14.1 | 7.09 |
| PFPeA | 4.4 | U | 13 | 4.4 | 0.91 | U | 2.7 | 0.91 |  | ND | U | 6.8 | 2.1 | ND | U | 1.2 | 0.4 |  | ND | U | 6.0 | 1.92 | ND | U | 3.8 | 1.21 |
| PFHxA | 4.4 | U | 13 | 4.4 | 1.8 | J | 2.7 | 0.91 |  | ND | U | 10.2 | 3.2 | ND | U | 1.8 | 0.6 |  | ND | U | 10.0 | 3.15 | ND | U | 6.3 | 2.00 |
| PFHpA | 4.4 | U | 13 | 4.4 | 0.91 | U | 2.7 | 0.91 |  | ND | U | 5.1 | 1.6 | ND | U | 0.9 | 0.3 |  | ND | U | 4.1 | 1.31 | ND | U | 2.6 | 0.83 |
| PFOA | 4.4 | U | 13 | 4.4 | 1.2 | J | 2.7 | 0.91 |  | ND | U | 5.9 | 1.9 | ND | U | 1.0 | 0.3 |  | ND | U | 4.3 | 1.38 | ND | U | 2.7 | 0.87 |
| PFNA | 4.4 | U | 13 | 4.4 | 1.2 | J | 2.7 | 0.91 |  | ND | U | 3.4 | 1.1 | ND | U | 0.6 | 0.2 |  | ND | U | 2.4 | 0.76 | ND | U | 1.5 | 0.48 |
| PFDA | 4.4 | U | 13 | 4.4 | 3.9 | N/A | 2.7 | 0.91 |  | ND | U | 4.2 | 1.3 | 5.5 | N/A | 0.7 | 0.2 |  | ND | U | 2.5 | 0.79 | ND | U | 1.5 | 0.50 |
| PFUA | 4.4 | U | 13 | 4.4 | 2.2 | J | 2.7 | 0.91 |  | ND | U | 5.1 | 1.6 | ND | U | 0.9 | 0.3 |  | ND | U | 0.2 | 0.08 | 2.7 | N/A | 0.1 | 0.05 |
| PFDoA | 4.4 | U | 13 | 4.4 | 3.9 | N/A | 2.7 | 0.91 |  | ND | U | 6.8 | 2.1 | ND | U | 1.2 | 0.4 |  | ND | U | 0.5 | 0.18 | 5.2 | N/A | 0.3 | 0.12 |
| PFTrDA | 4.4 | U | 13 | 4.4 | 0.91 | U | 2.7 | 0.91 |  | ND | U | 6.8 | 2.1 | ND | U | 1.2 | 0.4 |  | ND | U | 0.7 | 0.25 | ND | U | 0.4 | 0.16 |
| PFTeA | 4.4 | U | 13 | 4.4 | 1.6 | J | 2.7 | 0.91 |  | ND | U | 14.4 | 4.5 | ND | U | 2.6 | 0.8 |  | ND | U | 3.1 | 0.98 | ND | U | 1.9 | 0.62 |
| PFBS | 8.8 | U | 44 | 8.8 | 1.8 | U | 9.1 | 1.8 |  | ND | U | 4.2 | 1.3 | ND | U | 0.7 | 0.2 |  | ND | U | 8.7 | 2.76 | ND | U | 5.5 | 1.75 |
| PFPS | 4.4 | U | 13 | 4.4 | 0.91 | U | 2.7 | 0.91 |  | ND | U | 5.9 | 1.9 | ND | U | 1.0 | 0.3 |  | ND | U | 1.8 | 0.58 | ND | U | 1.1 | 0.37 |
| PFHxS | 4.4 | U | 13 | 4.4 | 0.91 | U | 2.7 | 0.91 |  | ND | U | 9.3 | 2.9 | ND | U | 1.7 | 0.5 |  | ND | U | 3.7 | 1.18 | ND | U | 2.3 | 0.75 |
| PFHpS | 4.4 | U | 13 | 4.4 | 0.91 | U | 2.7 | 0.91 |  | ND | U | 6.8 | 2.1 | ND | U | 1.2 | 0.4 |  | ND | U | 1.9 | 0.61 | ND | U | 1.2 | 0.39 |
| PFOS | 4.5 | J | 13 | 4.4 | 6.3 | N/A | 2.7 | 0.91 |  | ND | U | 4.2 | 1.3 | 15.0 | N/A | 0.7 | 0.2 |  | ND | U | 12.8 | 4.05 | ND | U | 8.1 | 2.57 |
| PFNS | 4.4 | U | 13 | 4.4 | 0.91 | U | 2.7 | 0.91 |  | ND | U | 12.7 | 4.0 | ND | U | 2.3 | 0.7 |  | ND | U | 0.5 | 0.16 | ND | U | 0.3 | 0.10 |
| PFDS | 4.4 | U | 13 | 4.4 | 2 | J | 2.7 | 0.91 |  | ND | U | 7.6 | 2.4 | ND | U | 1.4 | 0.4 |  | ND | U | 0.2 | 0.09 | ND | U | 0.1 | 0.06 |
| PFDoS | 5.2 | J | 44 | 4.4 | 0.91 | U | 9.1 | 0.91 |  | ND | U | 17.0 | 5.4 | ND | U | 3.1 | 1.0 |  | ND | U | 0.1 | 0.05 | ND | U | 0.0 | 0.03 |
| PFEESA/PES | 4.4 | U | 13 | 4.4 | 0.91 | U | 2.7 | 0.91 |  | ND | U | 2.5 | 0.8 | ND | U | 0.4 | 0.1 |  | ND | U | 14.0 | 4.40 | ND | U | 8.8 | 2.79 |
| 9Cl-PF3ONS | 4.4 | U | 44 | 4.4 | 0.91 | U | 9.1 | 0.91 |  | ND | U | 5.1 | 1.6 | ND | U | 0.9 | 0.3 |  | ND | U | 0.9 | 0.31 | ND | U | 0.6 | 0.19 |
| 11Cl-PF3OUdS | 4.4 | U | 13 | 4.4 | 0.91 | U | 2.7 | 0.91 |  | ND | U | 4.2 | 1.3 | ND | U | 0.7 | 0.2 |  | ND | U | 0.9 | 0.31 | ND | U | 0.6 | 0.19 |

Site 7

| **Lab** | **Lab 1** | | | | | | | |  | **Lab 2** | | | | | | | |  | **Lab 3** | | | | | | | |
| --- | --- | --- | --- | --- | --- | --- | --- | --- | --- | --- | --- | --- | --- | --- | --- | --- | --- | --- | --- | --- | --- | --- | --- | --- | --- | --- |
| **Acronym** | **(Location A)** | | | | **(Location B)** | | | |  | **(Location A)** | | | | **(Location B)** | | | |  | **(Location A)** | | | | **(Location B)** | | | |
|  | R | Q | RL | MDL | R | Q | RL | MDL |  | R | Q | RL | MDL | R | Q | RL | MDL |  | R | Q | RL | MDL | R | Q | RL | MDL |
| 3:3 FTCA | 4 | U | 12 | 4 | 1 | U | 3.1 | 1 |  | ND | U | 34.9 | 11.0 | ND | U | 7.6 | 2.4 |  | ND | U | 14.5 | 4.56 | ND | U | 9.2 | 2.90 |
| 5:3 FTCA | 21 | N/A | 12 | 4 | 27 | N/A | 3.1 | 1 |  | ND | U | 34.9 | 11.0 | ND | U | 7.6 | 2.4 |  | 86.2 | N/A | 14.5 | 4.56 | 160.8 | N/A | 9.2 | 2.90 |
| 7:3 FTCA | 8.2 | J *- cn | 12 | 4 | 5.8 | *- cn | 3.1 | 1 |  | ND | U | 34.9 | 11.0 | ND | U | 7.6 | 2.4 |  | 60.8 | N/A | 4.0 | 1.28 | 55.9 | N/A | 2.5 | 0.81 |
| 4:2 FTS | 12 | U | 40 | 12 | 3.1 | U | 10 | 3.1 |  | ND | U | 5.8 | 1.8 | ND | U | 1.2 | 0.4 |  | ND | U | 0.4 | 0.13 | ND | U | 0.2 | 0.08 |
| 6:2 FTS | 12 | U | 40 | 12 | 3.1 | U | 10 | 3.1 |  | ND | U | 10.2 | 3.2 | 98.1 | N/A | 2.2 | 0.7 |  | ND | U | 0.4 | 0.15 | ND | U | 0.3 | 0.09 |
| 8:2 FTS | 12 | U | 60 | 12 | 3.1 | U | 15 | 3.1 |  | ND | U | 8.0 | 2.5 | ND | U | 1.7 | 0.6 |  | 0.59 | N/A | 0.0 | 0.03 | 1.07 | N/A | 0.0 | 0.02 |
| PFMPA | 4 | U | 12 | 4 | 1 | U | 3.1 | 1 |  | ND | U | 7.2 | 2.3 | ND | U | 1.5 | 0.5 |  | ND | U | 2.1 | 0.68 | ND | U | 1.3 | 0.43 |
| PFMBA | 4 | U | 12 | 4 | 1 | U | 3.1 | 1 |  | ND | U | 3.6 | 1.1 | ND | U | 0.7 | 0.3 |  | ND | U | 4.1 | 1.32 | ND | U | 2.6 | 0.84 |
| NFDHA | 4 | U | 12 | 4 | 1 | U | 3.1 | 1 |  | ND | U | 6.5 | 2.1 | ND | U | 1.4 | 0.5 |  | ND | U | 0.7 | 0.25 | ND | U | 0.4 | 0.16 |
| HFPODA | 20 | U | 40 | 20 | 5.1 | U | 10 | 5.1 |  | ND | U | 8.7 | 2.8 | ND | U | 1.9 | 0.6 |  | ND | U | 15.6 | 4.92 | ND | U | 9.9 | 3.13 |
| DONA | 4 | U | 60 | 4 | 1 | U | 15 | 1 |  | ND | U | 7.2 | 2.3 | ND | U | 1.5 | 0.5 |  | ND | U | 1.1 | 0.35 | ND | U | 0.7 | 0.22 |
| NMeFOSAA | 8 | J | 40 | 4 | 9.2 | J | 10 | 1 |  | ND | U | 5.1 | 1.6 | ND | U | 1.1 | 0.4 |  | 6.2 | N/A | 0.3 | 0.12 | 10.8 | N/A | 0.2 | 0.08 |
| NEtFOSAA | 5.6 | J | 40 | 4 | 5.3 | J | 10 | 1 |  | ND | U | 3.6 | 1.1 | ND | U | 0.7 | 0.3 |  | 3.2 | N/A | 0.6 | 0.19 | 4.87 | N/A | 0.3 | 0.12 |
| PFOSA | 4 | U | 12 | 4 | 1 | U | 3.1 | 1 |  | ND | U | 2.9 | 0.9 | ND | U | 0.6 | 0.2 |  | ND | U | 0.3 | 0.12 | ND | U | 0.2 | 0.08 |
| NMeFOSA | 10 | U *+ | 40 | 10 | 2.5 | U *+ | 10 | 2.5 |  | ND | U | 2.9 | 0.9 | ND | U | 0.6 | 0.2 |  | ND | U | 3.3 | 1.06 | ND | U | 2.1 | 0.68 |
| NEtFOSA | 10 | U | 40 | 10 | 2.5 | U | 10 | 2.5 |  | ND | U | 2.9 | 0.9 | ND | U | 0.6 | 0.2 |  | ND | U | 3.7 | 1.17 | ND | U | 2.3 | 0.74 |
| NMeFOSE | 10 | U | 40 | 10 | 3.3 | J | 10 | 2.5 |  | ND | U | 29.1 | 9.2 | ND | U | 6.3 | 2.0 |  | 49.1 | N/A | 10.3 | 3.26 | 30.9 | N/A | 6.5 | 2.07 |
| NEtFOSE | 10 | U | 40 | 10 | 2.5 | U | 10 | 2.5 |  | ND | U | 29.1 | 9.2 | ND | U | 6.3 | 2.0 |  | ND | U | 7.9 | 3.95 | ND | U | 5.0 | 2.52 |
| PFBA | 16 | U | 40 | 16 | 4.1 | U | 10 | 4.1 |  | ND | U | 9.4 | 3.0 | ND | U | 2.0 | 0.7 |  | ND | U | 25.7 | 12.88 | ND | U | 16.4 | 8.20 |
| PFPeA | 4 | U | 12 | 4 | 1 | U | 3.1 | 1 |  | ND | U | 5.8 | 1.8 | ND | U | 1.2 | 0.4 |  | ND | U | 7.0 | 2.20 | ND | U | 4.4 | 1.40 |
| PFHxA | 4 | U | 12 | 4 | 1.3 | J | 3.1 | 1 |  | ND | U | 8.7 | 2.8 | ND | U | 1.9 | 0.6 |  | ND | U | 11.5 | 3.62 | ND | U | 7.3 | 2.31 |
| PFHpA | 4 | U | 12 | 4 | 1 | U | 3.1 | 1 |  | ND | U | 4.3 | 1.4 | ND | U | 0.9 | 0.3 |  | ND | U | 4.7 | 1.50 | ND | U | 3.0 | 0.96 |
| PFOA | 4 | U | 12 | 4 | 1.4 | J | 3.1 | 1 |  | ND | U | 5.1 | 1.6 | 42.9 | N/A | 1.1 | 0.4 |  | ND | U | 5.0 | 1.59 | ND | U | 3.2 | 1.01 |
| PFNA | 4 | U | 12 | 4 | 1 | U | 3.1 | 1 |  | ND | U | 2.9 | 0.9 | ND | U | 0.6 | 0.2 |  | ND | U | 2.7 | 0.88 | ND | U | 1.7 | 0.56 |
| PFDA | 16 | N/A | 12 | 4 | 8.9 | N/A | 3.1 | 1 |  | ND | U | 3.6 | 1.1 | ND | U | 0.7 | 0.3 |  | ND | U | 2.9 | 0.91 | ND | U | 1.8 | 0.58 |
| PFUA | 4 | U | 12 | 4 | 1.3 | J | 3.1 | 1 |  | ND | U | 4.3 | 1.4 | ND | U | 0.9 | 0.3 |  | ND | U | 0.2 | 0.09 | ND | U | 0.1 | 0.06 |
| PFDoA | 6.1 | J | 12 | 4 | 3.5 | N/A | 3.1 | 1 |  | ND | U | 5.8 | 1.8 | ND | U | 1.2 | 0.4 |  | 3.3 | N/A | 0.6 | 0.21 | 5.5 | N/A | 0.4 | 0.13 |
| PFTrDA | 4 | U | 12 | 4 | 1 | U | 3.1 | 1 |  | ND | U | 5.8 | 1.8 | ND | U | 1.2 | 0.4 |  | ND | U | 0.9 | 0.28 | ND | U | 0.5 | 0.18 |
| PFTeA | 4 | U | 12 | 4 | 1.1 | J | 3.1 | 1 |  | ND | U | 12.3 | 3.9 | ND | U | 2.7 | 0.9 |  | ND | U | 3.5 | 1.12 | ND | U | 2.2 | 0.71 |
| PFBS | 8 | U | 40 | 8 | 2 | U | 10 | 2 |  | 9.8 | N/A | 3.6 | 1.1 | ND | U | 0.7 | 0.3 |  | ND | U | 10.0 | 3.18 | ND | U | 6.4 | 2.02 |
| PFPS | 4 | U | 12 | 4 | 1 | U | 3.1 | 1 |  | ND | U | 5.1 | 1.6 | ND | U | 1.1 | 0.4 |  | ND | U | 2.1 | 0.67 | ND | U | 1.3 | 0.42 |
| PFHxS | 4 | U | 12 | 4 | 1 | U | 3.1 | 1 |  | ND | U | 8.0 | 2.5 | ND | U | 1.7 | 0.6 |  | ND | U | 4.3 | 1.36 | ND | U | 2.7 | 0.87 |
| PFHpS | 4 | U | 12 | 4 | 1 | U | 3.1 | 1 |  | ND | U | 5.8 | 1.8 | ND | U | 1.2 | 0.4 |  | ND | U | 2.2 | 0.70 | ND | U | 1.4 | 0.45 |
| PFOS | 11 | J | 12 | 4 | 9.5 | N/A | 3.1 | 1 |  | 145.6 | N/A | 3.6 | 1.1 | 113.4 | N/A | 0.7 | 0.3 |  | ND | U | 14.8 | 4.66 | ND | U | 9.4 | 2.97 |
| PFNS | 4 | U | 12 | 4 | 1 | U | 3.1 | 1 |  | ND | U | 10.9 | 3.4 | ND | U | 2.3 | 0.8 |  | ND | U | 0.5 | 0.18 | ND | U | 0.3 | 0.12 |
| PFDS | 4 | U | 12 | 4 | 1.5 | J I | 3.1 | 1 |  | ND | U | 6.5 | 2.1 | ND | U | 1.4 | 0.5 |  | ND | U | 0.3 | 0.11 | ND | U | 0.2 | 0.07 |
| PFDoS | 4 | U | 40 | 4 | 2.3 | J | 10 | 1 |  | ND | U | 14.5 | 4.6 | ND | U | 3.1 | 1.0 |  | ND | U | 0.1 | 0.05 | ND | U | 0.1 | 0.03 |
| PFEESA/PES | 4 | U | 12 | 4 | 1 | U | 3.1 | 1 |  | ND | U | 2.1 | 0.7 | ND | U | 0.4 | 0.2 |  | ND | U | 16.0 | 5.06 | ND | U | 10.2 | 3.22 |
| 9Cl-PF3ONS | 4 | U | 40 | 4 | 1 | U | 10 | 1 |  | ND | U | 4.3 | 1.4 | ND | U | 0.9 | 0.3 |  | ND | U | 1.1 | 0.35 | ND | U | 0.7 | 0.22 |
| 11Cl-PF3OUdS | 4 | U | 12 | 4 | 1 | U | 3.1 | 1 |  | ND | U | 3.6 | 1.1 | ND | U | 0.7 | 0.3 |  | ND | U | 1.1 | 0.35 | ND | U | 0.7 | 0.22 |

Site 8

| **Lab** | **Lab 1** | | | | | | | |  | **Lab 2** | | | | | | | |  | **Lab 3** | | | | | | | |
| --- | --- | --- | --- | --- | --- | --- | --- | --- | --- | --- | --- | --- | --- | --- | --- | --- | --- | --- | --- | --- | --- | --- | --- | --- | --- | --- |
| **Acronym** | **(Location A)** | | | | **(Location E)** | | | |  | **(Location A)** | | | | **(Location E)** | | | |  | **(Location A)** | | | | **(Location E)** | | | |
|  | R | Q | RL | MDL | R | Q | RL | MDL |  | R | Q | RL | MDL | R | Q | RL | MDL |  | R | Q | RL | MDL | R | Q | RL | MDL |
| 3:3 FTCA | 5.3 | U *- cn | 16 | 5.3 | 0.2 | U *- cn | 0.6 | 0.2 |  | ND | U | 67.5 | 21.2 | ND | U | 1.5 | 0.5 |  | ND | U | 11.0 | 3.49 | ND | U | 1.6 | 0.52 |
| 5:3 FTCA | 20 | N/A | 16 | 5.3 | 0.2 | U | 0.6 | 0.2 |  | ND | U | 67.5 | 21.2 | ND | U | 1.5 | 0.5 |  | 362.3 | N/A | 11.0 | 3.49 | ND | U | 1.6 | 0.52 |
| 7:3 FTCA | 5.3 | U *- cn | 16 | 5.3 | 0.2 | U *- cn | 0.6 | 0.2 |  | ND | U | 67.5 | 21.2 | ND | U | 1.5 | 0.5 |  | 56.9 | N/A | 3.1 | 0.98 | ND | U | 0.4 | 0.15 |
| 4:2 FTS | 16 | U | 53 | 16 | 0.6 | U | 2 | 0.6 |  | ND | U | 11.2 | 3.5 | ND | U | 0.2 | 0.1 |  | ND | U | 0.3 | 0.10 | ND | U | 0.0 | 0.02 |
| 6:2 FTS | 16 | U | 53 | 16 | 0.6 | U | 2 | 0.6 |  | ND | U | 19.6 | 6.2 | ND | U | 0.4 | 0.1 |  | 2.37 | N/A | 0.3 | 0.11 | ND | U | 0.0 | 0.02 |
| 8:2 FTS | 16 | U | 80 | 16 | 0.6 | U | 3 | 0.6 |  | ND | U | 15.4 | 4.9 | ND | U | 0.3 | 0.1 |  | ND | U | 0.0 | 0.02 | ND | U | 0.0 | 0.00 |
| PFMPA | 5.3 | U | 16 | 5.3 | 0.2 | U | 0.6 | 0.2 |  | ND | U | 14.0 | 4.4 | ND | U | 0.3 | 0.1 |  | ND | U | 1.6 | 0.52 | ND | U | 0.2 | 0.08 |
| PFMBA | 5.3 | U | 16 | 5.3 | 0.2 | U | 0.6 | 0.2 |  | ND | U | 7.0 | 2.2 | ND | U | 0.1 | 0.1 |  | ND | U | 3.2 | 1.01 | ND | U | 0.4 | 0.15 |
| NFDHA | 5.3 | U | 16 | 5.3 | 0.2 | U | 0.6 | 0.2 |  | ND | U | 12.6 | 4.0 | ND | U | 0.2 | 0.1 |  | ND | U | 0.5 | 0.19 | ND | U | 0.0 | 0.03 |
| HFPODA | 27 | U | 53 | 27 | 0.99 | U | 2 | 0.99 |  | ND | U | 16.8 | 5.3 | ND | U | 0.3 | 0.1 |  | ND | U | 11.9 | 3.77 | ND | U | 1.7 | 0.56 |
| DONA | 5.3 | U | 80 | 5.3 | 0.2 | U | 3 | 0.2 |  | ND | U | 14.0 | 4.4 | ND | U | 0.3 | 0.1 |  | ND | U | 0.8 | 0.27 | ND | U | 0.1 | 0.04 |
| NMeFOSAA | 5.3 | U | 53 | 5.3 | 0.2 | U | 2 | 0.2 |  | ND | U | 9.8 | 3.1 | ND | U | 0.2 | 0.1 |  | 10.1 | N/A | 0.2 | 0.09 | ND | U | 0.0 | 0.01 |
| NEtFOSAA | 5.3 | U | 53 | 5.3 | 0.2 | U | 2 | 0.2 |  | ND | U | 7.0 | 2.2 | ND | U | 0.1 | 0.1 |  | 5.3 | N/A | 0.4 | 0.15 | ND | U | 0.0 | 0.02 |
| PFOSA | 5.3 | U | 16 | 5.3 | 0.2 | U | 0.6 | 0.2 |  | ND | U | 5.6 | 1.8 | ND | U | 0.1 | 0.0 |  | ND | U | 0.2 | 0.09 | ND | U | 0.0 | 0.01 |
| NMeFOSA | 13 | U *+ | 53 | 13 | 0.5 | U *+ | 2 | 0.5 |  | ND | U | 5.6 | 1.8 | ND | U | 0.1 | 0.0 |  | ND | U | 2.5 | 0.81 | ND | U | 0.3 | 0.12 |
| NEtFOSA | 13 | U | 53 | 13 | 0.5 | U | 2 | 0.5 |  | ND | U | 5.6 | 1.8 | ND | U | 0.1 | 0.0 |  | ND | U | 2.8 | 0.89 | ND | U | 0.4 | 0.13 |
| NMeFOSE | 13 | U | 53 | 13 | 0.5 | U | 2 | 0.5 |  | ND | U | 56.2 | 17.7 | ND | U | 1.2 | 0.4 |  | ND | U | 7.9 | 2.49 | ND | U | 1.1 | 0.37 |
| NEtFOSE | 13 | U | 53 | 13 | 0.5 | U | 2 | 0.5 |  | ND | U | 56.2 | 17.7 | ND | U | 1.2 | 0.4 |  | ND | U | 6.0 | 3.03 | ND | U | 0.9 | 0.45 |
| PFBA | 21 | U | 53 | 21 | 0.79 | U | 2 | 0.79 |  | ND | U | 18.2 | 5.8 | ND | U | 0.4 | 0.1 |  | ND | U | 19.7 | 9.85 | ND | U | 2.9 | 1.47 |
| PFPeA | 5.3 | U | 16 | 5.3 | 0.2 | U | 0.6 | 0.2 |  | ND | U | 11.2 | 3.5 | ND | U | 0.2 | 0.1 |  | ND | U | 5.3 | 1.68 | ND | U | 0.7 | 0.25 |
| PFHxA | 5.3 | U | 16 | 5.3 | 0.2 | U | 0.6 | 0.2 |  | ND | U | 16.8 | 5.3 | ND | U | 0.3 | 0.1 |  | ND | U | 8.8 | 2.77 | ND | U | 1.3 | 0.41 |
| PFHpA | 5.3 | U | 16 | 5.3 | 0.2 | U | 0.6 | 0.2 |  | ND | U | 8.4 | 2.7 | ND | U | 0.1 | 0.1 |  | ND | U | 3.6 | 1.15 | ND | U | 0.5 | 0.17 |
| PFOA | 5.3 | U | 16 | 5.3 | 0.2 | U | 0.6 | 0.2 |  | ND | U | 9.8 | 3.1 | ND | U | 0.2 | 0.1 |  | ND | U | 3.8 | 1.21 | ND | U | 0.5 | 0.18 |
| PFNA | 5.3 | U | 16 | 5.3 | 0.2 | U | 0.6 | 0.2 |  | ND | U | 5.6 | 1.8 | ND | U | 0.1 | 0.0 |  | ND | U | 2.1 | 0.67 | ND | U | 0.3 | 0.10 |
| PFDA | 5.3 | U | 16 | 5.3 | 0.2 | U | 0.6 | 0.2 |  | ND | U | 7.0 | 2.2 | ND | U | 0.1 | 0.1 |  | ND | U | 2.2 | 0.70 | ND | U | 0.3 | 0.10 |
| PFUA | 5.3 | U | 16 | 5.3 | 0.2 | U | 0.6 | 0.2 |  | ND | U | 8.4 | 2.7 | ND | U | 0.1 | 0.1 |  | ND | U | 0.2 | 0.07 | ND | U | 0.0 | 0.01 |
| PFDoA | 5.3 | U | 16 | 5.3 | 0.2 | U | 0.6 | 0.2 |  | ND | U | 11.2 | 3.5 | ND | U | 0.2 | 0.1 |  | 1.1 | N/A | 0.5 | 0.16 | ND | U | 0.0 | 0.02 |
| PFTrDA | 5.3 | U | 16 | 5.3 | 0.2 | U | 0.6 | 0.2 |  | ND | U | 11.2 | 3.5 | ND | U | 0.2 | 0.1 |  | ND | U | 0.6 | 0.22 | ND | U | 0.1 | 0.03 |
| PFTeA | 5.3 | U | 16 | 5.3 | 0.2 | U | 0.6 | 0.2 |  | ND | U | 23.9 | 7.5 | ND | U | 0.5 | 0.2 |  | ND | U | 2.7 | 0.86 | ND | U | 0.4 | 0.13 |
| PFBS | 11 | U | 53 | 11 | 0.4 | U | 2 | 0.4 |  | ND | U | 7.0 | 2.2 | ND | U | 0.1 | 0.1 |  | ND | U | 7.7 | 2.43 | ND | U | 1.1 | 0.36 |
| PFPS | 5.3 | U | 16 | 5.3 | 0.2 | U | 0.6 | 0.2 |  | ND | U | 9.8 | 3.1 | ND | U | 0.2 | 0.1 |  | ND | U | 1.6 | 0.51 | ND | U | 0.2 | 0.08 |
| PFHxS | 5.3 | U | 16 | 5.3 | 0.2 | U | 0.6 | 0.2 |  | ND | U | 15.4 | 4.9 | ND | U | 0.3 | 0.1 |  | ND | U | 3.3 | 1.04 | ND | U | 0.4 | 0.16 |
| PFHpS | 5.3 | U | 16 | 5.3 | 0.2 | U | 0.6 | 0.2 |  | ND | U | 11.2 | 3.5 | ND | U | 0.2 | 0.1 |  | ND | U | 1.7 | 0.54 | ND | U | 0.2 | 0.08 |
| PFOS | 5.9 | J | 16 | 5.3 | 0.2 | U | 0.6 | 0.2 |  | ND | U | 7.0 | 2.2 | ND | U | 0.1 | 0.1 |  | ND | U | 11.3 | 3.56 | ND | U | 1.6 | 0.53 |
| PFNS | 5.3 | U | 16 | 5.3 | 0.2 | U | 0.6 | 0.2 |  | ND | U | 21.1 | 6.6 | ND | U | 0.4 | 0.2 |  | ND | U | 0.4 | 0.14 | ND | U | 0.0 | 0.02 |
| PFDS | 5.3 | U | 16 | 5.3 | 0.2 | U | 0.6 | 0.2 |  | ND | U | 12.6 | 4.0 | ND | U | 0.2 | 0.1 |  | ND | U | 0.2 | 0.08 | ND | U | 0.0 | 0.01 |
| PFDoS | 5.3 | U | 53 | 5.3 | 0.2 | U | 2 | 0.2 |  | ND | U | 28.1 | 8.8 | ND | U | 0.6 | 0.2 |  | ND | U | 0.1 | 0.04 | ND | U | 0.0 | 0.01 |
| PFEESA/PES | 5.3 | U | 16 | 5.3 | 0.2 | U | 0.6 | 0.2 |  | ND | U | 4.2 | 1.3 | ND | U | 0.0 | 0.0 |  | ND | U | 12.3 | 3.87 | ND | U | 1.8 | 0.58 |
| 9Cl-PF3ONS | 5.3 | U | 53 | 5.3 | 0.2 | U | 2 | 0.2 |  | ND | U | 8.4 | 2.7 | ND | U | 0.1 | 0.1 |  | ND | U | 0.8 | 0.27 | ND | U | 0.1 | 0.04 |
| 11Cl-PF3OUdS | 5.3 | U | 16 | 5.3 | 0.2 | U | 0.6 | 0.2 |  | ND | U | 7.0 | 2.2 | ND | U | 0.1 | 0.1 |  | ND | U | 0.8 | 0.27 | ND | U | 0.1 | 0.04 |

Site 9

| **Lab** | **Lab 1** | | | | | | | |  | **Lab 2** | | | | | | | |  | **Lab 3** | | | | | | | |
| --- | --- | --- | --- | --- | --- | --- | --- | --- | --- | --- | --- | --- | --- | --- | --- | --- | --- | --- | --- | --- | --- | --- | --- | --- | --- | --- |
| **Acronym** | **(Location A)** | | | | **(Location B)** | | | |  | **(Location A)** | | | | **(Location B)** | | | |  | **(Location A)** | | | | **(Location B)** | | | |
|  | R | Q | RL | MDL | R | Q | RL | MDL |  | R | Q | RL | MDL | R | Q | RL | MDL |  | R | Q | RL | MDL | R | Q | RL | MDL |
| 3:3 FTCA | 3.6 | U *- cn | 11 | 3.6 | 0.78 | U *- cn | 2.3 | 0.78 |  | ND | U | 27.6 | 8.7 | ND | U | 6.0 | 1.9 |  | ND | U | 10.4 | 3.29 | ND | U | 5.8 | 1.83 |
| 5:3 FTCA | 12 | *- cn | 11 | 3.6 | 41 | *- cn | 2.3 | 0.78 |  | ND | U | 27.6 | 8.7 | ND | U | 6.0 | 1.9 |  | 156.2 | N/A | 10.4 | 3.29 | 82.1 | N/A | 5.8 | 1.83 |
| 7:3 FTCA | 3.6 | U *- cn | 11 | 3.6 | 2.8 | *- cn | 2.3 | 0.78 |  | ND | U | 27.6 | 8.7 | ND | U | 6.0 | 1.9 |  | 17.7 | N/A | 2.9 | 0.92 | 27.4 | N/A | 1.6 | 0.51 |
| 4:2 FTS | 11 | U | 36 | 11 | 2.3 | U | 7.8 | 2.3 |  | ND | U | 4.6 | 1.5 | ND | U | 1.0 | 0.3 |  | ND | U | 0.3 | 0.09 | ND | U | 0.1 | 0.05 |
| 6:2 FTS | 11 | U | 36 | 11 | 2.3 | U | 7.8 | 2.3 |  | ND | U | 8.0 | 2.5 | ND | U | 1.7 | 0.6 |  | ND | U | 0.3 | 0.11 | ND | U | 0.1 | 0.06 |
| 8:2 FTS | 11 | U | 54 | 11 | 2.3 | U | 12 | 2.3 |  | ND | U | 6.3 | 2.0 | ND | U | 1.3 | 0.4 |  | 0.69 | N/A | 0.0 | 0.02 | 0.95 | N/A | 0.0 | 0.01 |
| PFMPA | 3.6 | U | 11 | 3.6 | 0.78 | U | 2.3 | 0.78 |  | ND | U | 5.7 | 1.8 | ND | U | 1.2 | 0.4 |  | ND | U | 1.5 | 0.49 | ND | U | 0.8 | 0.27 |
| PFMBA | 3.6 | U | 11 | 3.6 | 0.78 | U | 2.3 | 0.78 |  | ND | U | 2.8 | 0.9 | ND | U | 0.6 | 0.2 |  | ND | U | 3.0 | 0.95 | ND | U | 1.6 | 0.53 |
| NFDHA | 3.6 | U | 11 | 3.6 | 0.78 | U | 2.3 | 0.78 |  | ND | U | 5.1 | 1.6 | ND | U | 1.1 | 0.4 |  | ND | U | 0.5 | 0.18 | ND | U | 0.3 | 0.10 |
| HFPODA | 18 | U | 36 | 18 | 3.9 | U | 7.8 | 3.9 |  | ND | U | 6.9 | 2.2 | ND | U | 1.5 | 0.5 |  | ND | U | 11.2 | 3.55 | ND | U | 6.2 | 1.98 |
| DONA | 3.6 | U | 54 | 3.6 | 0.78 | U | 12 | 0.78 |  | ND | U | 5.7 | 1.8 | ND | U | 1.2 | 0.4 |  | ND | U | 0.8 | 0.25 | ND | U | 0.4 | 0.14 |
| NMeFOSAA | 3.6 | U | 36 | 3.6 | 5 | J | 7.8 | 0.78 |  | ND | U | 4.0 | 1.3 | ND | U | 0.8 | 0.3 |  | 3.4 | N/A | 0.2 | 0.09 | 8.3 | N/A | 0.1 | 0.05 |
| NEtFOSAA | 3.6 | U | 36 | 3.6 | 3.5 | J | 7.8 | 0.78 |  | ND | U | 2.8 | 0.9 | ND | U | 0.6 | 0.2 |  | 2.8 | N/A | 0.4 | 0.14 | 3.68 | N/A | 0.2 | 0.08 |
| PFOSA | 3.6 | U | 11 | 3.6 | 0.98 | J | 2.3 | 0.78 |  | ND | U | 2.3 | 0.7 | ND | U | 0.5 | 0.2 |  | 1.2 | N/A | 0.2 | 0.09 | 0.9 | N/A | 0.1 | 0.05 |
| NMeFOSA | 8.9 | U *+ cn | 36 | 8.9 | 1.9 | U *+ cn | 7.8 | 1.9 |  | ND | U | 2.3 | 0.7 | ND | U | 0.5 | 0.2 |  | ND | U | 2.4 | 0.77 | ND | U | 1.3 | 0.43 |
| NEtFOSA | 8.9 | U | 36 | 8.9 | 1.9 | U | 7.8 | 1.9 |  | ND | U | 2.3 | 0.7 | ND | U | 0.5 | 0.2 |  | ND | U | 2.6 | 0.84 | ND | U | 1.4 | 0.47 |
| NMeFOSE | 8.9 | U | 36 | 8.9 | 9.2 | N/A | 7.8 | 1.9 |  | ND | U | 23.0 | 7.3 | ND | U | 5.0 | 1.6 |  | 37.1 | N/A | 7.4 | 2.35 | 55.8 | N/A | 4.1 | 1.31 |
| NEtFOSE | 8.9 | U | 36 | 8.9 | 1.9 | U | 7.8 | 1.9 |  | ND | U | 23.0 | 7.3 | ND | U | 5.0 | 1.6 |  | ND | U | 5.7 | 2.85 | ND | U | 3.1 | 1.59 |
| PFBA | 14 | U | 36 | 14 | 3.1 | U | 7.8 | 3.1 |  | ND | U | 7.5 | 2.4 | ND | U | 1.6 | 0.5 |  | ND | U | 18.5 | 9.29 | ND | U | 10.3 | 5.17 |
| PFPeA | 3.6 | U | 11 | 3.6 | 0.78 | U | 2.3 | 0.78 |  | ND | U | 4.6 | 1.5 | ND | U | 1.0 | 0.3 |  | ND | U | 5.0 | 1.59 | ND | U | 2.8 | 0.88 |
| PFHxA | 3.6 | U | 11 | 3.6 | 1.5 | J | 2.3 | 0.78 |  | ND | U | 6.9 | 2.2 | ND | U | 1.5 | 0.5 |  | ND | U | 8.3 | 2.61 | ND | U | 4.6 | 1.45 |
| PFHpA | 3.6 | U | 11 | 3.6 | 0.78 | U | 2.3 | 0.78 |  | ND | U | 3.4 | 1.1 | ND | U | 0.7 | 0.2 |  | ND | U | 3.4 | 1.08 | ND | U | 1.9 | 0.60 |
| PFOA | 3.6 | U | 11 | 3.6 | 1 | J | 2.3 | 0.78 |  | ND | U | 4.0 | 1.3 | ND | U | 0.8 | 0.3 |  | ND | U | 3.6 | 1.15 | ND | U | 2.0 | 0.64 |
| PFNA | 3.6 | U | 11 | 3.6 | 1.3 | J | 2.3 | 0.78 |  | ND | U | 2.3 | 0.7 | ND | U | 0.5 | 0.2 |  | ND | U | 2.0 | 0.63 | ND | U | 1.1 | 0.35 |
| PFDA | 3.6 | U | 11 | 3.6 | 1.9 | J | 2.3 | 0.78 |  | ND | U | 2.8 | 0.9 | ND | U | 0.6 | 0.2 |  | ND | U | 2.0 | 0.66 | ND | U | 1.1 | 0.37 |
| PFUA | 3.6 | U | 11 | 3.6 | 1.7 | J | 2.3 | 0.78 |  | ND | U | 3.4 | 1.1 | ND | U | 0.7 | 0.2 |  | ND | U | 0.2 | 0.06 | 2.1 | N/A | 0.1 | 0.04 |
| PFDoA | 3.6 | U | 11 | 3.6 | 1.3 | J | 2.3 | 0.78 |  | ND | U | 4.6 | 1.5 | ND | U | 1.0 | 0.3 |  | ND | U | 0.4 | 0.15 | 1.1 | N/A | 0.2 | 0.08 |
| PFTrDA | 3.6 | U | 11 | 3.6 | 0.78 | U | 2.3 | 0.78 |  | ND | U | 4.6 | 1.5 | ND | U | 1.0 | 0.3 |  | ND | U | 0.6 | 0.20 | ND | U | 0.3 | 0.11 |
| PFTeA | 3.6 | U | 11 | 3.6 | 0.78 | U | 2.3 | 0.78 |  | ND | U | 9.8 | 3.1 | ND | U | 2.1 | 0.7 |  | ND | U | 2.5 | 0.81 | ND | U | 1.4 | 0.45 |
| PFBS | 7.1 | U | 36 | 7.1 | 1.6 | U | 7.8 | 1.6 |  | ND | U | 2.8 | 0.9 | ND | U | 0.6 | 0.2 |  | ND | U | 7.2 | 2.29 | ND | U | 4.0 | 1.27 |
| PFPS | 3.6 | U | 11 | 3.6 | 0.78 | U | 2.3 | 0.78 |  | ND | U | 4.0 | 1.3 | ND | U | 0.8 | 0.3 |  | ND | U | 1.5 | 0.48 | ND | U | 0.8 | 0.27 |
| PFHxS | 3.6 | U | 11 | 3.6 | 0.78 | U | 2.3 | 0.78 |  | ND | U | 6.3 | 2.0 | ND | U | 1.3 | 0.4 |  | ND | U | 3.1 | 0.98 | ND | U | 1.7 | 0.55 |
| PFHpS | 3.6 | U | 11 | 3.6 | 0.78 | U | 2.3 | 0.78 |  | ND | U | 4.6 | 1.5 | ND | U | 1.0 | 0.3 |  | ND | U | 1.6 | 0.51 | ND | U | 0.8 | 0.28 |
| PFOS | 3.6 | U | 11 | 3.6 | 9.8 | N/A | 2.3 | 0.78 |  | ND | U | 2.8 | 0.9 | 255.8 | N/A | 0.6 | 0.2 |  | ND | U | 10.6 | 3.36 | ND | U | 5.9 | 1.87 |
| PFNS | 3.6 | U | 11 | 3.6 | 0.78 | U | 2.3 | 0.78 |  | ND | U | 8.6 | 2.7 | ND | U | 1.8 | 0.6 |  | ND | U | 0.4 | 0.13 | ND | U | 0.2 | 0.07 |
| PFDS | 3.6 | U | 11 | 3.6 | 2 | J | 2.3 | 0.78 |  | ND | U | 5.1 | 1.6 | ND | U | 1.1 | 0.4 |  | ND | U | 0.2 | 0.08 | ND | U | 0.1 | 0.04 |
| PFDoS | 3.6 | U | 36 | 3.6 | 0.78 | U | 7.8 | 0.78 |  | ND | U | 11.5 | 3.6 | ND | U | 2.5 | 0.8 |  | ND | U | 0.1 | 0.04 | ND | U | 0.0 | 0.02 |
| PFEESA/PES | 3.6 | U | 11 | 3.6 | 0.78 | U | 2.3 | 0.78 |  | ND | U | 1.7 | 0.5 | ND | U | 0.3 | 0.1 |  | ND | U | 11.6 | 3.65 | ND | U | 6.4 | 2.03 |
| 9Cl-PF3ONS | 3.6 | U | 36 | 3.6 | 0.78 | U | 7.8 | 0.78 |  | ND | U | 3.4 | 1.1 | ND | U | 0.7 | 0.2 |  | ND | U | 0.8 | 0.25 | ND | U | 0.4 | 0.14 |
| 11Cl-PF3OUdS | 3.6 | U | 11 | 3.6 | 0.78 | U | 2.3 | 0.78 |  | ND | U | 2.8 | 0.9 | ND | U | 0.6 | 0.2 |  | ND | U | 0.8 | 0.25 | ND | U | 0.4 | 0.14 |

Site 10

| **Lab** | **Lab 1** | | | | | | | |  | **Lab 2** | | | | | | | |  | **Lab 3** | | | | | | | |
| --- | --- | --- | --- | --- | --- | --- | --- | --- | --- | --- | --- | --- | --- | --- | --- | --- | --- | --- | --- | --- | --- | --- | --- | --- | --- | --- |
| **Acronym** | **(Location A)** | | | | **(Location B)** | | | |  | **(Location A)** | | | | **(Location B)** | | | |  | **(Location A)** | | | | **(Location B)** | | | |
|  | R | Q | RL | MDL | R | Q | RL | MDL |  | R | Q | RL | MDL | R | Q | RL | MDL |  | R | Q | RL | MDL | R | Q | RL | MDL |
| 3:3 FTCA | 12 | U *- cn | 37 | 12 | 2.9 | J *- cn | 4.4 | 1.5 |  | ND | U | 26.0 | 8.2 | ND | U | 7.5 | 2.4 |  | ND | U | 10.7 | 3.38 | ND | U | 7.0 | 2.2 |
| 5:3 FTCA | 190 | *- cn | 37 | 12 | 270 | *- cn | 4.4 | 1.5 |  | ND | U | 26.0 | 8.2 | ND | U | 7.5 | 2.4 |  | 137.2 | N/A | 10.7 | 3.38 | 140.7 | N/A | 7.0 | 2.2 |
| 7:3 FTCA | 12 | U *- cn | 37 | 12 | 9.5 | *- cn | 4.4 | 1.5 |  | ND | U | 26.0 | 8.2 | ND | U | 7.5 | 2.4 |  | 29.3 | N/A | 3.0 | 0.94 | 31.6 | N/A | 1.9 | 0.6 |
| 4:2 FTS | 37 | U | 120 | 37 | 4.4 | U | 15 | 4.4 |  | ND | U | 4.3 | 1.4 | ND | U | 1.2 | 0.4 |  | ND | U | 0.3 | 0.10 | ND | U | 0.2 | 0.1 |
| 6:2 FTS | 37 | U | 120 | 37 | 4.4 | U | 15 | 4.4 |  | ND | U | 7.5 | 2.4 | ND | U | 2.1 | 0.7 |  | ND | U | 0.3 | 0.11 | ND | U | 0.2 | 0.1 |
| 8:2 FTS | 37 | U | 180 | 37 | 4.4 | U | 22 | 4.4 |  | ND | U | 5.9 | 1.9 | ND | U | 1.7 | 0.5 |  | ND | U | 0.0 | 0.02 | ND | U | 0.0 | 0.0 |
| PFMPA | 12 | U | 37 | 12 | 1.5 | U | 4.4 | 1.5 |  | ND | U | 5.4 | 1.7 | ND | U | 1.5 | 0.5 |  | ND | U | 1.5 | 0.50 | ND | U | 1.0 | 0.3 |
| PFMBA | 12 | U | 37 | 12 | 1.5 | U | 4.4 | 1.5 |  | ND | U | 2.7 | 0.9 | ND | U | 0.7 | 0.2 |  | ND | U | 3.0 | 0.97 | ND | U | 2.0 | 0.6 |
| NFDHA | 12 | U | 37 | 12 | 1.5 | U | 4.4 | 1.5 |  | ND | U | 4.8 | 1.5 | ND | U | 1.4 | 0.4 |  | ND | U | 0.5 | 0.18 | ND | U | 0.3 | 0.1 |
| HFPODA | 61 | U | 120 | 61 | 7.3 | U | 15 | 7.3 |  | ND | U | 6.5 | 2.0 | ND | U | 1.8 | 0.6 |  | ND | U | 11.5 | 3.64 | ND | U | 7.6 | 2.4 |
| DONA | 12 | U | 180 | 12 | 1.5 | U | 22 | 1.5 |  | ND | U | 5.4 | 1.7 | ND | U | 1.5 | 0.5 |  | ND | U | 0.8 | 0.26 | ND | U | 0.5 | 0.2 |
| NMeFOSAA | 12 | U | 120 | 12 | 12 | J | 15 | 1.5 |  | ND | U | 3.7 | 1.2 | ND | U | 1.0 | 0.3 |  | 3.4 | N/A | 0.2 | 0.09 | 4.5 | N/A | 0.1 | 0.1 |
| NEtFOSAA | 12 | U | 120 | 12 | 7.4 | J | 15 | 1.5 |  | ND | U | 2.7 | 0.9 | ND | U | 0.7 | 0.2 |  | 1.2 | N/A | 0.4 | 0.14 | 2.74 | N/A | 0.2 | 0.1 |
| PFOSA | 12 | U | 37 | 12 | 1.7 | J | 4.4 | 1.5 |  | ND | U | 2.1 | 0.7 | ND | U | 0.6 | 0.2 |  | ND | U | 0.2 | 0.09 | ND | U | 0.1 | 0.1 |
| NMeFOSA | 31 | U *+ cn | 120 | 31 | 3.6 | U *+ cn | 15 | 3.6 |  | ND | U | 2.1 | 0.7 | ND | U | 0.6 | 0.2 |  | ND | U | 2.4 | 0.79 | ND | U | 1.6 | 0.5 |
| NEtFOSA | 31 | U | 120 | 31 | 3.6 | U | 15 | 3.6 |  | ND | U | 2.1 | 0.7 | ND | U | 0.6 | 0.2 |  | ND | U | 2.7 | 0.86 | ND | U | 1.8 | 0.6 |
| NMeFOSE | 31 | U | 120 | 31 | 6.1 | J | 15 | 3.6 |  | ND | U | 21.6 | 6.8 | ND | U | 6.2 | 2.0 |  | 12.5 | N/A | 7.6 | 2.41 | 13.8 | N/A | 5.0 | 1.6 |
| NEtFOSE | 31 | U | 120 | 31 | 3.6 | U | 15 | 3.6 |  | ND | U | 21.6 | 6.8 | ND | U | 6.2 | 2.0 |  | ND | U | 5.8 | 2.93 | ND | U | 3.8 | 1.9 |
| PFBA | 49 | U | 120 | 49 | 5.8 | U | 15 | 5.8 |  | ND | U | 7.0 | 2.2 | ND | U | 2.0 | 0.6 |  | ND | U | 19.0 | 9.53 | ND | U | 12.5 | 6.3 |
| PFPeA | 12 | U | 37 | 12 | 2.6 | J | 4.4 | 1.5 |  | ND | U | 4.3 | 1.4 | ND | U | 1.2 | 0.4 |  | ND | U | 5.1 | 1.63 | ND | U | 3.4 | 1.1 |
| PFHxA | 12 | U | 37 | 12 | 8.9 | N/A | 4.4 | 1.5 |  | ND | U | 6.5 | 2.0 | ND | U | 1.8 | 0.6 |  | ND | U | 8.5 | 2.68 | ND | U | 5.6 | 1.8 |
| PFHpA | 12 | U | 37 | 12 | 1.5 | U | 4.4 | 1.5 |  | ND | U | 3.2 | 1.0 | ND | U | 0.9 | 0.3 |  | ND | U | 3.5 | 1.11 | ND | U | 2.3 | 0.7 |
| PFOA | 12 | U | 37 | 12 | 2.9 | J | 4.4 | 1.5 |  | ND | U | 3.7 | 1.2 | ND | U | 1.0 | 0.3 |  | ND | U | 3.7 | 1.18 | ND | U | 2.4 | 0.8 |
| PFNA | 12 | U | 37 | 12 | 1.5 | U | 4.4 | 1.5 |  | ND | U | 2.1 | 0.7 | ND | U | 0.6 | 0.2 |  | ND | U | 2.0 | 0.65 | ND | U | 1.3 | 0.4 |
| PFDA | 12 | U | 37 | 12 | 4.1 | J | 4.4 | 1.5 |  | ND | U | 2.7 | 0.9 | ND | U | 0.7 | 0.2 |  | 4.22 | N/A | 2.1 | 0.68 | 5.2 | N/A | 1.4 | 0.4 |
| PFUA | 12 | U | 37 | 12 | 2.7 | J | 4.4 | 1.5 |  | ND | U | 3.2 | 1.0 | ND | U | 0.9 | 0.3 |  | ND | U | 0.2 | 0.06 | 1.0 | N/A | 0.1 | 0.0 |
| PFDoA | 12 | U | 37 | 12 | 4.4 | N/A | 4.4 | 1.5 |  | ND | U | 4.3 | 1.4 | ND | U | 1.2 | 0.4 |  | 2.6 | N/A | 0.4 | 0.16 | 2.1 | N/A | 0.3 | 0.1 |
| PFTrDA | 12 | U | 37 | 12 | 1.5 | U | 4.4 | 1.5 |  | ND | U | 4.3 | 1.4 | ND | U | 1.2 | 0.4 |  | ND | U | 0.6 | 0.21 | ND | U | 0.4 | 0.1 |
| PFTeA | 12 | U | 37 | 12 | 1.8 | J | 4.4 | 1.5 |  | ND | U | 9.2 | 2.9 | ND | U | 2.6 | 0.8 |  | ND | U | 2.6 | 0.83 | ND | U | 1.7 | 0.5 |
| PFBS | 25 | U | 120 | 25 | 2.9 | U | 15 | 2.9 |  | ND | U | 2.7 | 0.9 | ND | U | 0.7 | 0.2 |  | ND | U | 7.4 | 2.35 | ND | U | 4.9 | 1.5 |
| PFPS | 12 | U | 37 | 12 | 1.5 | U | 4.4 | 1.5 |  | ND | U | 3.7 | 1.2 | ND | U | 1.0 | 0.3 |  | ND | U | 1.5 | 0.49 | ND | U | 1.0 | 0.3 |
| PFHxS | 12 | U | 37 | 12 | 1.5 | U | 4.4 | 1.5 |  | ND | U | 5.9 | 1.9 | ND | U | 1.7 | 0.5 |  | ND | U | 3.2 | 1.01 | ND | U | 2.1 | 0.7 |
| PFHpS | 12 | U | 37 | 12 | 1.5 | U | 4.4 | 1.5 |  | ND | U | 4.3 | 1.4 | ND | U | 1.2 | 0.4 |  | ND | U | 1.6 | 0.52 | ND | U | 1.0 | 0.3 |
| PFOS | 12 | U | 37 | 12 | 16 | N/A | 4.4 | 1.5 |  | 215.7 | N/A | 2.7 | 0.9 | 66.9 | N/A | 0.7 | 0.2 |  | ND | U | 10.9 | 3.45 | ND | U | 7.2 | 2.3 |
| PFNS | 12 | U | 37 | 12 | 1.5 | U | 4.4 | 1.5 |  | ND | U | 8.1 | 2.6 | ND | U | 2.3 | 0.7 |  | ND | U | 0.4 | 0.14 | ND | U | 0.2 | 0.1 |
| PFDS | 12 | U | 37 | 12 | 1.5 | U | 4.4 | 1.5 |  | ND | U | 4.8 | 1.5 | ND | U | 1.4 | 0.4 |  | ND | U | 0.2 | 0.08 | ND | U | 0.1 | 0.1 |
| PFDoS | 12 | U | 120 | 12 | 1.5 | U | 15 | 1.5 |  | ND | U | 10.8 | 3.4 | ND | U | 3.1 | 1.0 |  | ND | U | 0.1 | 0.04 | ND | U | 0.0 | 0.0 |
| PFEESA/PES | 12 | U | 37 | 12 | 1.5 | U | 4.4 | 1.5 |  | ND | U | 1.6 | 0.5 | ND | U | 0.4 | 0.1 |  | ND | U | 11.9 | 3.75 | ND | U | 7.8 | 2.5 |
| 9Cl-PF3ONS | 12 | U | 120 | 12 | 1.5 | U | 15 | 1.5 |  | ND | U | 3.2 | 1.0 | ND | U | 0.9 | 0.3 |  | ND | U | 0.8 | 0.26 | ND | U | 0.5 | 0.2 |
| 11Cl-PF3OUdS | 12 | U | 37 | 12 | 1.5 | U | 4.4 | 1.5 |  | ND | U | 2.7 | 0.9 | ND | U | 0.7 | 0.2 |  | ND | U | 0.8 | 0.26 | ND | U | 0.5 | 0.2 |

Site 11

| **Lab** | **Lab 1** | | | | | | | |  | **Lab 2** | | | | | | | |  | **Lab 3** | | | | | | | |
| --- | --- | --- | --- | --- | --- | --- | --- | --- | --- | --- | --- | --- | --- | --- | --- | --- | --- | --- | --- | --- | --- | --- | --- | --- | --- | --- |
| **Acronym** | **(Location A)** | | | | **(Location B)** | | | |  | **(Location A)** | | | | **(Location B)** | | | |  | **(Location A)** | | | | **(Location B)** | | | |
|  | R | Q | RL | MDL | R | Q | RL | MDL |  | R | Q | RL | MDL | R | Q | RL | MDL |  | R | Q | RL | MDL | R | Q | RL | MDL |
| 3:3 FTCA | 2.6 | U *- cn | 7.7 | 2.6 | 1.2 | U *- cn | 3.5 | 1.2 |  | ND | U | 206.1 | 64.8 | ND | U | 9.2 | 2.9 |  | ND | U | 7.0 | 2.23 | ND | U | 9.2 | 2.90 |
| 5:3 FTCA | 15 | *- cn | 7.7 | 2.6 | 56 | *- cn | 3.5 | 1.2 |  | ND | U | 206.1 | 64.8 | 163.5 | N/A | 9.2 | 2.9 |  | 635.4 | N/A | 7.0 | 2.23 | 657.8 | N/A | 9.2 | 2.90 |
| 7:3 FTCA | 2.6 | U *- cn | 7.7 | 2.6 | 2.2 | J *- cn | 3.5 | 1.2 |  | ND | U | 206.1 | 64.8 | ND | U | 9.2 | 2.9 |  | 44.6 | N/A | 1.9 | 0.62 | 105.2 | N/A | 2.5 | 0.81 |
| 4:2 FTS | 7.7 | U | 26 | 7.7 | 3.5 | U | 12 | 3.5 |  | ND | U | 34.3 | 10.8 | ND | U | 1.5 | 0.5 |  | ND | U | 0.2 | 0.06 | ND | U | 0.2 | 0.08 |
| 6:2 FTS | 7.7 | U | 26 | 7.7 | 3.5 | U | 12 | 3.5 |  | ND | U | 60.1 | 18.9 | ND | U | 2.7 | 0.9 |  | 5.63 | N/A | 0.2 | 0.07 | 1.39 | N/A | 0.3 | 0.09 |
| 8:2 FTS | 7.7 | U | 39 | 7.7 | 3.5 | U | 17 | 3.5 |  | ND | U | 47.2 | 14.9 | ND | U | 2.1 | 0.7 |  | ND | U | 0.0 | 0.01 | ND | U | 0.0 | 0.02 |
| PFMPA | 2.6 | U | 7.7 | 2.6 | 1.2 | U | 3.5 | 1.2 |  | ND | U | 42.9 | 13.5 | ND | U | 1.9 | 0.6 |  | ND | U | 1.0 | 0.33 | ND | U | 1.3 | 0.43 |
| PFMBA | 2.6 | U | 7.7 | 2.6 | 1.2 | U | 3.5 | 1.2 |  | ND | U | 21.4 | 6.8 | ND | U | 0.9 | 0.3 |  | ND | U | 2.0 | 0.64 | ND | U | 2.6 | 0.84 |
| NFDHA | 2.6 | U | 7.7 | 2.6 | 1.2 | U | 3.5 | 1.2 |  | ND | U | 38.6 | 12.2 | ND | U | 1.7 | 0.5 |  | ND | U | 0.3 | 0.12 | ND | U | 0.4 | 0.16 |
| HFPODA | 13 | U | 26 | 13 | 5.8 | U | 12 | 5.8 |  | ND | U | 51.5 | 16.2 | ND | U | 2.3 | 0.7 |  | ND | U | 7.6 | 2.41 | ND | U | 9.9 | 3.13 |
| DONA | 2.6 | U | 39 | 2.6 | 1.2 | U | 17 | 1.2 |  | ND | U | 42.9 | 13.5 | ND | U | 1.9 | 0.6 |  | ND | U | 0.5 | 0.17 | ND | U | 0.7 | 0.22 |
| NMeFOSAA | 2.6 | U | 26 | 2.6 | 4.7 | J | 12 | 1.2 |  | ND | U | 30.0 | 9.5 | ND | U | 1.3 | 0.4 |  | 3.5 | N/A | 0.1 | 0.06 | 13.1 | N/A | 0.2 | 0.08 |
| NEtFOSAA | 2.6 | U | 26 | 2.6 | 2.8 | J | 12 | 1.2 |  | ND | U | 21.4 | 6.8 | ND | U | 0.9 | 0.3 |  | 2.8 | N/A | 0.3 | 0.09 | 8.79 | N/A | 0.3 | 0.12 |
| PFOSA | 2.6 | U | 7.7 | 2.6 | 1.2 | U | 3.5 | 1.2 |  | ND | U | 17.1 | 5.4 | ND | U | 0.7 | 0.2 |  | ND | U | 0.1 | 0.06 | ND | U | 0.2 | 0.08 |
| NMeFOSA | 6.5 | U *+ cn | 26 | 6.5 | 2.9 | U *+ | 12 | 2.9 |  | ND | U | 17.1 | 5.4 | ND | U | 0.7 | 0.2 |  | ND | U | 1.6 | 0.52 | ND | U | 2.1 | 0.68 |
| NEtFOSA | 6.5 | U | 26 | 6.5 | 2.9 | U | 12 | 2.9 |  | ND | U | 17.1 | 5.4 | ND | U | 0.7 | 0.2 |  | ND | U | 1.8 | 0.57 | ND | U | 2.3 | 0.74 |
| NMeFOSE | 6.5 | U | 26 | 6.5 | 3.8 | J | 12 | 2.9 |  | ND | U | 171.7 | 54.0 | ND | U | 7.7 | 2.4 |  | 11.5 | N/A | 5.0 | 1.59 | 14.7 | N/A | 6.5 | 2.07 |
| NEtFOSE | 6.5 | U | 26 | 6.5 | 2.9 | U | 12 | 2.9 |  | ND | U | 171.7 | 54.0 | ND | U | 7.7 | 2.4 |  | ND | U | 3.8 | 1.93 | ND | U | 5.0 | 2.52 |
| PFBA | 10 | U | 26 | 10 | 4.6 | U | 12 | 4.6 |  | ND | U | 55.8 | 17.6 | ND | U | 2.5 | 0.8 |  | ND | U | 12.6 | 6.30 | ND | U | 16.4 | 8.20 |
| PFPeA | 2.6 | U | 7.7 | 2.6 | 1.2 | U | 3.5 | 1.2 |  | ND | U | 34.3 | 10.8 | ND | U | 1.5 | 0.5 |  | ND | U | 3.4 | 1.08 | ND | U | 4.4 | 1.40 |
| PFHxA | 2.6 | U | 7.7 | 2.6 | 1.2 | U | 3.5 | 1.2 |  | ND | U | 51.5 | 16.2 | ND | U | 2.3 | 0.7 |  | 13.0 | N/A | 5.6 | 1.77 | 18.1 | N/A | 7.3 | 2.31 |
| PFHpA | 2.6 | U | 7.7 | 2.6 | 1.2 | U | 3.5 | 1.2 |  | ND | U | 25.7 | 8.1 | ND | U | 1.1 | 0.4 |  | ND | U | 2.3 | 0.73 | ND | U | 3.0 | 0.96 |
| PFOA | 2.6 | U | 7.7 | 2.6 | 1.2 | U | 3.5 | 1.2 |  | ND | U | 30.0 | 9.5 | ND | U | 1.3 | 0.4 |  | ND | U | 2.4 | 0.78 | ND | U | 3.2 | 1.01 |
| PFNA | 2.6 | U | 7.7 | 2.6 | 1.2 | U | 3.5 | 1.2 |  | ND | U | 17.1 | 5.4 | ND | U | 0.7 | 0.2 |  | ND | U | 1.3 | 0.43 | ND | U | 1.7 | 0.56 |
| PFDA | 3.5 | J | 7.7 | 2.6 | 5 | N/A | 3.5 | 1.2 |  | ND | U | 21.4 | 6.8 | ND | U | 0.9 | 0.3 |  | 2.15 | N/A | 1.4 | 0.45 | 4.3 | N/A | 1.8 | 0.58 |
| PFUA | 2.6 | U | 7.7 | 2.6 | 1.2 | U | 3.5 | 1.2 |  | ND | U | 25.7 | 8.1 | ND | U | 1.1 | 0.4 |  | 1.7 | N/A | 0.1 | 0.04 | 3.8 | N/A | 0.1 | 0.06 |
| PFDoA | 2.6 | U | 7.7 | 2.6 | 2 | J | 3.5 | 1.2 |  | ND | U | 34.3 | 10.8 | ND | U | 1.5 | 0.5 |  | 3.2 | N/A | 0.3 | 0.10 | 3.8 | N/A | 0.4 | 0.13 |
| PFTrDA | 2.6 | U | 7.7 | 2.6 | 1.2 | U | 3.5 | 1.2 |  | ND | U | 34.3 | 10.8 | ND | U | 1.5 | 0.5 |  | ND | U | 0.4 | 0.14 | ND | U | 0.5 | 0.18 |
| PFTeA | 2.6 | U | 7.7 | 2.6 | 1.2 | U | 3.5 | 1.2 |  | ND | U | 73.0 | 23.0 | ND | U | 3.2 | 1.0 |  | ND | U | 1.7 | 0.55 | ND | U | 2.2 | 0.71 |
| PFBS | 5.2 | U | 26 | 5.2 | 2.3 | U | 12 | 2.3 |  | ND | U | 21.4 | 6.8 | ND | U | 0.9 | 0.3 |  | ND | U | 4.9 | 1.55 | ND | U | 6.4 | 2.02 |
| PFPS | 2.6 | U | 7.7 | 2.6 | 1.2 | U | 3.5 | 1.2 |  | ND | U | 30.0 | 9.5 | ND | U | 1.3 | 0.4 |  | ND | U | 1.0 | 0.33 | ND | U | 1.3 | 0.42 |
| PFHxS | 2.6 | U | 7.7 | 2.6 | 1.2 | U | 3.5 | 1.2 |  | ND | U | 47.2 | 14.9 | ND | U | 2.1 | 0.7 |  | ND | U | 2.1 | 0.67 | ND | U | 2.7 | 0.87 |
| PFHpS | 2.6 | U | 7.7 | 2.6 | 1.2 | U | 3.5 | 1.2 |  | ND | U | 34.3 | 10.8 | ND | U | 1.5 | 0.5 |  | ND | U | 1.0 | 0.34 | ND | U | 1.4 | 0.45 |
| PFOS | 2.6 | U | 7.7 | 2.6 | 8.8 | N/A | 3.5 | 1.2 |  | 349.9 | N/A | 21.4 | 6.8 | ND | U | 0.9 | 0.3 |  | ND | U | 7.2 | 2.28 | ND | U | 9.4 | 2.97 |
| PFNS | 2.6 | U | 7.7 | 2.6 | 1.2 | U | 3.5 | 1.2 |  | ND | U | 64.4 | 20.3 | ND | U | 2.9 | 0.9 |  | ND | U | 0.2 | 0.09 | ND | U | 0.3 | 0.12 |
| PFDS | 2.6 | U | 7.7 | 2.6 | 1.2 | U | 3.5 | 1.2 |  | ND | U | 38.6 | 12.2 | ND | U | 1.7 | 0.5 |  | ND | U | 0.1 | 0.05 | ND | U | 0.2 | 0.07 |
| PFDoS | 2.6 | U | 26 | 2.6 | 1.2 | U | 12 | 1.2 |  | ND | U | 85.8 | 27.0 | ND | U | 3.8 | 1.2 |  | ND | U | 0.0 | 0.03 | ND | U | 0.1 | 0.03 |
| PFEESA/PES | 2.6 | U | 7.7 | 2.6 | 1.2 | U | 3.5 | 1.2 |  | ND | U | 12.8 | 4.1 | ND | U | 0.5 | 0.2 |  | ND | U | 7.8 | 2.48 | ND | U | 10.2 | 3.22 |
| 9Cl-PF3ONS | 2.6 | U | 26 | 2.6 | 1.2 | U | 12 | 1.2 |  | ND | U | 25.7 | 8.1 | ND | U | 1.1 | 0.4 |  | ND | U | 0.5 | 0.17 | ND | U | 0.7 | 0.22 |
| 11Cl-PF3OUdS | 2.6 | U | 7.7 | 2.6 | 1.2 | U | 3.5 | 1.2 |  | ND | U | 21.4 | 6.8 | ND | U | 0.9 | 0.3 |  | ND | U | 0.5 | 0.17 | ND | U | 0.7 | 0.22 |

Site 12

| **Lab** | **Lab 1** | | | | | | | |  | **Lab 2** | | | | | | | |  | **Lab 3** | | | | | | | |
| --- | --- | --- | --- | --- | --- | --- | --- | --- | --- | --- | --- | --- | --- | --- | --- | --- | --- | --- | --- | --- | --- | --- | --- | --- | --- | --- |
| **Acronym** | **(Location A)** | | | | **(Location B)** | | | |  | **(Location A)** | | | | **(Location B)** | | | |  | **(Location A)** | | | | **(Location B)** | | | |
|  | R | Q | RL | MDL | R | Q | RL | MDL |  | R | Q | RL | MDL | R | Q | RL | MDL |  | R | Q | RL | MDL | R | Q | RL | MDL |
| 3:3 FTCA | 3.3 | U *- cn | 9.9 | 3.3 | 1.1 | U *- cn | 3.4 | 1.1 |  | ND | U | 31.6 | 10.0 | ND | U | 7.6 | 2.4 |  | ND | U | 9.7 | 3.07 | ND | U | 8.6 | 2.71 |
| 5:3 FTCA | 33 | *- cn | 9.9 | 3.3 | 100 | *- cn | 3.4 | 1.1 |  | ND | U | 31.6 | 10.0 | 20.4 | N/A | 7.6 | 2.4 |  | 134.4 | N/A | 9.7 | 3.07 | 218.8 | N/A | 8.6 | 2.71 |
| 7:3 FTCA | 3.3 | U *- cn | 9.9 | 3.3 | 3 | J *- cn | 3.4 | 1.1 |  | ND | U | 31.6 | 10.0 | ND | U | 7.6 | 2.4 |  | 26.7 | N/A | 2.7 | 0.86 | 62.8 | N/A | 2.4 | 0.76 |
| 4:2 FTS | 9.9 | U | 33 | 9.9 | 3.4 | U | 11 | 3.4 |  | ND | U | 5.2 | 1.7 | ND | U | 1.2 | 0.4 |  | ND | U | 0.2 | 0.09 | ND | U | 0.2 | 0.08 |
| 6:2 FTS | 9.9 | U | 33 | 9.9 | 3.4 | U | 11 | 3.4 |  | ND | U | 9.2 | 2.9 | ND | U | 2.2 | 0.7 |  | ND | U | 0.3 | 0.10 | ND | U | 0.2 | 0.09 |
| 8:2 FTS | 9.9 | U | 49 | 9.9 | 3.4 | U | 17 | 3.4 |  | ND | U | 7.2 | 2.3 | ND | U | 1.7 | 0.5 |  | 0.74 | N/A | 0.0 | 0.02 | 1.17 | N/A | 0.0 | 0.02 |
| PFMPA | 3.3 | U | 9.9 | 3.3 | 1.1 | U | 3.4 | 1.1 |  | ND | U | 6.6 | 2.1 | ND | U | 1.5 | 0.5 |  | ND | U | 1.4 | 0.45 | ND | U | 1.2 | 0.40 |
| PFMBA | 3.3 | U *- cn | 9.9 | 3.3 | 1.1 | U *- cn | 3.4 | 1.1 |  | ND | U | 3.3 | 1.0 | ND | U | 0.7 | 0.2 |  | ND | U | 2.8 | 0.88 | ND | U | 2.4 | 0.78 |
| NFDHA | 3.3 | U *- cn | 9.9 | 3.3 | 1.1 | U *- cn | 3.4 | 1.1 |  | ND | U | 5.9 | 1.9 | ND | U | 1.4 | 0.4 |  | ND | U | 0.5 | 0.17 | ND | U | 0.4 | 0.15 |
| HFPODA | 16 | U | 33 | 16 | 5.6 | U | 11 | 5.6 |  | ND | U | 7.9 | 2.5 | ND | U | 1.9 | 0.6 |  | ND | U | 10.5 | 3.31 | ND | U | 9.2 | 2.92 |
| DONA | 3.3 | U | 49 | 3.3 | 1.1 | U | 17 | 1.1 |  | ND | U | 6.6 | 2.1 | ND | U | 1.5 | 0.5 |  | ND | U | 0.7 | 0.24 | ND | U | 0.6 | 0.21 |
| NMeFOSAA | 6.3 | J I | 33 | 3.3 | 17 | N/A | 11 | 1.1 |  | ND | U | 4.6 | 1.5 | ND | U | 1.1 | 0.3 |  | 6.3 | N/A | 0.2 | 0.08 | 16.89 | N/A | 0.2 | 0.07 |
| NEtFOSAA | 3.3 | U | 33 | 3.3 | 6.7 | J | 11 | 1.1 |  | ND | U | 3.3 | 1.0 | ND | U | 0.7 | 0.2 |  | 4.6 | N/A | 0.4 | 0.13 | 7.6 | N/A | 0.3 | 0.11 |
| PFOSA | 3.3 | U | 9.9 | 3.3 | 1.1 | U | 3.4 | 1.1 |  | ND | U | 2.6 | 0.8 | ND | U | 0.6 | 0.2 |  | ND | U | 0.2 | 0.08 | ND | U | 0.2 | 0.07 |
| NMeFOSA | 8.2 | U | 33 | 8.2 | 2.8 | U | 11 | 2.8 |  | ND | U | 2.6 | 0.8 | ND | U | 0.6 | 0.2 |  | ND | U | 2.2 | 0.71 | ND | U | 2.0 | 0.63 |
| NEtFOSA | 8.2 | U | 33 | 8.2 | 2.8 | U | 11 | 2.8 |  | ND | U | 2.6 | 0.8 | ND | U | 0.6 | 0.2 |  | ND | U | 2.4 | 0.78 | ND | U | 2.2 | 0.69 |
| NMeFOSE | 8.2 | U | 33 | 8.2 | 6.2 | J | 11 | 2.8 |  | ND | U | 26.4 | 8.3 | ND | U | 6.3 | 2.0 |  | 18.4 | N/A | 6.9 | 2.19 | 36.5 | N/A | 6.1 | 1.93 |
| NEtFOSE | 8.2 | U | 33 | 8.2 | 4.6 | J | 11 | 2.8 |  | ND | U | 26.4 | 8.3 | ND | U | 6.3 | 2.0 |  | ND | U | 5.3 | 2.66 | ND | U | 4.6 | 2.35 |
| PFBA | 13 | U | 33 | 13 | 4.5 | U | 11 | 4.5 |  | ND | U | 8.5 | 2.7 | ND | U | 2.0 | 0.6 |  | ND | U | 17.3 | 8.66 | ND | U | 15.2 | 7.65 |
| PFPeA | 3.3 | U | 9.9 | 3.3 | 1.1 | U | 3.4 | 1.1 |  | ND | U | 5.2 | 1.7 | ND | U | 1.2 | 0.4 |  | ND | U | 4.7 | 1.48 | ND | U | 4.1 | 1.31 |
| PFHxA | 3.3 | U | 9.9 | 3.3 | 1.7 | J | 3.4 | 1.1 |  | ND | U | 7.9 | 2.5 | ND | U | 1.9 | 0.6 |  | ND | U | 7.7 | 2.44 | ND | U | 6.8 | 2.15 |
| PFHpA | 3.3 | U | 9.9 | 3.3 | 1.1 | U | 3.4 | 1.1 |  | ND | U | 3.9 | 1.2 | ND | U | 0.9 | 0.3 |  | ND | U | 3.2 | 1.01 | ND | U | 2.8 | 0.89 |
| PFOA | 3.3 | U | 9.9 | 3.3 | 1.1 | U | 3.4 | 1.1 |  | ND | U | 4.6 | 1.5 | ND | U | 1.1 | 0.3 |  | ND | U | 3.3 | 1.07 | ND | U | 2.9 | 0.94 |
| PFNA | 3.3 | U | 9.9 | 3.3 | 1.1 | U | 3.4 | 1.1 |  | ND | U | 2.6 | 0.8 | ND | U | 0.6 | 0.2 |  | ND | U | 1.8 | 0.59 | ND | U | 1.6 | 0.52 |
| PFDA | 3.3 | U | 9.9 | 3.3 | 4.8 | N/A | 3.4 | 1.1 |  | ND | U | 3.3 | 1.0 | ND | U | 0.7 | 0.2 |  | ND | U | 1.9 | 0.61 | ND | U | 1.7 | 0.54 |
| PFUA | 3.3 | U | 9.9 | 3.3 | 1.3 | J | 3.4 | 1.1 |  | ND | U | 3.9 | 1.2 | ND | U | 0.9 | 0.3 |  | ND | U | 0.1 | 0.06 | ND | U | 0.1 | 0.05 |
| PFDoA | 3.3 | U | 9.9 | 3.3 | 2.2 | J | 3.4 | 1.1 |  | ND | U | 5.2 | 1.7 | ND | U | 1.2 | 0.4 |  | 0.9 | N/A | 0.4 | 0.14 | 8.3 | N/A | 0.3 | 0.13 |
| PFTrDA | 3.3 | U | 9.9 | 3.3 | 1.1 | U | 3.4 | 1.1 |  | ND | U | 5.2 | 1.7 | ND | U | 1.2 | 0.4 |  | ND | U | 0.6 | 0.19 | ND | U | 0.5 | 0.17 |
| PFTeA | 3.3 | U | 9.9 | 3.3 | 1.1 | U | 3.4 | 1.1 |  | ND | U | 11.2 | 3.5 | ND | U | 2.7 | 0.8 |  | ND | U | 2.3 | 0.75 | ND | U | 2.1 | 0.67 |
| PFBS | 6.6 | U | 33 | 6.6 | 2.3 | U | 11 | 2.3 |  | ND | U | 3.3 | 1.0 | ND | U | 0.7 | 0.2 |  | ND | U | 6.7 | 2.14 | ND | U | 5.9 | 1.89 |
| PFPS | 3.3 | U | 9.9 | 3.3 | 1.1 | U | 3.4 | 1.1 |  | ND | U | 4.6 | 1.5 | ND | U | 1.1 | 0.3 |  | ND | U | 1.4 | 0.45 | ND | U | 1.2 | 0.40 |
| PFHxS | 3.3 | U | 9.9 | 3.3 | 1.1 | J I | 3.4 | 1.1 |  | ND | U | 7.2 | 2.3 | ND | U | 1.7 | 0.5 |  | ND | U | 2.9 | 0.91 | ND | U | 2.5 | 0.81 |
| PFHpS | 3.3 | U | 9.9 | 3.3 | 1.1 | U | 3.4 | 1.1 |  | ND | U | 5.2 | 1.7 | ND | U | 1.2 | 0.4 |  | ND | U | 1.5 | 0.47 | ND | U | 1.3 | 0.42 |
| PFOS | 3.3 | U | 9.9 | 3.3 | 6.5 | N/A | 3.4 | 1.1 |  | 51.9 | N/A | 3.3 | 1.0 | ND | U | 0.7 | 0.2 |  | ND | U | 9.9 | 3.13 | ND | U | 8.7 | 2.77 |
| PFNS | 3.3 | U | 9.9 | 3.3 | 1.1 | U | 3.4 | 1.1 |  | ND | U | 9.9 | 3.1 | ND | U | 2.3 | 0.7 |  | ND | U | 0.3 | 0.12 | ND | U | 0.3 | 0.11 |
| PFDS | 3.3 | U | 9.9 | 3.3 | 1.1 | U | 3.4 | 1.1 |  | ND | U | 5.9 | 1.9 | ND | U | 1.4 | 0.4 |  | ND | U | 0.2 | 0.07 | ND | U | 0.1 | 0.06 |
| PFDoS | 7.8 | J | 33 | 3.3 | 1.8 | J | 11 | 1.1 |  | ND | U | 13.2 | 4.2 | ND | U | 3.1 | 1.0 |  | ND | U | 0.1 | 0.04 | ND | U | 0.0 | 0.03 |
| PFEESA/PES | 3.3 | U | 9.9 | 3.3 | 1.1 | U | 3.4 | 1.1 |  | ND | U | 1.9 | 0.6 | ND | U | 0.4 | 0.1 |  | ND | U | 10.8 | 3.40 | ND | U | 9.5 | 3.01 |
| 9Cl-PF3ONS | 3.3 | U | 33 | 3.3 | 1.1 | U | 11 | 1.1 |  | ND | U | 3.9 | 1.2 | ND | U | 0.9 | 0.3 |  | ND | U | 0.7 | 0.24 | ND | U | 0.6 | 0.21 |
| 11Cl-PF3OUdS | 3.3 | U | 9.9 | 3.3 | 1.1 | U | 3.4 | 1.1 |  | ND | U | 3.3 | 1.0 | ND | U | 0.7 | 0.2 |  | ND | U | 0.7 | 0.24 | ND | U | 0.6 | 0.21 |

Notes: R = Results (ng/g, dry basis); Q = Qualifiers; U = Undetected/Not detected; *5+ = Isotope dilution analyte is outside acceptance limits, high bias; *5- = Isotope dilution analyte is outside acceptance limits, low bias; J, D = Result is less than the RL but greater than or equal to the MDL and the concentration is an approximate value; F1 = MS and/or MSD recovery exceeds control limits. (Matrix spike / matrix spike duplicate (MS/MSD)); *- = LCS and/or LCSD is outside acceptance limits, low biased. laboratory control sample (LCS) and duplicate (LCSD); *+ = LCS and/or LCSD is outside acceptance limits, high biased; cn = Refer to Case Narrative for further detail; I = Value is estimated maximum possible concentration; ^2 = Calibration Blank is outside acceptance limits; F2 = MS/MSD RPD exceeds control limits.

**TABLE S5.** PFAS compounds detected in pre-stabilized and post-stabilized sludge sample by the three laboratories.

| Lab | Pre-stabilized sludge | Post-stabilized sludge (biosolids) |
| --- | --- | --- |
| 1 | 5:3 FTCA, PFHxA, PFDA, PFOS | 5:3 FTCA, NMeFOSAA, NMeFOSE, PFHxA, PFDA, PFDoA, PFOS |
| 2 | PFBS, PFOS | 5:3 FTCA, 6:2 FTS, PFOA, PFDA, PFBS, PFOS |
| 3 | 5:3 FTCA, 7:3 FTCA, 6:2 FTS, 8:2 FTS, NMeFOSAA, NEtFOSAA, PFOSA, NMeFOSE, PFHxA, PFDA, PFUA, PFDoA | 5:3 FTCA, 7:3 FTCA, 6:2 FTS, 8:2 FTS, NMeFOSAA, NEtFOSAA, PFOSA, NMeFOSE, PFHxA, PFDA, PFUA, PFDoA,  PFOS |

**Table S6.** Wilcoxon Rank Sum Test Evaluation

| Pair | Pre-stabilized sludge | Post-stabilized sludge (biosolid) samples | | |
| --- | --- | --- | --- | --- |
|  | Lab 1 and Lab3 | Lab 1 and Lab2 | Lab 1 and Lab3 | Lab 2 and Lab3 |
| n | 6 | 7 | 15 | 5 |
| T+ | 0 | 9 | 10 | 2 |
| T- | 21 | 19 | 110 | 13 |
| W_stat_ | 0 | 9 | 10 | 2 |
| W_crit_ | 2 | 3 | 30 | 0 |
| Condition | W_stat_ <W_crit_ | W_stat_ >W_crit_ | W_stat_ <W_crit_ | W_stat_ >W_crit_ |
| Notes | Reject null, there is significant difference | Accept null, no significant difference | Reject null, there is significant difference | Accept null, no significant difference |

Notes: ‘n’ = number of pair of data points used in evaluation; ‘T+’ = sum of rank of all the positive differences; ‘T-’ = sum of rank of all the negative values; ‘Wstat’ = Wilcoxon ranked signed statistics; ‘Wcrit’ = Wilcoxon critical value.

**Table S7.** Lab 2 Limit of Quantification (LOQ)

| No. | Analyte | LOQ (μg/L) |
| --- | --- | --- |
| 1 | PFPrA | 9.5 |
| 2 | PFBA | 40.7 |
| 3 | PFPeA | 24.8 |
| 4 | PFHxA | 37.5 |
| 5 | PFHpA | 19.1 |
| 6 | PFOA | 23.5 |
| 7 | PFNA | 13.4 |
| 8 | PFDA | 17.2 |
| 9 | PFUnA | 18.4 |
| 10 | PFDoA | 24.2 |
| 11 | PFTrDA | 26.1 |
| 12 | PFTeDA | 53.4 |
| 13 | PFPrS | 40.1 |
| 14 | PFBS | 15.9 |
| 15 | PFPeS | 22.3 |
| 16 | PFHxS | 37.5 |
| 17 | PFHpS | 25.4 |
| 18 | PFOS | 15.9 |
| 19 | PFNS | 48.3 |
| 21 | PFDS | 27.3 |
| 22 | PFDoS | 63.6 |
| 23 | 4:2 FTS | 26.7 |
| 24 | 6:2 FTS | 43.9 |
| 25 | 8:2 FTS | 36.3 |
| 26 | PFOSA | 12.7 |
| 27 | NMeFOSA | 12.7 |
| 28 | NEtFOSA | 12.7 |
| 29 | NMeFOSAA | 21.0 |
| 30 | NEtFOSAA | 17.2 |
| 31 | NMeFOSE | 127.2 |
| 32 | NEtFOSE | 127.2 |
| 33 | HFPO-DA | 37.5 |
| 34 | ADONA | 31.8 |
| 34 | PFEESA | 10.8 |
| 35 | PFMPA | 30.5 |
| 36 | PFMBA | 15.9 |
| 37 | NFDHA | 28.6 |
| 38 | 9CL-PF3ONS | 19.1 |
| 39 | 11CL-PF3OUDS | 15.9 |
| 40 | 3:3 FTCA | 152.6 |
| 41 | 5:3 FTCA | 152.6 |
| 42 | 7:3 FTCA | 152.6 |
| 43 | 6:6PFPi | 1938.5 |
| 44 | 6:8PFPi | 8140.8 |
| 45 | 8:8PFPi | 3700.2 |
| 46 | 6:2diPAP | 814.1 |
| 47 | 8:2diPAP | 4522.6 |

**Table S8**. Lab 3 Limit of Detection and ILOQ

|  | LOD (ng/mL) | LOQ (ng/mL) |
| --- | --- | --- |
| PFBA | 0.24 | 0.80 |
| PFPeA | 0.12 | 0.40 |
| PFHxA | 0.06 | 0.20 |
| PFHpA | 0.06 | 0.20 |
| PFOA | 0.06 | 0.20 |
| PFNA | 0.06 | 0.20 |
| PFDA | 0.06 | 0.20 |
| PFUnA | 0.06 | 0.20 |
| PFDoA | 0.06 | 0.20 |
| PFTrDA | 0.06 | 0.20 |
| PFTA | 0.06 | 0.20 |
| PFBS | 0.06 | 0.20 |
| PFPeS | 0.06 | 0.20 |
| PFHxS | 0.06 | 0.20 |
| PFHpS | 0.06 | 0.20 |
| PFOS | 0.06 | 0.20 |
| PFNS | 0.06 | 0.20 |
| PFDS | 0.06 | 0.20 |
| PFDoS | 0.06 | 0.20 |
| 4:2 FTS | 0.24 | 0.80 |
| 6:2 FTS | 0.24 | 0.80 |
| 8:2 FTS | 0.24 | 0.80 |
| ADONA | 0.24 | 0.80 |
| 11Cl-PF3OUdS | 0.24 | 0.80 |
| HFPO-DA | 0.24 | 0.80 |
| 9Cl-PF3ONS | 0.24 | 0.80 |
| NMeFOSAA | 0.06 | 0.20 |
| NEtFOSAA | 0.06 | 0.20 |
| NMeFOSA | 0.06 | 0.20 |
| NEtFOSA | 0.06 | 0.20 |
| NMeFOSE | 0.60 | 2.00 |
| NEtFOSE | 0.60 | 2.00 |
| NFDHA | 0.12 | 0.40 |
| PFEESA | 0.12 | 0.40 |
| PFMPA | 0.12 | 0.40 |
| PFMBA | 0.12 | 0.40 |
| PFOSA | 0.06 | 0.20 |
| 6:2 diPAP | 0.12 | 0.40 |
| 8:2 diPAP | 0.12 | 0.40 |
| 6:2/8:2 diPAP | 0.12 | 0.40 |
| 3:3 FTCA | 0.29 | 0.96 |
| 5:3 FTCA | 1.44 | 4.80 |
| 7:3 FTCA | 1.44 | 4.80 |
